# A Novel Method to Construct 2-Aminobenzofurans via [4 + 1] Cycloaddition Reaction of In Situ Generated *Ortho*-Quinone Methides with Isocyanides

**DOI:** 10.3390/molecules27238538

**Published:** 2022-12-04

**Authors:** Huaxin Lin, Senling Tang, Yang Pan, Peng Liang, Xiaofeng Ma, Wei Jiao, Huawu Shao

**Affiliations:** 1 Natural Products Research Centre, Chengdu Institute of Biology, Chinese Academy of Sciences, Chengdu 610041, China; 2 University of Chinese Academy of Sciences, Beijing 100049, China

**Keywords:** 2-aminobenzofurans, isocyanides, *ortho*-quinone methides, [4 + 1] cycloaddition

## Abstract

A new approach for the synthesis of 2-aminobenzofurans has been described via Sc(OTf)_3_ mediated formal cycloaddition of isocyanides with the in situ generated *ortho*-quinone methides (*o*-QMs) from *o*-hydroxybenzhydryl alcohol. Notably, as a class of readily available and highly active intermediates, *o*-QMs were first used in the construction of benzofurans. This [4 + 1] cycloaddition reaction provides a straightforward and efficient methodology for the construction of 2-aminobenzofurans scaffold in good yield (up to 93% yield) under mild conditions.

## 1. Introduction

Benzofuran core, an important class of structural fragments, is widely distributed in natural products and biologically active compounds [1,2,3,4]. The benzofuran subunit is also present in a host of medicines, such as amiodarone, methoxypsoralen, dronedarone, etc [5]. Therefore, various methods for the preparation of benzofurans have been developed. As a special kind of functionalized benzofurans, 2-aminobenzofurans are of considerable interest and feature profound bioactivities, such as antifungal, P-glycoprotein inhibitors, anticancer activities, and tubulin polymerization inhibitors [6,7,8,9]. 

**Scheme 1:**
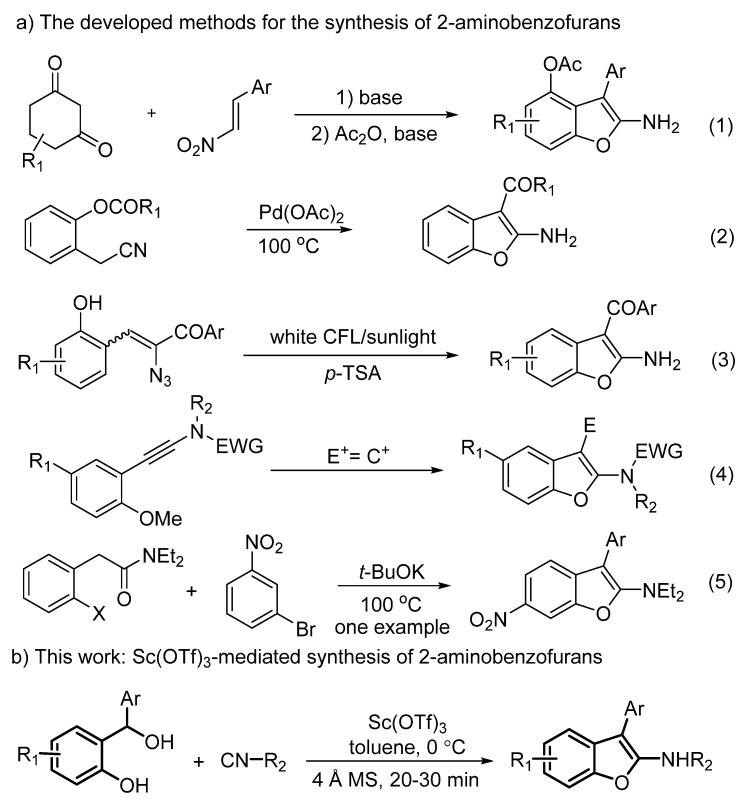
Strategies for the diversified synthesis of 2-aminobenzofurans.

Although such structures are important, only limited methods have been reported for accessing 2-aminobenzofurans and the structural diversity of the products is insufficient. For example, in 2005, Ishikawa’s group reported the synthesis of 2-aminobenzofurans from 1-aryl-2-nitroethylenes and cyclohexane-1,3-diones via a one-pot multistep strategy, but only moderate yield can be obtained. Moreover, unsymmetrical cyclohexane-1,3-diones have poor regiochemistry (Scheme 1a, Equation (1)) [10]. Soon after, Ohe and co-workers provided a new method to obtain 2-aminobenzofurans through palladium-catalyzed intramolecular cycloisomerization of 2-(cyanomethyl) phenyl ester; however, the substrate range is relatively limited (Scheme 1a, Equation (2)) [11]. In addition, Maurya’s group also demonstrated the synthesis of very similar products (3-acyl-2-aminobenzofurans) via visible light-triggered intramolecular cyclization of α-azidochalcones (Scheme 1a, Equation (3)) [12]. In 2013, Cao’s group developed a method for the synthesis of 3-alkyl- or 3-allenyl-2-amidobenzofurans by carbocation-induced electrophilic cyclization of *o*-anisole-substituted ynamides (Scheme 1a, Equation (4)) [13]. In this method, the substituent on the benzene ring is fixed at the 5 position, and at least one electron withdrawing substituent is required on nitrogen. Finally, Kumar et al. reported strong base (*t*BuOK) mediated synthesis of 3-phenylbenzofuran-2-amines (one example) (Scheme 1a, Equation (5)) [14]. While those methods allow 2-aminobenzofurans to be obtained in an efficient way, new methods that can access a variety of structural skeletons under mild reaction conditions and from simple starting materials are still highly desired (Scheme 1b).

In recent years, *ortho*-quinone methides (*o*-QMs) [15,16,17,18,19,20,21,22], a versatile class of building blocks, have been widely used in organic synthesis. *o*-QMs could be generated from *o*-hydroxybenzhydryl alcohol derivatives and directly participate in various [4 + *n*] (*n* = 2, 3) cycloadditions [23,24,25,26,27]. The [4 + 1] cycloadditions involved in *o*-QMs, however, are only developed for the construction of 2,3-dihydrobenzofuran skeletons and have never been used to synthesize benzofurans [28,29,30,31,32,33,34], let alone 2-aminobenzofurans. 

## 2. Results

Our continuous interest in cycloaddition [35,36,37,38,39,40] led us to envision that the reaction of *o*-QMs with isocyanides [41,42,43,44,45,46] would achieve the benzofuran motifs via an intermolecular formal [4 + 1] cycloaddition. To test the feasibility of our hypothesis, we chose *o*-hydroxybenzhydryl alcohol **1a** and *p*-nitrophenyl isocyanide **2a** as the model substrates to optimize the reaction conditions (Table 1).

The initial experiment was conducted in CH_2_Cl_2_ in the presence of various Brønsted acids, such as benzoic acid, TsOH and TfOH, at room temperature. It was observed that, except for benzoic acid, which only offered a trace amount of the desired product, both TsOH and TfOH provided the cycloaddition product **3a** in roughly the same yield, even though the yield was relatively low (entries 1–3). Considering that isocyanide could be hydrolyzed under fairly strong acidic conditions [47], we replaced Brønsted acids with Lewis acids to further optimize reaction conditions. A range of Lewis acids, such as BF_3_·Et_2_O, InCl_3_, and Sc(OTf)_3_, were then screened (entries 4–6). Among them, the desired cycloaddition product **3a** could be obtained with 53% isolated yield when 0.5 equiv. of Sc(OTf)_3_ was employed. To further improve the yield, different solvents, including THF, MeCN, and toluene, were also examined (entries 7−10). Toluene proved to be the best solvent for this transformation. 

Encouraged by these results, we investigated the effect of the loading of Sc(OTf)_3_. It was found that, when we increased the loading of Sc(OTf)_3_ from 0.5 equiv. to 1.0 equiv., the yield of **3a** was improved to 75% (entry 11). However, when 1.2 equiv. of Sc(OTf)_3_ was used, the yield was reduced slightly (entry 12). It is noteworthy that the cycloaddition product **3a** could be improved to 81% yield (entry 13) when the reaction was performed at 0 °C, but further cooling the temperature to −10 °C led to the yield’s reduction to 69% (entry 14). Lastly, the addition a small number of 4 Å MS could increase the yield of **3a** to 87% (entry 15).

**Scheme 2:**
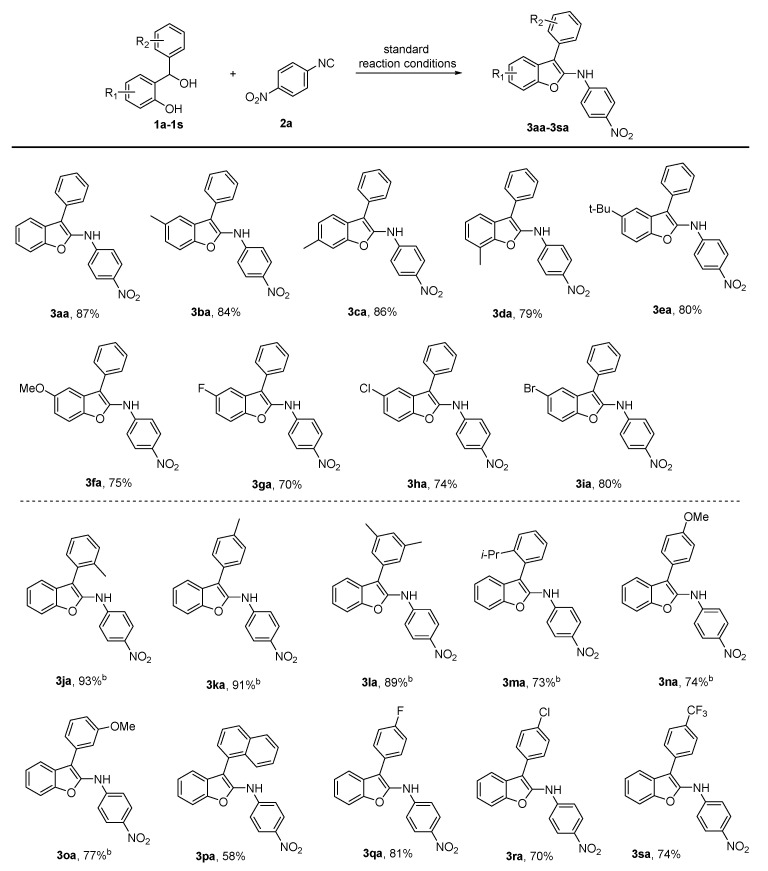
Scope of *o*-hydroxybenzhydryl alcohols. Standard reaction conditions: **1** (0.1 mmol), **2a** (0.2 mmol), Sc(OTf)_3_ (0.1 mmol), 4 Å MS (50 mg), dry toluene (1 mL), at 0 °C, 30 min. ^b^ 20 min.

With the optimized conditions in hand, a number of 2-aminobenzofurans were successfully obtained in moderate to excellent yields within 30 min through the formal [4 + 1] cycloaddition of *o*-hydroxybenzhydryl alcohol (**1a**–**1s**) and *p*-nitrophenyl isocyanide **2a** (Scheme 2). As shown in Scheme 2, both electron-donating substituents (**3ba**–**3fa**) and electron-deficient substituents (**3ga**–**3ia**) on the phenol were well tolerated in this formal [4 + 1] cycloaddition reaction and afforded the desired products in 70% to 84% yields. Obviously, the position of the substituents on phenol moiety had little influence on the reaction (**3ba** and **3ca**). The structure of products was unambiguously confirmed by single-crystal X-ray analysis of **3ia** (please see Supplementary Materials). Next, different substitutions on the benzyl phenol moiety were examined. We found that methyl substitution at the *ortho*-, *meta*- and *para*- of benzyl alcohol moiety can afford the corresponding products (**3ja**, **3ka**, and **3la**) good to excellent yields. Strong electron-donating substituent (methoxy) in a different position was also converted smoothly into the desired 2-aminobenzofurans (**3na** and **3oa**). Notably, the benzyl alcohol with high steric hindrance substitutions at *ortho*-position also efficiently underwent the formal [4 + 1] addition to provide corresponding products (**3ma** and **3pa**) in 73% and 58% yields, respectively. Electron-withdrawing substituents, including F, Cl, and CF_3_, were also suitable for this transformation, providing the 2-aminobenzofurans with good results (**3qa**–**3sa**). From the above results, it can be concluded that both strong electron-donating substituents and electron-withdrawing substituents at the benzyl alcohol slightly reduce the yield; the yield of the product decreases slightly when high steric hindrance substitutions at *ortho*-position of the benzyl alcohol take place.

**Scheme 3:**
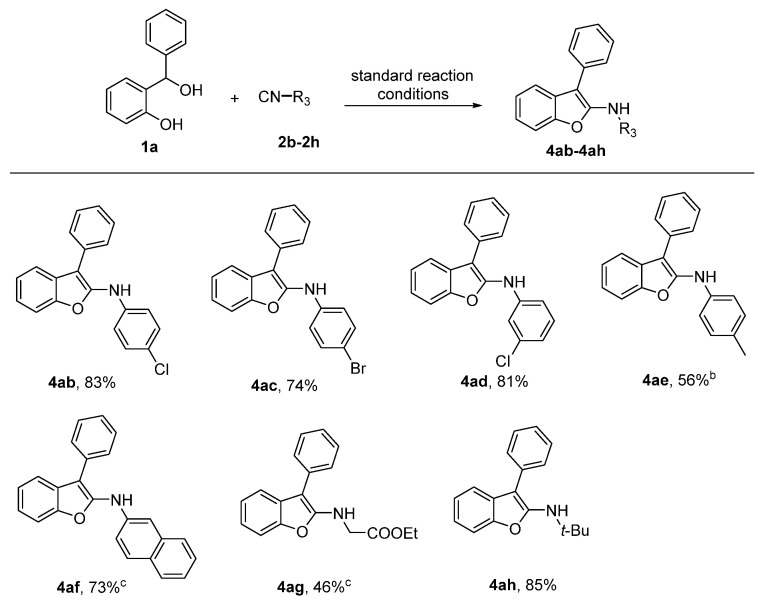
Scope of isocyanides. Standard reaction conditions: **1a** (0.1 mmol), 2 (0.2 mmol), Sc(OTf)_3_ (0.1 mmol), 4 Å MS (50 mg), dry toluene (1 mL), at 0 °C, 20 min. ^b^ Reaction time is 3 min. ^c^ Reaction time is 10 min.

We further evaluated the substrate scope of isocyanides (Scheme 3). A series of phenylisocyanides, with electron-withdrawing substituents at *para*- and *meta*-positions of the benzene ring, were smoothly converted to the corresponding products (**4ab**–**4ad**). However, for methyl substituted phenyl isocyanides, the yield decreased (**4ae**). Therefore, it can be inferred that electron-withdrawing substituents on phenyl isocyanides are beneficial to the formation of the product. *β*-Naphthyl isocyanide were employed in the transformation, offering the corresponding 2-aminobenzofurans in 83% yield (**4af**). Notably, alkyl isocyanides, including ethyl isocyanoacetate and *tert*-butyl isocyanide, could also smoothly transform to the desired cycloaddition products in 46% and 85% yields, respectively (**4ag** and **4ah**), which enriched the diversity of structural skeletons.

**Scheme 4:**
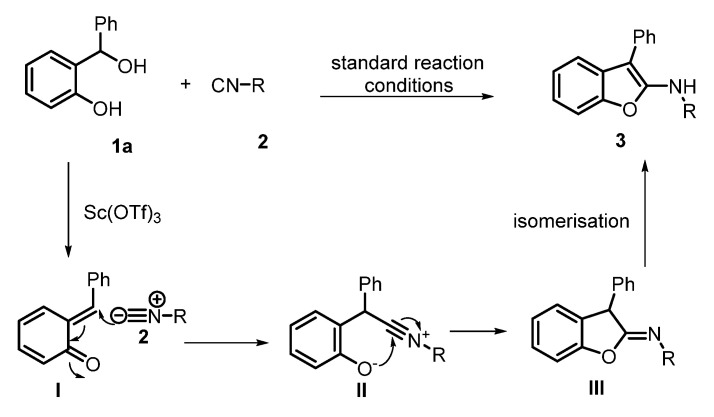
Plausible reaction mechanism.

According to previous reports on Sc(OTf)_3_-triggered transformation of *o*-hydroxybenzhydryl alcohol [48,49,50,51], a plausible mechanism for the [4 + 1] cycloaddition was proposed (Scheme 4), the nucleophilic addition of the isocyanides to *o*-QMs (**Ⅰ**) generated in situ from *o*-hydroxybenzyl alcohol **1a**, which formed intermediate **Ⅱ**. Subsequently, **Ⅱ** undergoes intramolecular cyclization producing the intermediate **Ⅲ**, which isomerised to the desired 2-aminobenzofurans **3**.

Given that 2-aminobenzofurans have been proven to have a variety of biological activities, we decide to conduct in silico researches of the synthesized 2-aminobenzofurans to evaluate their drug-likeness, which were carried out using the SwissADME platform [52]. Satisfyingly, except for compounds **3ea**, **3ma**, **3pa**, and **4ab**–**4af**, other compounds were found to have good obedience (100%) with two drug-likeness filters (Lipinski [53] and Veber [54]) (Table 2). In addition, some substituted (Cl, F, Br, CH_3_, OCH_3_) 2-aminobenzofurans’ pharmacokinetic properties were predicted through admetSAR [55], and it was found that these products showed a great range of average ADMET score [56,57] (0.68–0.74) with regard to human intestinal absorption, blood–brain barrier penetration, Caco-2 permeability, Ames mutagenicity, carcinogenicity, and acute oral toxicity class (Table 3). Finally, taking **4ae** as an example, we predicted its possible molecular targets using SwissTargetPrediction [58,59]. The results show that it can act on multiple targets, such as nuclear receptor, family A-G protein-coupled receptor, etc., and the probability of prediction is around 10%**.**

In summary, we have developed a novel and efficient method for the acquisition of 2-aminobenzofuran derivatives via Sc(OTf)_3_-promoted [4 + 1] cycloaddition reaction of isocyanides with the in situ generated *ortho*-quinone methides (*o*-QMs) under mild conditions. In addition, *o*-QMs were first successfully used in this transformation and its advantage of this transformation is the simplicity of the reaction and the increased variety of 2-aminobenzofurans. Further exploration of the construction of other heterocyclics from *o*-QMs and applications of this product is in progress.

## 3. Experimental

### 3.1. General Procedures

Unless otherwise noted, reagents were commercially available and were used without further purification. A 4 Å molecular sieve was pre-dried in an oven at 200 °C for 3 h Thin-layer chromatography (TLC) was performed using silica gel GF254 precoated plates (0.20 mm thickness). Visualization on TLC was achieved by UV light (254 nm). Column chromatography was performed on silica gel 90, 200–300 mesh. ^1^H NMR and ^13^C NMR spectra were recorded at 25 °C on a Bruker Avance 400 spectrometer (^1^H: 400 MHz and ^13^C: 101 MHz). ^1^H NMR chemical shifts are reported in ppm (δ) relative to tetramethylsilane (TMS) with the solvent resonance employed as the internal standard (CDCl_3_, δ 7.26 ppm; DMSO-*d*_6_, δ 2.5 ppm). ^13^C NMR chemical shifts were determined relative to the signal of the solvent: CDCl_3_ at δ 77.00 ppm, DMSO-*d*_6_ at δ 39.5 ppm. Data for 1H and ^13^C NMR were recorded as follows: chemical shift (δ, ppm), multiplicity (s = singlet, d = doublet, t = triplet, m = multiplet, q = quartet, dd = doublet of doublets, dt = doublet of triplets, td = triplet of doublets), coupling constants (Hz), and integration. ESI-HRMS spectra were recorded on a BioTOF Q instrument. Infrared (IR) spectra are obtained by the use of Spectrum One and expressed in wave number (cm^−1^). *o*-hydroxybenzhydryl alcohols **1a**–**1s** [60] and isocyanides **2a**–**2f** [51] were synthesized according to the previously reported.

### 3.2. Typical Procedure for Synthesis of **3aa**

To a solution of *p*-nitrophenyl isocyanide **2a** (0.2 mmol, 30 mg) in toluene (0.5 mL), we immediately added the *o*-hydroxybenzhydryl alcohols **1a** (0.1 mmol, 20 mg), Sc(OTf)_3_ (0.1 mmol, 49 mg) in toluene (0.5 mL) under N_2_ in a Schlenck tube. The reaction mixture was stirred at 0 °C for 30 min. Upon completion, the reaction mixture was quenched with water, and then extracted with EtOAc and washed with brine. The combined organic phase was dried over anhydrous Na_2_SO_4_ and the solvent was evaporated under vacuum. The crude product was purified using flash chromatography column eluting with (petroleum ether:ethyl acetate = 15:1) to obtain the product **3aa**.

Detailed physicochemical properties of novel 2-aminobenzofuran derivatives:

*N-(4-Nitrophenyl)-3-phenylbenzofuran-2-amine* (**3aa**): Petroleum ether: ethyl acetate = 10:1, Rf = 0.5, yield: 87%, red solid, mp 123 °C.IR: 3309, 1639, 1589, 1494, 1393, 1311, 1242, 1190, 1114. ^1^H NMR (400 MHz, Chloroform-*d*) δ 8.16 (d, *J* = 8.8 Hz, 2H), 7.71 (d, *J* = 7.0 Hz, 1H), 7.58 (d, *J* = 7.3 Hz, 2H), 7.50 (q, *J* = 7.4 Hz, 3H), 7.37 (dt, *J* = 13.6, 6.9 Hz, 3H), 7.02 (d, *J* = 8.8 Hz, 2H), 6.66 (s, 1H). ^13^C NMR (101 MHz, CDCl_3_) δ 151.2, 148.3, 145.3, 141.0, 131.2, 129.6, 128.2, 128.1, 127.6, 126.0, 124.3, 123.6, 119.5, 114.6, 111.1, 108.1. ESI-HRMS: *m*/*z* calcd for C_20_H_15_N_2_O_3_ [M + H]^+^: 331.1077, found: 331.1075.
*5-Methyl-N-(4-nitrophenyl)-3-phenylbenzofuran-2-amine* (**3ba**): Petroleum ether: ethyl acetate = 10:1, Rf = 0.6, yield: 84%, red solid, mp 108 °C. IR: 3368, 2922, 1586, 1492, 1323, 1239, 1192, 1110. ^1^H NMR (400 MHz, CDCl_3_) δ 8.14 (d, *J* = 9.1 Hz, 2H), 7.58–7.51 (m, 2H), 7.51–7.42 (m, 3H), 7.41–7.33 (m, 2H), 7.18–7.12 (dd, *J* = 8.4 Hz, 0.8Hz, 1H), 7.03–6.95 (m, 2H), 6.54 (s, 1H), 2.47 (s, 3H). ^13^C NMR (101 MHz, CDCl_3_) δ 149.6, 148.3, 145.3, 141.1, 133.1, 131.4, 129.2, 128.2, 128.0, 127.6, 126.0, 125.4, 119.4, 114.5, 110.6, 108.0, 21.5. ESI-HRMS: *m*/*z* calcd for C_21_H_17_N_2_O_3_ [M + H]^+^: 345.1234, found: 345.1239.
*6-Methyl-N-(4-nitrophenyl)-3-phenylbenzofuran-2-amine* (**3ca**): Petroleum ether: ethyl acetate = 10:1, Rf = 0.6, yield: 86%, red solid, mp 109 °C. IR: 3360, 2920, 1596, 1496, 1322, 1304, 1248, 1188, 1109. ^1^H NMR (400 MHz, CDCl_3_) δ 8.13 (d, *J* = 9.1 Hz, 2H), 7.56 (dd, *J* = 11.8, 7.7 Hz, 3H), 7.46 (t, *J* = 7.6 Hz, 2H), 7.35 (t, *J* = 7.4 Hz, 1H), 7.31 (s, 1H), 7.14 (d, *J* = 7.9 Hz, 1H), 6.99–6.91 (m, 2H), 6.53 (s, 1H), 2.51 (s, 3H). ^13^C NMR (101 MHz, CDCl_3_) δ 151.7, 148.7, 144.4, 141.0, 134.8, 131.4, 129.2, 128.1, 127.6, 126.0, 125.3, 124.8, 119.2, 114.4, 111.4, 109.0, 21.7. ESI-HRMS: *m*/*z* calcd for C_21_H_17_N_2_O_3_ [M + H]^+^: 345.1234, found: 345.1234.
*7-Methyl-N-(4-nitrophenyl)-3-phenylbenzofuran-2-amine* (**3da**): Petroleum ether: ethyl acetate = 10:1, Rf = 0.6, yield: 79%, red solid, mp 141 °C. IR: 3364, 2923, 1591, 1501, 1385, 1325, 1248, 1183, 1110. ^1^H NMR (400 MHz, CDCl_3_) δ 8.19–8.11 (m, 2H), 7.59–7.51 (m, 3H), 7.47 (t, *J* = 7.6 Hz, 2H), 7.36 (t, *J* = 7.4 Hz, 1H), 7.22 (t, *J* = 7.6 Hz, 1H), 7.16 (d, *J* = 7.3 Hz, 1H), 7.03–6.96 (m, 2H), 6.60 (s, 1H), 2.56 (s, 3H). ^13^C NMR (101 MHz, CDCl_3_) δ 150.2, 148.4, 144.9, 141.0, 131.4, 129.2, 128.2, 127.6, 127.5, 126.0, 125.4, 123.6, 121.4, 117.1, 114.5, 108.6, 15.0. ESI-HRMS: *m*/*z* calcd for C_21_H_17_N_2_O_3_ [M + H]^+^: 345.1234, found: 345.1255.
*5-(tert-butyl)-N-(4-nitrophenyl)-3-phenylbenzofuran-2-amine* (**3ea**): Petroleum ether: ethyl acetate = 15:1, Rf = 0.7, yield: 80%, red solid, mp 97 °C. IR: 3356, 2960, 1591, 1503, 1340, 1285, 1186, 1111. ^1^H NMR (400 MHz, Chloroform-*d*) δ 8.13 (d, *J* = 9.0 Hz, 2H), 7.68 (s, 1H), 7.56 (d, *J* = 7.2 Hz, 2H), 7.49 (t, *J* = 7.5 Hz, 2H), 7.43 (s, 2H), 7.38 (d, *J* = 7.2 Hz, 1H), 6.98 (d, *J* = 9.0 Hz, 2H), 6.62 (s, 1H), 1.41 (s, 9H). ^13^C NMR (101 MHz, CDCl_3_) δ 149.5, 148.5, 146.8, 145.3, 141.0, 131.4, 129.3, 128.3, 127.6, 126.0, 122.2, 115.7, 114.5, 110.5, 108.7, 34.9, 31.9. ESI-HRMS: *m*/*z* calcd for C_24_H_23_N_2_O_3_ [M + H]^+^: 387.1703, found: 387.1704.
*5-Methoxy-N-(4-nitrophenyl)-3-phenylbenzofuran-2-amine* (**3fa**): Petroleum ether: ethyl acetate = 10:1, Rf = 0.4, yield: 75%, red solid, mp 165 °C. IR: 3371, 2931, 1586, 1479, 1322, 1296, 1225, 1191, 1152, 1110. ^1^H NMR (400 MHz, CDCl_3_) δ 8.14 (d, *J* = 9.1 Hz, 2H), 7.56–7.51 (m, 2H), 7.48 (t, *J* = 7.6 Hz, 2H), 7.42–7.34 (m, 2H), 7.12 (d, *J* = 2.5 Hz, 1H), 7.05–6.97 (m, 2H), 6.92 (dd, *J* = 8.9, 2.6 Hz, 1H), 6.62 (s, 1H), 3.85 (s, 3H). ^13^C NMR (101 MHz, CDCl_3_) δ 156.6, 148.1, 146.0, 146.0, 141.1, 131.3, 129.3, 128.7, 128.2, 127.6, 126.0, 114.6, 112.3, 111.6, 107.9, 102.5, 56.0. ESI-HRMS: *m*/*z* calcd for C_21_H_17_N_2_O_4_ [M + H]^+^: 361.1183, found: 361.1187.
*5-Fluoro-N-(4-nitrophenyl)-3-phenylbenzofuran-2-amine* (**3ga**): Petroleum ether: ethyl acetate = 10:1, Rf = 0.6, yield: 70%, red solid, mp 141 °C. IR: 3343, 1586, 1502, 1476, 1325, 1311, 1242, 1195, 1140, 1110. ^1^H NMR (400 MHz, DMSO-*d*_6_) δ 10.05 (s, 1H), 8.10 (d, *J* = 9.0 Hz, 2H), 7.62 (dd, *J* = 10.3, 5.9 Hz, 3H), 7.53–7.41 (m, 3H), 7.36 (t, *J* = 7.3 Hz, 1H), 7.17 (td, *J* = 9.2, 2.3 Hz, 1H), 7.04 (d, *J* = 9.1 Hz, 2H). ^13^C NMR (101 MHz, DMSO-*d*_6_) δ159.6 (d, *J* = 232.3 Hz), 150.0, 148.1, 147.3, 140.1, 131.0, 129.5, 129.3, 128.4, 127.9, 126.2, 115.1, 112.7, 118.0 (d, *J* = 20.2 Hz), 108.3, 105.6, 105.3. ^19^F NMR (376 MHz, DMSO-*d*_6_) δ −119.35. ESI-HRMS: *m*/*z* calcd for C_20_H_14_FN_2_O_3_ [M + H]^+^: 349.0983, found: 349.0992.
*5-Chloro-N-(4-nitrophenyl)-3-phenylbenzofuran-2-amine* (**3ha**): Petroleum ether: ethyl acetate = 10:1, Rf = 0.6, yield: 74%, red solid, mp 198 °C. IR: 3366, 1588, 1500, 1384, 1325, 1234, 1109. ^1^H NMR (400 MHz, DMSO-*d*_6_) δ 10.08 (s, 1H), 8.10 (d, *J* = 9.1 Hz, 2H), 7.67 (d, *J* = 2.0 Hz, 1H), 7.65 (d, *J* = 8.7 Hz, 1H), 7.60 (d, *J* = 7.4 Hz, 2H), 7.49 (t, *J* = 7.6 Hz, 2H), 7.37 (td, *J* = 6.3, 5.5, 2.7 Hz, 2H), 7.05 (d, *J* = 9.2 Hz, 2H). ^13^C NMR (101 MHz, DMSO-*d*_6_) δ 149.9, 149.6, 147.9, 140.1, 130.8, 129.9, 129.6, 128.5, 128.5, 128.0, 126.2, 124.4, 118.9, 115.1, 113.2, 107.6. ESI-HRMS: *m*/*z* calcd for C_20_H_14_ClN_2_O_3_ [M + H]^+^: 365.0687, found: 365.0678.
*5-Bromo-N-(4-nitrophenyl)-3-phenylbenzofuran-2-amine* (**3ia**): Petroleum ether: ethyl acetate = 10:1, Rf = 0.6, yield: 80%, red solid, mp 210 °C. IR: 3367, 1585, 1504, 1468, 1324, 1232, 1109. ^1^H NMR (400 MHz, DMSO-*d*_6_) δ 10.08 (s, 1H), 8.09 (d, *J* = 9.1 Hz, 2H), 7.88 (d, *J* =1.2 Hz, 1H), 7.59 (d, *J* = 7.3 Hz, 3H), 7.54–7.44 (m, 3H), 7.36 (t, *J* = 7.3 Hz, 1H), 7.04 (d, *J* = 9.1 Hz, 2H). ^13^C NMR (101 MHz, DMSO-*d*_6_) δ 149.9, 149.9, 147.7, 140.1, 130.8, 130.5, 129.6, 128.5, 128.0, 127.1, 126.2, 121.8, 116.4, 115.1, 113.6, 107.4. ESI-HRMS: *m*/*z* calcd for C_20_H_13_BrN_2_NaO_3_ [M + Na]^+^: 431.0002, found: 430.9998.
*N-(4-Nitrophenyl)-3-(o-tolyl)benzofuran-2-amine* (**3ja**): Petroleum ether: ethyl acetate = 10:1, Rf = 0.6, yield: 93%, red solid, mp 145 °C. IR: 3347, 2925, 1593, 1503, 1327, 1248, 1169, 1112. ^1^H NMR (400 MHz, CDCl_3_) δ 8.17–8.11 (m, 2H), 7.54–7.49 (m, 1H), 7.37–7.27 (m, 7H), 7.08–7.02 (m, 2H), 6.44 (s, 1H), 2.24 (s, 3H). ^13^C NMR (101 MHz, CDCl_3_) δ 151.0, 147.6, 145.8, 141.1, 137.6, 130.9, 130.5, 129.8, 129.1, 128.4, 126.4, 125.9, 123.8, 123.4, 119.6, 114.7, 110.9, 106.2, 20.2. ESI-HRMS: *m*/*z* calcd for C_21_H_17_N_2_O_3_ [M + H]^+^: 345.1234, found: 345.1234.
*N-(4-Nitrophenyl)-3-(p-tolyl)benzofuran-2-amine* (**3ka**): Petroleum ether: ethyl acetate = 10:1, Rf = 0.6, yield: 91%, red solid, mp 138 °C. IR: 3359, 2920, 1591, 1524, 1384, 1248, 1175, 1109. ^1^H NMR (400 MHz, CDCl_3_) δ 8.14 (d, *J* = 9.1 Hz, 2H), 7.72–7.64 (m, 1H), 7.49 (d, *J* = 7.4 Hz, 1H), 7.45 (d, *J* = 8.0 Hz, 2H), 7.37–7.30 (m, 2H), 7.28 (d, *J* = 7.9 Hz, 2H), 6.99 (d, *J* = 9.1 Hz, 2H), 6.58 (s, 1H), 2.41 (s, 3H). ^13^C NMR (101 MHz, CDCl_3_) δ 151.2, 148.4, 145.0, 141.0, 137.5, 130.0, 128.1, 128.1, 128.1, 126.0, 124.2, 123.5, 119.6, 114.5, 111.1, 108.3, 21.3. ESI-HRMS: *m*/*z* calcd for C_21_H_16_N_2_NaO_3_ [M + Na]^+^: 367.1053, found: 367.1054.
*3-(3,5-Dimethylphenyl)-N-(4-nitrophenyl)benzofuran-2-amine* (**3la**): Petroleum ether: ethyl acetate = 12:1, Rf = 0.6, yield: 89%, red solid, mp 92 °C. IR: 3342, 2922, 1592, 1502, 1384, 1326, 1182, 1111. ^1^H NMR (400 MHz, CDCl_3_) δ 8.15 (d, *J* = 9.1 Hz, 2H), 7.67 (dd, *J* = 6.0, 2.7 Hz, 1H), 7.49 (dd, *J* = 6.5, 2.2 Hz, 1H), 7.33 (dt, *J* = 6.6, 4.7 Hz, 2H), 7.16 (s, 2H), 7.03 (d, *J* = 9.2 Hz, 3H), 6.64 (s, 1H), 2.37 (s, 6H). ^13^C NMR (101 MHz, CDCl_3_) δ 151.1, 148.2, 145.3, 141.0, 138.9, 131.0, 129.4, 128.2, 126.0, 124.0, 123.5, 119.6, 114.7, 111.0, 107.7, 21.5. ESI-HRMS: *m*/*z* calcd for C_22_H_18_N_2_NaO_3_ [M + Na]^+^: 381.1210, found: 381.1206.
*3-(2-Isopropylphenyl)-N-(4-nitrophenyl)benzofuran-2-amine* (**3ma**): Petroleum ether: ethyl acetate = 12:1, Rf = 0.6, yield: 73%, red solid, mp 170 °C. IR: 3319, 2961, 1642, 1592, 1499, 1384, 1323, 1306, 1237, 1186, 1112. ^1^H NMR (400 MHz, Chloroform-*d*) δ 8.14 (d, *J* = 9.0 Hz, 2H), 7.52 (d, *J* = 8.0 Hz, 1H), 7.50–7.40 (m, 2H), 7.32–7.28 (m, 5H), 7.05 (d, *J* = 9.0 Hz, 2H), 6.49 (s, 1H), 3.02 (hept, *J* = 6.6 Hz, 1H), 1.18 (d, *J* = 6.8 Hz, 3H), 1.06 (d, *J* = 6.8 Hz, 3H). ^13^C NMR (101 MHz, CDCl_3_) δ 150.9, 148.9, 147.9, 146.0, 141.1, 130.9, 129.8, 129.0, 128.3, 126.3, 126.3, 125.9, 123.8, 123.5, 119.3, 114.6, 110.9, 106.3, 30.1, 24.5, 24.1. ESI-HRMS: *m*/*z* calcd for C_23_H_21_N_2_O_3_ [M + H]^+^: 373.1547, found: 373.1547.
*3-(4-Methoxyphenyl)-N-(4-nitrophenyl)benzofuran-2-amine* (**3na**): Petroleum ether: ethyl acetate = 10:1, Rf = 0.4, yield: 74%, red solid, mp 177 °C. IR: 3358, 2950, 1594, 1502, 1307, 1231, 1152, 1114. ^1^H NMR (400 MHz, CDCl_3_) δ 8.15 (d, *J* = 9.0 Hz, 2H), 7.70–7.63 (m, 1H), 7.48 (t, *J* = 7.1 Hz, 3H), 7.37–7.28 (m, 2H), 6.99 (dd, *J* = 11.2, 8.9 Hz, 4H), 6.50 (s, 1H), 3.85 (s, 3H). ^13^C NMR (101 MHz, CDCl_3_) δ 159.1, 151.2, 148.5, 144.7, 141.0, 129.4, 128.2, 126.0, 124.3, 123.4, 123.3, 119.6, 114.7, 114.4, 111.1, 108.4, 55.4. ESI-HRMS: *m*/*z* calcd for C_21_H_17_N_2_O_4_ [M + H]^+^: 361.1183, found: 361.1183.
*3-(3-Methoxyphenyl)-N-(4-nitrophenyl)benzofuran-2-amine* (**3oa**): Petroleum ether: ethyl acetate = 10:1, Rf = 0.4, yield: 77%, red solid, mp 107 °C. IR: 3315, 2920, 1591, 1501, 1325, 1309, 1239, 1183, 1110. ^1^H NMR (400 MHz, CDCl_3_) δ 8.15 (d, *J* = 9.1 Hz, 2H), 7.69 (dd, *J* = 6.5, 2.2 Hz, 1H), 7.50 (dd, *J* = 6.9, 1.9 Hz, 1H), 7.39 (t, *J* = 7.9 Hz, 1H), 7.36–7.28 (m, 2H), 7.13 (d, *J* = 7.7 Hz, 1H), 7.10 (t, *J* = 2.0 Hz, 1H), 7.03 (d, *J* = 9.1 Hz, 2H), 6.91 (dd, *J* = 8.2, 2.3 Hz, 1H), 6.63 (s, 1H), 3.81 (s, 3H). ^13^C NMR (101 MHz, CDCl_3_) δ 160.2, 151.1, 148.1, 145.4, 141.1, 132.5, 130.4, 128.0, 126.0, 124.2, 123.6, 120.5, 119.5, 114.7, 114.0, 112.8, 111.1, 107.5, 55.3. ESI-HRMS: *m/z* calcd for C_21_H_16_N_2_NaO_4_ [M + Na]^+^: 383.1002, found: 383.1004.
*3-(Naphthalen-1-yl)-N-(4-nitrophenyl)benzofuran-2-amine* (**3pa**): Petroleum ether: ethyl acetate = 10:1, Rf = 0.6, yield: 58%, red solid, mp 194 °C. IR: 3309, 2920, 1637, 1587, 1498, 1322, 1305, 1242, 1183, 1110. ^1^H NMR (400 MHz, CDCl_3_) δ 8.11 (d, *J* = 9.1 Hz, 2H), 7.99–7.91 (m, 2H), 7.83 (d, *J* = 8.4 Hz, 1H), 7.51–7.61 (m, 4H), 7.44 (t, *J* = 7.5 Hz, 1H), 7.36–7.31 (m, 1H), 7.29 (d, *J* = 6.7 Hz, 1H), 7.24 (d, *J* = 7.4 Hz, 1H), 7.06 (d, *J* = 9.1 Hz, 2H), 6.46 (s, 1H). ^13^C NMR (101 MHz, CDCl_3_) δ 151.0, 147.4, 146.8, 141.1, 134.1, 131.7, 129.6, 128.8, 128.8, 128.2, 128.1, 126.7, 126.4, 125.8, 125.8, 125.4, 123.7, 123.6, 119.7, 114.9, 110.9, 104.1. ESI-HRMS: *m*/*z* calcd for C_24_H_17_N_2_O_3_ [M + H]^+^: 381.1234, found: 381.1225.
*3-(4-Fluorophenyl)-N-(4-nitrophenyl)benzofuran-2-amine* (**3qa**): Petroleum ether: ethyl acetate = 10:1, Rf = 0.6, yield: 81%, red solid, mp 126 °C. IR: 3332, 2920, 1587, 1500, 1472, 1325, 1108. ^1^H NMR (400 MHz, DMSO-*d*_6_) δ 9.93 (s, 1H), 8.10 (d, *J* = 9.2 Hz, 2H), 7.72–7.58 (m, 4H), 7.40–7.28 (m, 4H), 7.00 (d, *J* = 9.2 Hz, 2H). ^13^C NMR (101 MHz, DMSO-*d*_6_) δ 161.7 (d, *J* = 252.5 Hz), 151.2, 150.5, 146.3, 139.9, 130.5, 130.4, 128.0, 127.8, 126.3, 124.8, 124.0, 119.7, 116.4 (d, *J* = 20.2 Hz), 114.8, 111.6, 108.0. ^19^F NMR (376 MHz, DMSO-*d*_6_) δ -114.37. ESI-HRMS: *m*/*z* calcd for C_20_H_14_FN_2_O_3_ [M + H]^+^: 349.0983, found: 349.0977.
*3-(4-Chlorophenyl)-N-(4-nitrophenyl)benzofuran-2-amine* (**3ra**): Petroleum ether: ethyl acetate = 10:1, Rf = 0.6, yield: 70%, red solid, mp 177 °C. IR: 3312, 2920, 1638, 1588, 1491, 1390, 1308, 1241, 1114. ^1^H NMR (400 MHz, CDCl_3_) δ 8.15 (d, *J* = 9.0 Hz, 2H), 7.64 (d, *J* = 7.1 Hz, 1H), 7.53–7.47 (m, 3H), 7.44 (d, *J* = 8.4 Hz, 2H), 7.39–7.29 (m, 2H), 7.00 (d, *J* = 9.0 Hz, 2H), 6.54 (s, 1H). ^13^C NMR (101 MHz, CDCl_3_) δ 151.2, 148.0, 145.3, 141.3, 133.5, 129.7, 129.5, 129.5, 127.7, 126.0, 124.5, 123.7, 119.3, 114.6, 111.2, 107.3. ESI-HRMS: *m*/*z* calcd for C_20_H_14_ClN_2_O_3_ [M + H]^+^: 365.0687, found: 365.0687.
*N-(4-Nitrophenyl)-3-(4-(trifluoromethyl)phenyl)benzofuran-2-amine* (**3sa**): Petroleum ether: ethyl acetate = 10:1, Rf = 0.6, yield: 74%, red solid, mp 226 °C. IR: 3311, 2967, 1590, 1311, 1307, 1272, 1239, 1112, 1066. ^1^H NMR (400 MHz, DMSO-*d*_6_) δ 10.09 (s, 1H), 8.13 (d, *J* = 9.2 Hz, 2H), 7.85 (s, 4H), 7.75 (dd, *J* = 6.6, 2.2 Hz, 1H), 7.63 (dd, *J* = 6.9, 1.8 Hz, 1H), 7.43–7.32 (m, 2H), 7.08 (d, *J* = 9.2 Hz, 2H). ^13^C NMR (101 MHz, DMSO-*d*_6_) δ 151.1, 149.9, 147.5, 140.1, 136.0, 129.1, 128.0, 127.7, 127.6, 126.4, 126.3, 126.3, 126.1, 124.8, 124.2, 123.4, 119.5, 115.2, 111.7, 106.5. ^19^F NMR (376 MHz, DMSO-*d*_6_) δ −60.99. ESI-HRMS: *m*/*z* calcd for C_21_H_13_F_3_N_2_NaO_3_ [M + Na]^+^: 421.0770, found: 421.0766.
*N-(4-Chlorophenyl)-3-phenylbenzofuran-2-amine* (**4ab**): Petroleum ether: ethyl acetate = 15:1, Rf = 0.6, yield: 83%, white solid, mp 102 °C. IR: 3371, 1636, 1594, 1479, 1384, 1238, 1183. ^1^H NMR (400 MHz, CDCl_3_) δ 7.62–7.67 (m, 1H), 7.58 (d, *J* = 7.2 Hz, 2H), 7.51–7.44 (m, 3H), 7.35 (t, *J* = 7.4 Hz, 1H), 7.28 (t, *J* = 3.6 Hz, 1H), 7.25 (d, *J* = 5.0 Hz, 1H), 7.25–7.20 (m, 2H), 7.00–6.93 (m, 2H), 6.13 (s, 1H). ^13^C NMR (101 MHz, CDCl_3_) δ 150.9, 147.9, 140.8, 132.0, 129.3, 129.2, 128.6, 128.1, 127.1, 126.1, 123.3, 123.2, 118.8, 117.3, 110.8, 104.5. ESI-HRMS: *m*/*z* calcd for C_20_H_15_ClNO [M + H]^+^: 320.0837, found: 320.0822.
*N-(4-Bromophenyl)-3-phenylbenzofuran-2-amine* (**4ac**): Petroleum ether: ethyl acetate = 15:1, Rf = 0.6, yield: 74%, white solid, mp 137 °C. IR: 3360, 1636, 1590, 1489, 1379, 1241, 1174. ^1^H NMR (400 MHz, CDCl_3_) δ 7.65 (dd, *J* = 5.4, 3.3 Hz, 1H), 7.57 (d, *J* = 7.4 Hz, 2H), 7.51–7.43 (m, 3H), 7.40–7.32 (m, 3H), 7.30–7.26 (m, 2H), 6.91 (d, *J* = 8.6 Hz, 2H), 6.12 (s, 1H). ^13^C NMR (101 MHz, CDCl_3_) δ 150.9, 147.7, 141.3, 132.2, 132.0, 129.12, 128.5, 128.1, 127.1, 123.3, 123.2, 118.8, 117.7, 113.3, 110.8, 104.7. ESI-HRMS: *m*/*z* calcd for C_20_H_15_BrNO [M + H]^+^: 364.0332, found: 364.0323.
*N-(3-Chlorophenyl)-3-phenylbenzofuran-2-amine* (**4ad**): Petroleum ether: ethyl acetate = 15:1, Rf = 0.6, yield: 81%, white solid, mp 134 °C. IR: 3359, 1637, 1593, 1492, 1378, 1242, 1173. ^1^H NMR (400 MHz, Chloroform-*d*) δ 7.68 (dd, *J* = 6.1, 2.9 Hz, 1H), 7.59 (d, *J* = 7.2 Hz, 2H), 7.48–7.52 (m, 3H), 7.37 (t, *J* = 7.4 Hz, 1H), 7.32–7.29 (m, 2H), 7.20 (t, *J* = 8.1 Hz, 1H), 7.05 (t, *J* = 1.9 Hz, 1H), 6.99–6.86 (m, 2H), 6.15 (s, 1H). ^13^C NMR (101 MHz, CDCl_3_) δ 151.0, 147.3, 143.6, 135.1, 131.9, 130.4, 129.2, 128.4, 128.2, 127.2, 123.4, 123.3, 121.1, 119.0, 115.9, 114.1, 110.9, 105.5. ESI-HRMS: *m*/*z* calcd for C_20_H_13_ClNO [M − H]^−^: 318.0680, found: 318.0685.
*3-Phenyl-N-(p-tolyl)benzofuran-2-amine* (**4ae**): Petroleum ether: ethyl acetate = 15:1, Rf = 0.6, yield: 56%, white solid, mp 92 °C. IR: 3380, 2925, 1608, 1517, 1384, 1196, 1071. ^1^H NMR (400 MHz, Chloroform-*d*) δ 7.69–7.56 (m, 3H), 7.52–7.42 (m, 3H), 7.33 (t, *J* = 7.4 Hz, 1H), 7.29–7.22 (m, 2H), 7.11 (d, *J* = 8.2 Hz, 1H), 6.99 (d, *J* = 8.4 Hz, 1H), 6.12 (s, 1H), 2.33 (s, 3H). ^13^C NMR (101 MHz, CDCl_3_) δ 150.8, 149.2, 139.5, 132.5, 129.9, 129.2, 128.9, 128.1, 126.8, 123.1, 122.6, 118.4, 116.7, 110.7, 102.6, 20.7. ESI-HRMS: *m*/*z* calcd for C_21_H_18_NO [M + H]^+^: 300.1383, found: 300.1380.
*N-(Naphthalen-2-yl)-3-phenylbenzofuran-2-amine* (**4af**): Petroleum ether: ethyl acetate = 15:1, Rf = 0.6, yield: 73%, White solid, mp 124 °C. IR: 3377, 1629, 1600, 1454, 1381, 1220, 1184. ^1^H NMR (400 MHz, DMSO-*d*_6_) δ 9.19 (s, 1H), 7.77 (d, *J* = 6.2 Hz, 1H), 7.75 (d, *J* = 5.3 Hz, 1H), 7.72–7.69 (m, 1H), 7.68–7.65 (m, 2H), 7.62 (d, *J* = 8.2 Hz, 1H), 7.56–7.60 (m, 1H), 7.45 (t, *J* = 7.7 Hz, 2H), 7.39–7.34 (m, 1H), 7.33–7.27 (m, 3H), 7.27–7.24 (m, 1H), 7.22–7.25 (m, 1H), 7.19 (d, *J* = 2.0 Hz, 1H). ESI-HRMS: *m/z* calcd for C_24_H_16_NO [M − H]^−^: 334.1226, found: 334.1239.
*Ethyl-(3-phenylbenzofuran-2-yl) glycinate* (**4ag**): Petroleum ether: ethyl acetate = 6:1, Rf = 0.5, yield: 46%, White solid, mp 96 °C. IR: 3349, 2927, 1734, 1612, 1463, 1393, 1206, 1122. ^1^H NMR (400 MHz, CDCl_3_) δ 7.62 (d, *J* = 7.2 Hz, 2H), 7.45–7.55 (m, 3H), 7.33 (d, *J* = 8.0 Hz, 1H), 7.30 (t, *J* = 7.5 Hz, 1H), 7.19 (t, *J* = 7.4 Hz, 1H), 7.12–7.06 (m, 1H), 5.05 (t, *J* = 5.6 Hz, 1H), 4.24 (q, *J* = 7.1 Hz, 2H), 4.18 (d, *J* = 5.9 Hz, 2H), 1.30 (t, *J* = 7.1 Hz, 3H). ^13^C NMR (101 MHz, CDCl_3_) δ 170.7, 153.7, 150.0, 133.2, 130.2, 129.2, 127.6, 126.0, 123.1, 120.6, 117.1, 109.9, 93.9, 61.6, 45.4, 14.2. ESI-HRMS: *m*/*z* calcd for C_18_H_18_NO_3_ [M + H]^+^: 296.1281, found: 296.1281.
*N-(tert-butyl)-3-phenylbenzofuran-2-amine* (**4ah**): Petroleum ether: ethyl acetate = 20:1, Rf = 0.6, yield: 85%, White solid, mp 108 °C. IR: 3367, 2967, 1606, 1458, 1379, 1210, 1015. ^1^H NMR (400 MHz, CDCl_3_) δ 7.53 (d, *J* = 7.0 Hz, 2H), 7.51 –7.45 (m, 3H), 7.37 (d, *J* = 7.9 Hz, 1H), 7.30 (t, *J* = 7.2 Hz, 1H), 7.18 (t, *J* = 7.2 Hz, 1H), 7.10 (t, *J* =7.2 Hz, 1H), 4.39 (s, 1H), 1.43 (s, 9H). ^13^C NMR (101 MHz, CDCl_3_) δ 155.2, 150.4, 133.7, 129.6, 129.2, 127.8, 125.9, 122.8, 120.6, 117.0, 110.0 97.0, 53.5, 30.6. ESI-HRMS: *m*/*z* calcd for C_18_H_20_NO [M + H]^+^: 266.1539, found: 266.1537.

### 3.3. X-ray Crystallographic Data of **3ia**

The crystal of **3ia** for XRD analysis was prepared by recrystallization from the DMSO (see the supporting information for details). CCDC 1914402 containing the supplementary crystallographic data can be obtained free of charge from the Cambridge Crystallographic Data Centre via www.ccdc.cam.ac.uk/data_request/cif (accessed on 5 July 2019). (remarks: The unit cell contains several **3ia** and DMSO, which are weakly clustered together, but this does not affect the structural characterization of compound **3ia**.)

## Figures and Tables

**Table 1:** Optimization of reaction conditions ^a^.

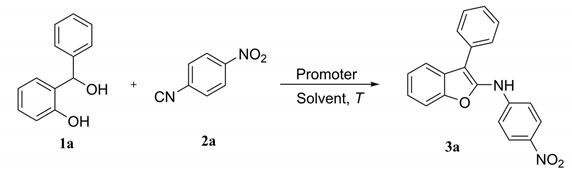			
Entry	Promoter	Solvent	Yield ^b^ (%)
1	TsOH	CH_2_Cl_2_	18
2	TfOH	CH_2_Cl_2_	15
3	benzoic acid	CH_2_Cl_2_	trace
4	BF_3_·Et_2_O	CH_2_Cl_2_	40
5	InCl_3_	CH_2_Cl_2_	23
6	Sc(OTf)_3_	CH_2_Cl_2_	53
7	Sc(OTf)_3_	THF	29
8	Sc(OTf)_3_	MeCN	37
9	Sc(OTf)_3_	DCE	50
10	Sc(OTf)_3_	toluene	60
11 ^c^	Sc(OTf)_3_	toluene	75
12 ^d^	Sc(OTf)_3_	toluene	71
13 ^c,e^	Sc(OTf)_3_	toluene	81
14 ^c,f^	Sc(OTf)_3_	toluene	69
15 ^c,e,g^	Sc(OTf)_3_	toluene	87

^a^ Reaction conditions: **1a** (0.1 mmol), **2a** (0.2 mmol), promoter (0.05 mmol), dry solvent (1 mL), at room temperature under N_2_, for 30 min. ^b^ Isolated yield. ^c^ Sc(OTf)_3_ (0.1 mmol). ^d^ Sc(OTf)_3_ (0.12 mmol). ^e^ The reaction was performed at 0 °C. ^f^ The reaction was performed at −10 °C. ^g^ 4 Å MS (50 mg) was employed.

**Table 2:** Physiochemical properties of the compounds predicted using SwissADME.

Compound Name	MW	nHetero Atoms	Rotatable Bonds	H-Bond Acceptor	H-Bond Donor	TPSA (Å sqr)	MlogP
**3aa**	330.34	5	4	3	1	70.99	4.03
**3ba**	344.37	5	4	3	1	70.99	3.44
**3ca**	344.37	5	4	3	1	70.99	3.44
**3da**	344.37	5	4	3	1	70.99	3.44
**3ea**	386.45	5	5	3	1	70.99	4.90 *
**3fa**	360.37	6	5	4	1	80.22	3.71
**3ga**	348.33	6	4	4	1	70.99	3.60
**3ha**	364.79	6	4	3	1	70.99	3.71
**3ia**	409.24	6	4	3	1	70.99	3.82
**3ja**	344.37	5	4	3	1	70.99	3.44
**3ka**	344.37	5	4	3	1	70.99	3.44
**3la**	358.40	5	4	3	1	70.99	3.66
**3ma**	372.42	5	5	3	1	70.99	4.69 *
**3na**	360.37	6	5	4	1	80.22	3.71
**3oa**	360.37	6	5	4	1	80.22	3.71
**3pa**	380.40	5	4	3	1	70.99	4.73 *
**3qa**	348.33	6	4	4	1	70.99	3.60
**3ra**	364.79	6	4	3	1	70.99	3.71
**3sa**	398.34	8	5	6	1	70.99	4.04
**4ab**	319.79	3	3	1	1	25.17	4.79 *
**4ac**	364.24	3	3	1	1	25.17	4.90 *
**4ad**	319.79	3	3	1	1	25.17	4.79 *
**4ae**	299.37	2	3	1	1	25.17	4.52 *
**4af**	335.31	2	3	1	1	25.17	4.99 *
**4ag**	295.34	4	6	3	1	51.47	2.80
**4ah**	265.36	2	3	1	1	25.17	3.79

(Lipinski: MW ≤ 500, MlogP ≤ 4.15, N or O ≤ 10, NH or OH ≤ 5; Veber: Rotatable bonds ≤ 10, TPSA ≤ 140). * The asterisk indicates that it is outside the standard range.

**Table 3:** ADMET score for human intestinal absorption, Caco-2 permeability, blood–brain barrier, carcinogenicity, Ames mutagenesis and acute oral toxicity, as predicted using admetSAR.

Compound Name	Human Intestinal Absorption	Blood Brain Barrier	Caco-2 Permeability	Ames Mutagenesis	Carcinogenicity	Acute Oral Toxicity	Average Score
**3ba**	0.9868	0.7500	0.7184	0.7400	0.5000	0.5118	0.7012
**3fa**	0.9848	0.7500	0.7869	0.7400	0.6020	0.5636	0.7379
**3ga**	0.9874	0.7500	0.7567	0.6800	0.5381	0.4706	0.6971
**3ha**	0.9860	0.7500	0.6451	0.7600	0.5881	0.5396	0.7115
**3ia**	0.9835	0.7500	0.5890	0.7000	0.5371	0.5325	0.6820
**3ka**	0.9868	0.7500	0.8320	0.7900	0.5000	0.5118	0.7284
**3na**	0.9848	0.7500	0.7356	0.7700	0.6020	0.5636	0.7343
**3qa**	0.9874	0.7500	0.7716	0.7600	0.5381	0.4706	0.7130
**3ra**	0.9860	0.7500	0.5674	0.8100	0.5881	0.5396	0.7069
**4ab**	0.9939	0.9000	0.6813	0.5500	0.5419	0.5439	0.7018
**4ac**	0.9925	0.9000	0.6529	0.6300	0.5929	0.5220	0.7151

## Data Availability

The details of the data supporting the report results in this research were included in the paper and Supplementary Materials.

## Supplementary Materials

The following supporting information can be downloaded at: https://www.mdpi.com/article/10.3390/molecules27238538/s1, Figure S1: X-ray molecular structure of **3ia**; Table S1: Crystal data and structure refinement for **3ia**; Figures S2–S56: NMR spectra of the products (**3aa**–**3sa**, **4ab**–**4ah**).
