# Supplementary material for: A Novel Method to Construct 2-Aminobenzofurans via [4 + 1] Cycloaddition Reaction of In Situ Generated Ortho-Quinone Methides with Isocyanides
Source: Molecules. 2022 Dec 4;27(23):8538. doi: 10.3390/molecules27238538 (PMC9737762; doi:10.3390/molecules27238538)
Supplement: Supplementary file 1 [file molecules-27-08538-s001.zip › molecules-2012203-supplementary.pdf]

Supporting information

A Novel Method to Construct 2-Aminobenzofurans via [4+1] Cycloaddition Reaction of In Situ Generated *Ortho*-Quinone Methides with Isocyanides

Huaxin Lin [1,2], Senling Tang [1,2], Yang Pan [1,2], Peng Liang [1,2], Xiaofeng Ma [1], Wei Jiao [1,*] and Huawu Shao [1,*]

[1] Natural Products Research Centre, Chengdu Institute of Biology, Chinese Academy of Sciences, Chengdu 610041, China

[2] University of Chinese Academy of Sciences, Beijing 100049, China

* Correspondence: jiaowei@cib.ac.cn (W.J.); shaohw@cib.ac.cn (H.S.)

Table of contents

1. X-ray Crystallographic data of **3ia**……………………………………………………………………………S2

2. NMR spectra of the products……………………………………………………………………………………S4

1. X-ray Crystallographic data of 3ia

The crystal of **3ia** suitable for XRD analysis was prepared by recrystallization from the DMSO. CCDC 1914402 containing the supplementary crystallographic data can be obtained free of charge from The Cambridge Crystallographic Data Centre via www.ccdc.cam.ac.uk/data_request/cif. (remarks: The unit cell contains several **3ia** and DMSO, which are weakly clustered together, but this does not affect the structural characterization of compound **3ia**.)

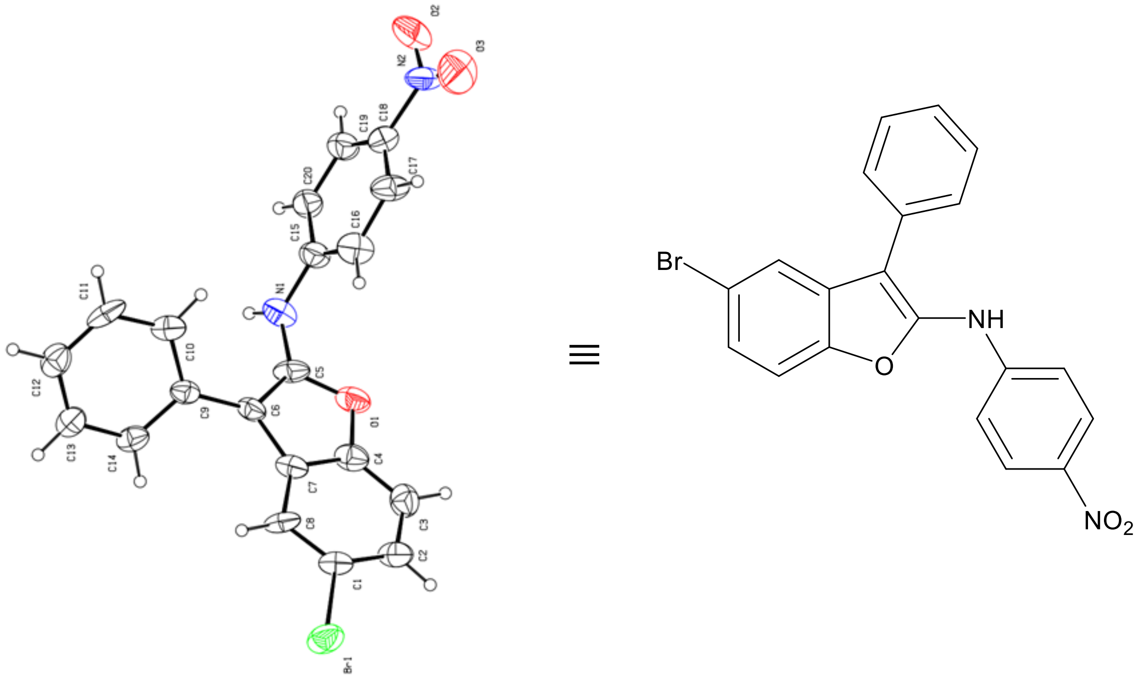

Figure S1. X-ray molecular structure of **3ia**

Table S1 Crystal data and structure refinement for mo_20190105_fence_01_0m_a.

Identification code	mo_20190105_fence_01_0m_a
Empirical formula	$C_{21}H_{16}BrN_2O_{3.5}S_{0.5}$
Formula weight	448.30
Temperature/K	297.15
Crystal system	triclinic
Space group	P-1

a/Å	17.968(4)
b/Å	18.005(4)
c/Å	18.552(4)
α/°	94.914(7)
β/°	95.055(7)
γ/°	90.091(8)
Volume/Å^3	5956(2)
Z	12
ρ_{calc}g/cm^3	1.500
μ/mm^{-1}	2.150
F(000)	2724.0
Crystal size/mm^3	0.21 × 0.17 × 0.06
Radiation	MoKα (λ = 0.71073)
2Θ range for data collection/°	4.424 to 46.978
Index ranges	-20 ≤ h ≤ 20, -20 ≤ k ≤ 20, -20 ≤ l ≤ 20
Reflections collected	71482
Independent reflections	17177 [R_{int} = 0.0776, R_{sigma} = 0.1081]
Data/restraints/parameters	17177/4/1533
Goodness-of-fit on F^2	1.317
Final R indexes [I>=2σ (I)]	R_1 = 0.1164, wR_2 = 0.3325
Final R indexes [all data]	R_1 = 0.1660, wR_2 = 0.3867
Largest diff. peak/hole / e Å^{-3}	1.11/-2.40

2. NMR spectra of the products

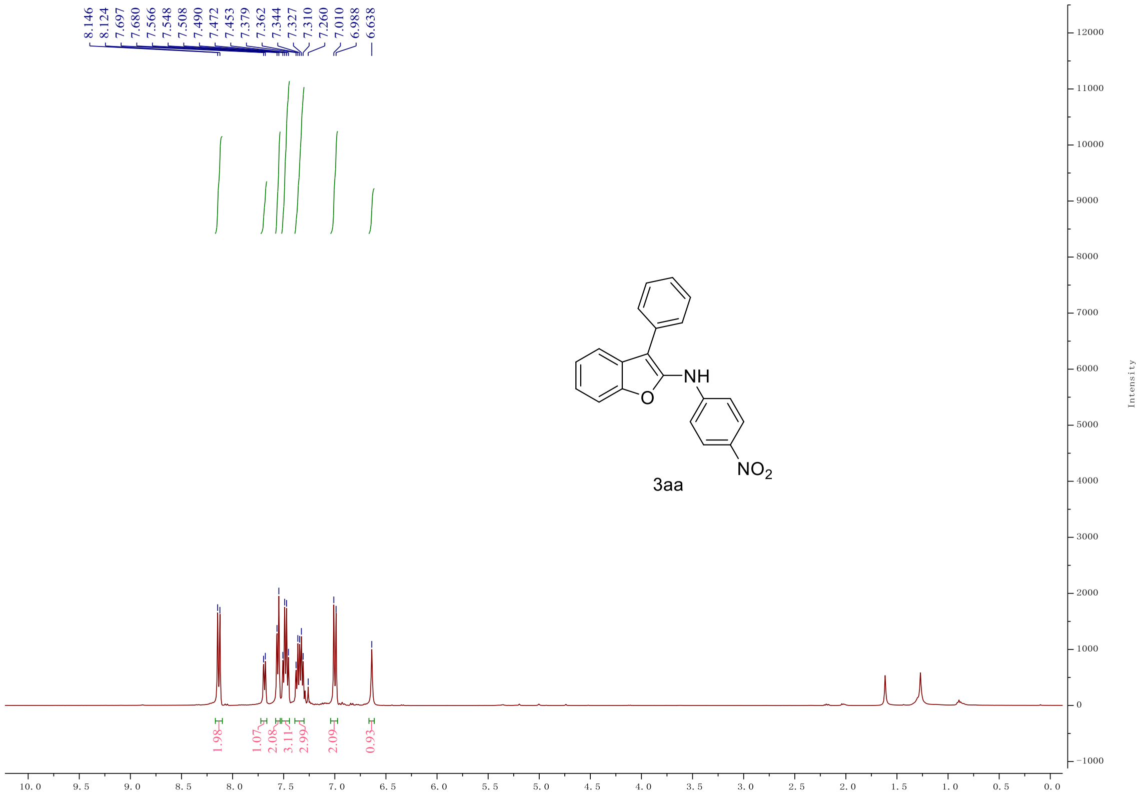

Figure S2. The ^{1}H NMR of compound **3aa**.

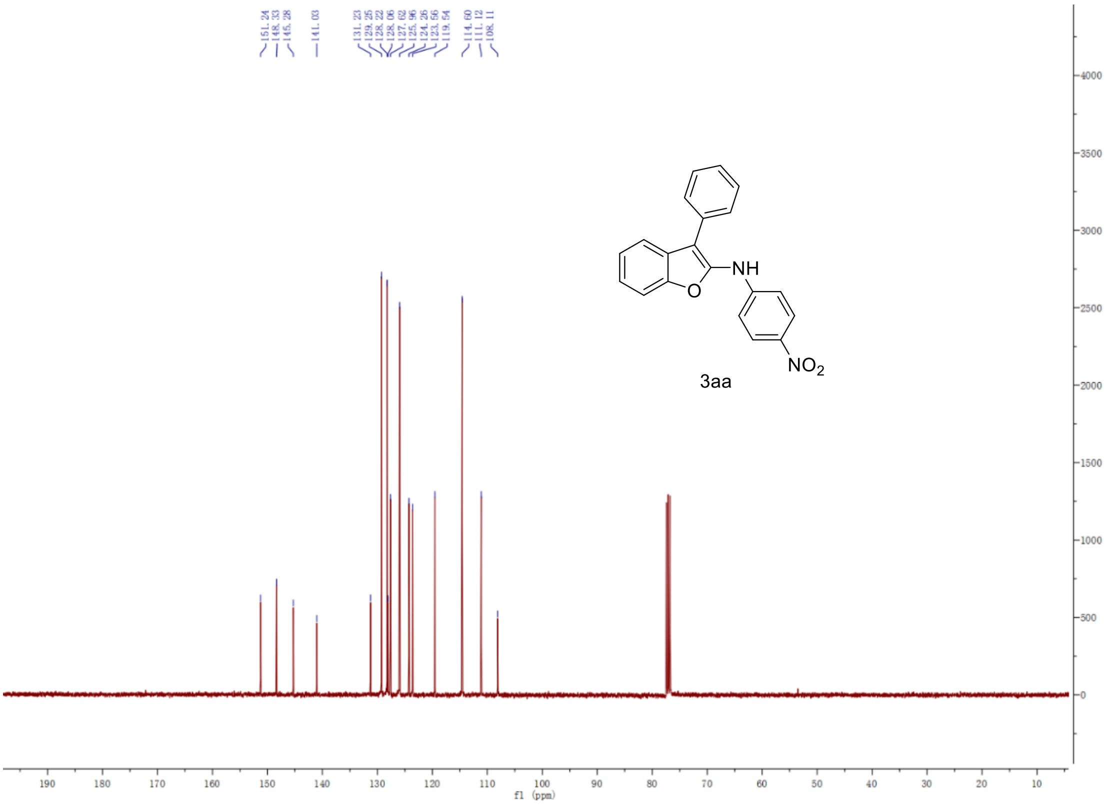

Figure S3. The ^{13}C NMR of compound **3aa**.

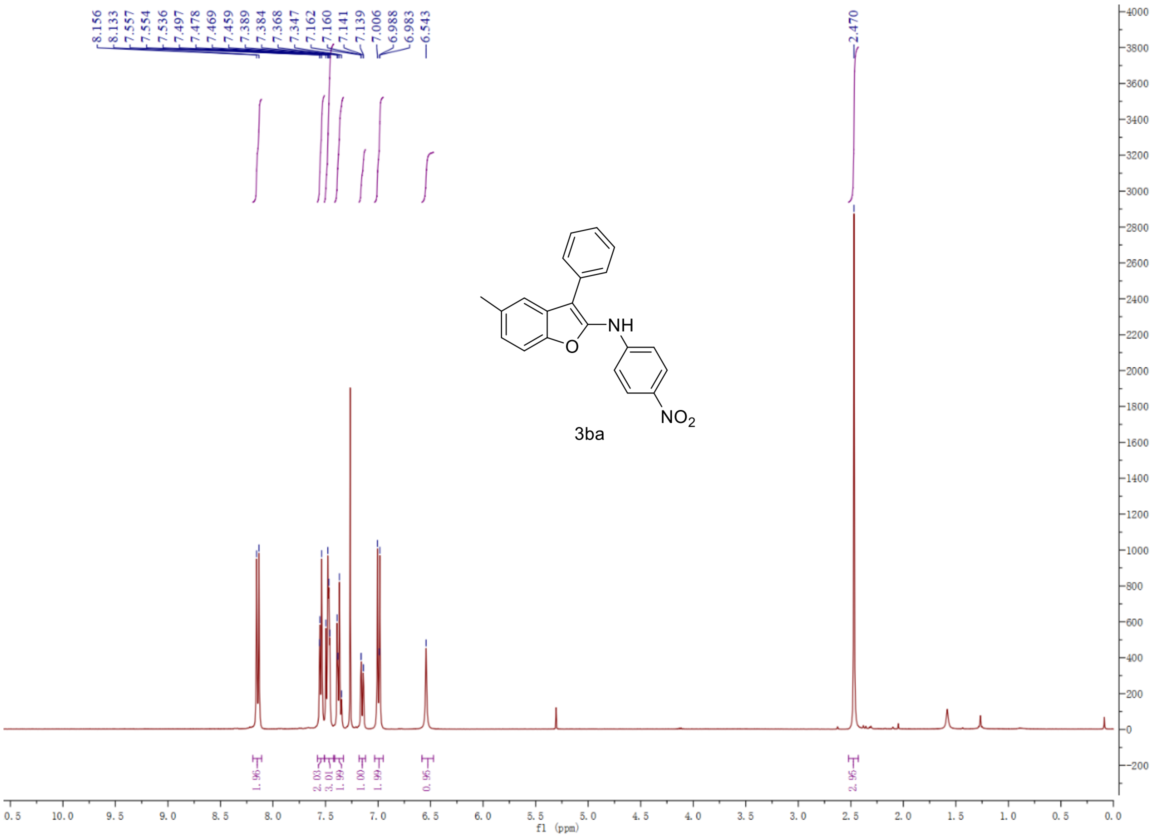

Figure S4. The ^{1}H NMR of compound **3ba**.

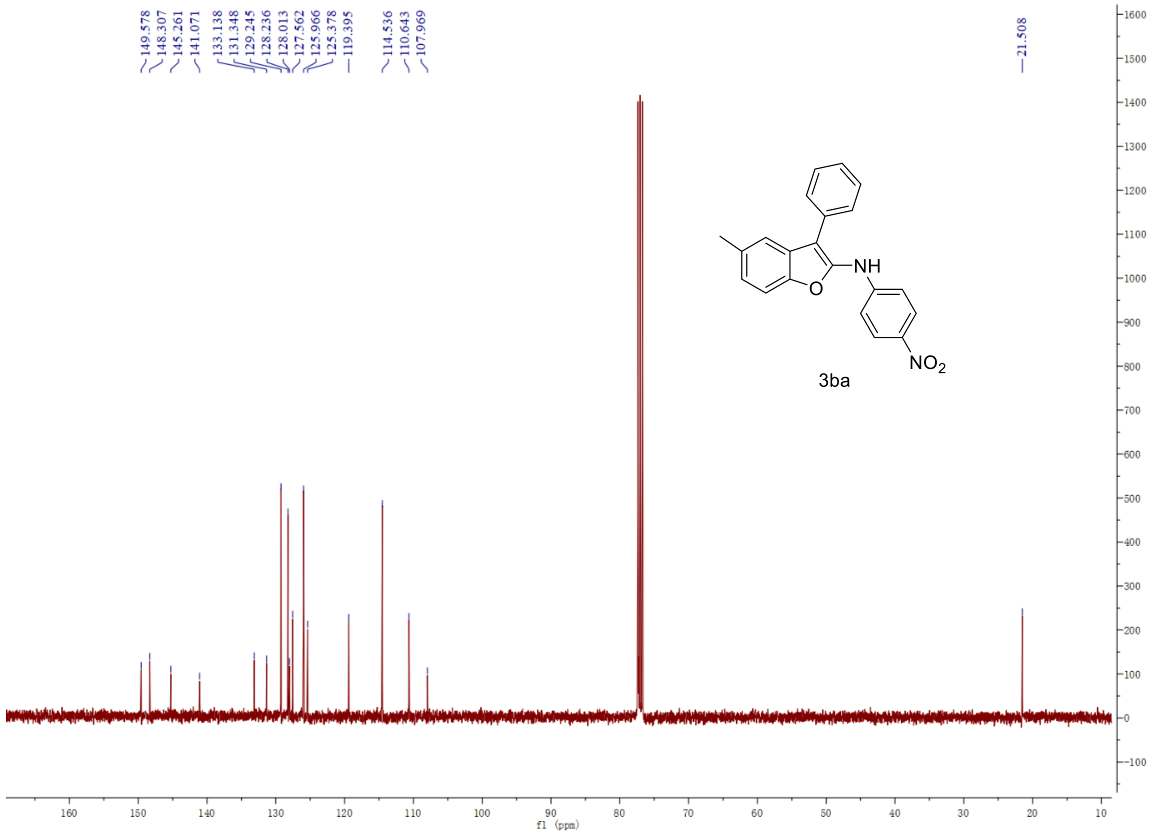

Figure S5. The ^{13}C NMR of compound **3ba**.

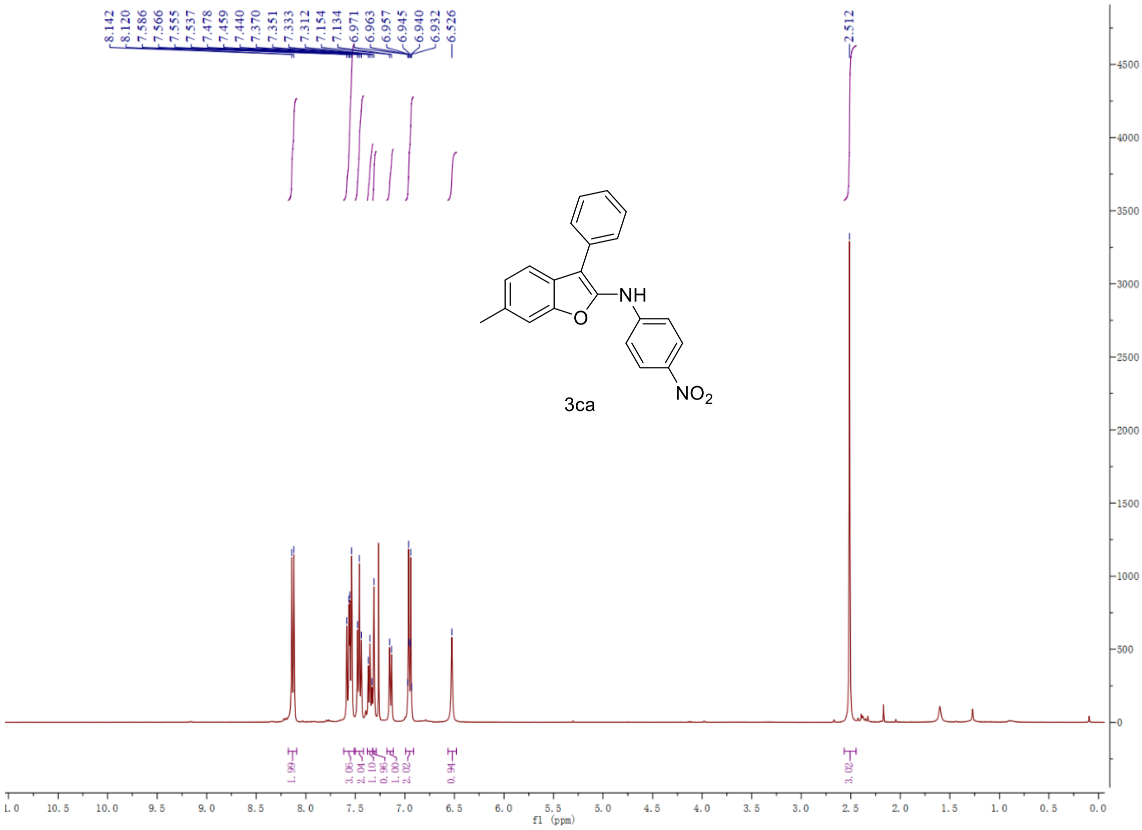

Figure S6. The ^{1}H NMR of compound **3ca**.

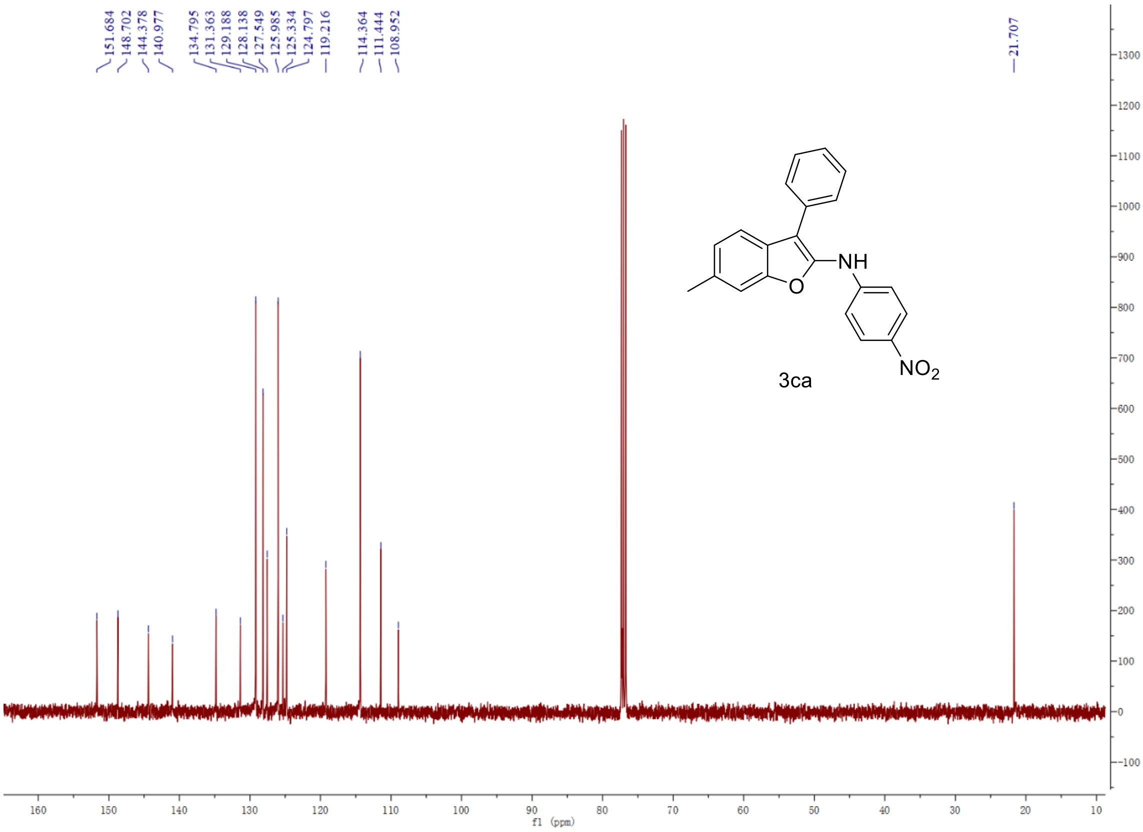

Figure S7. The ^{13}C NMR of compound **3ca**.

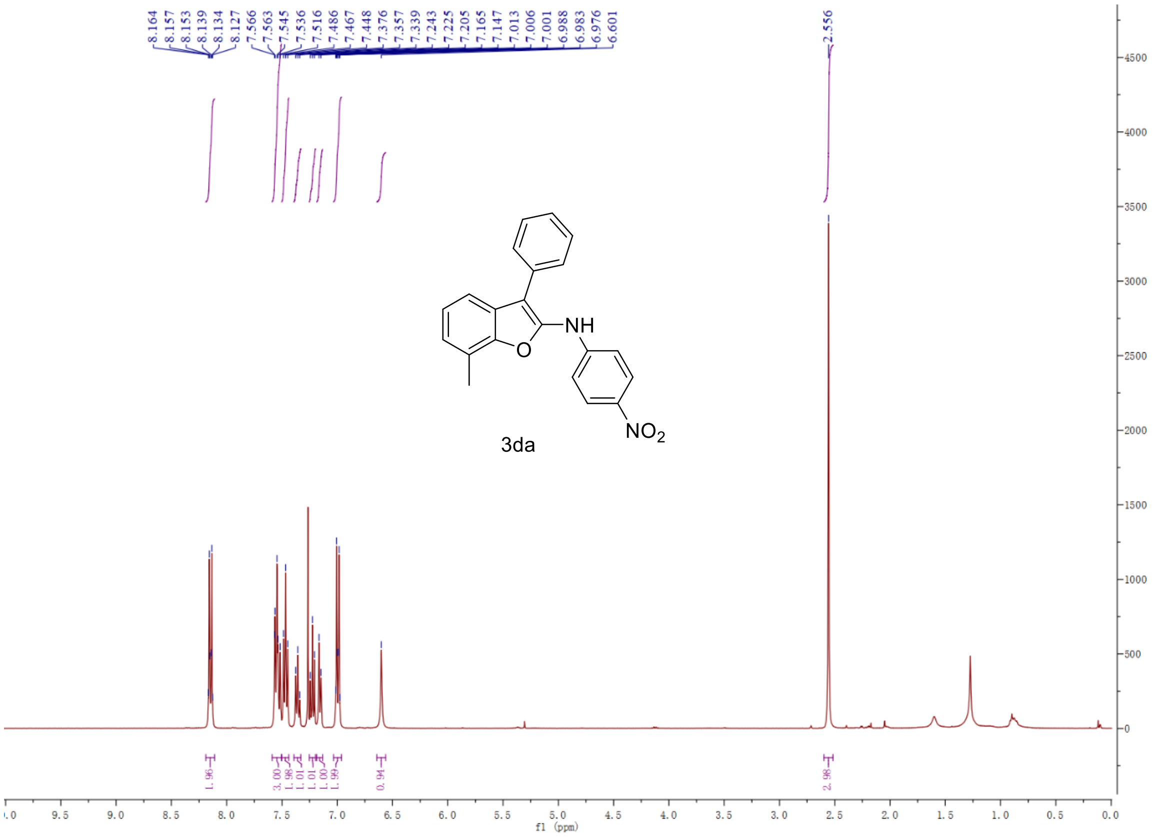

Figure S8. The ^{1}H NMR of compound **3da**.

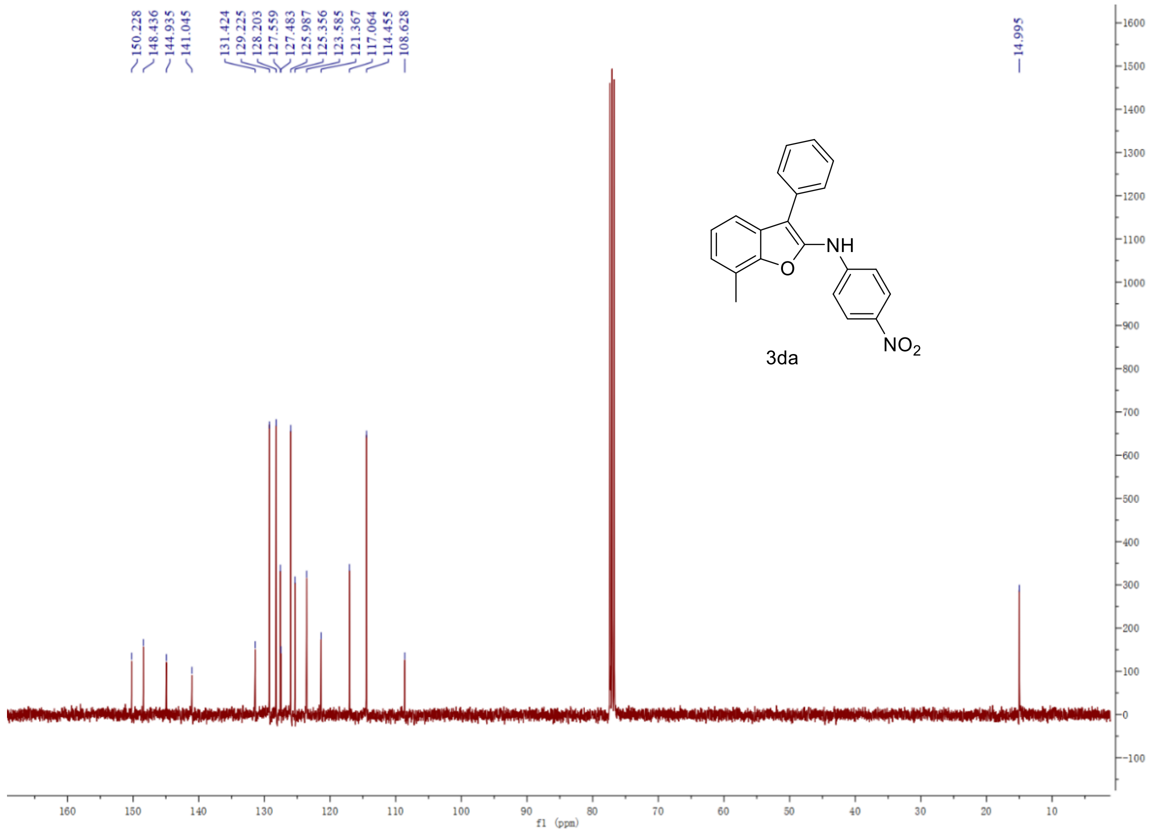

Figure S9. The ^{13}C NMR of compound **3da**.

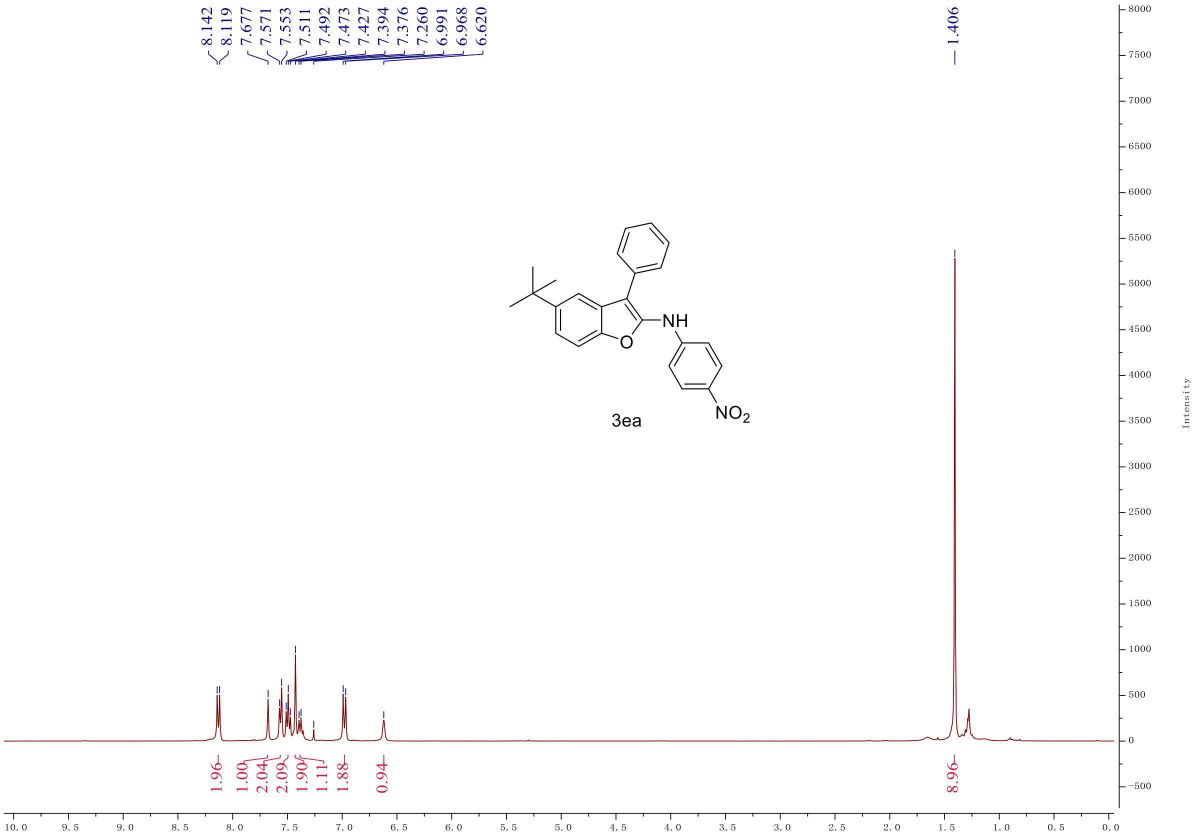

Figure S10. The ^{1}H NMR of compound **3ea**.

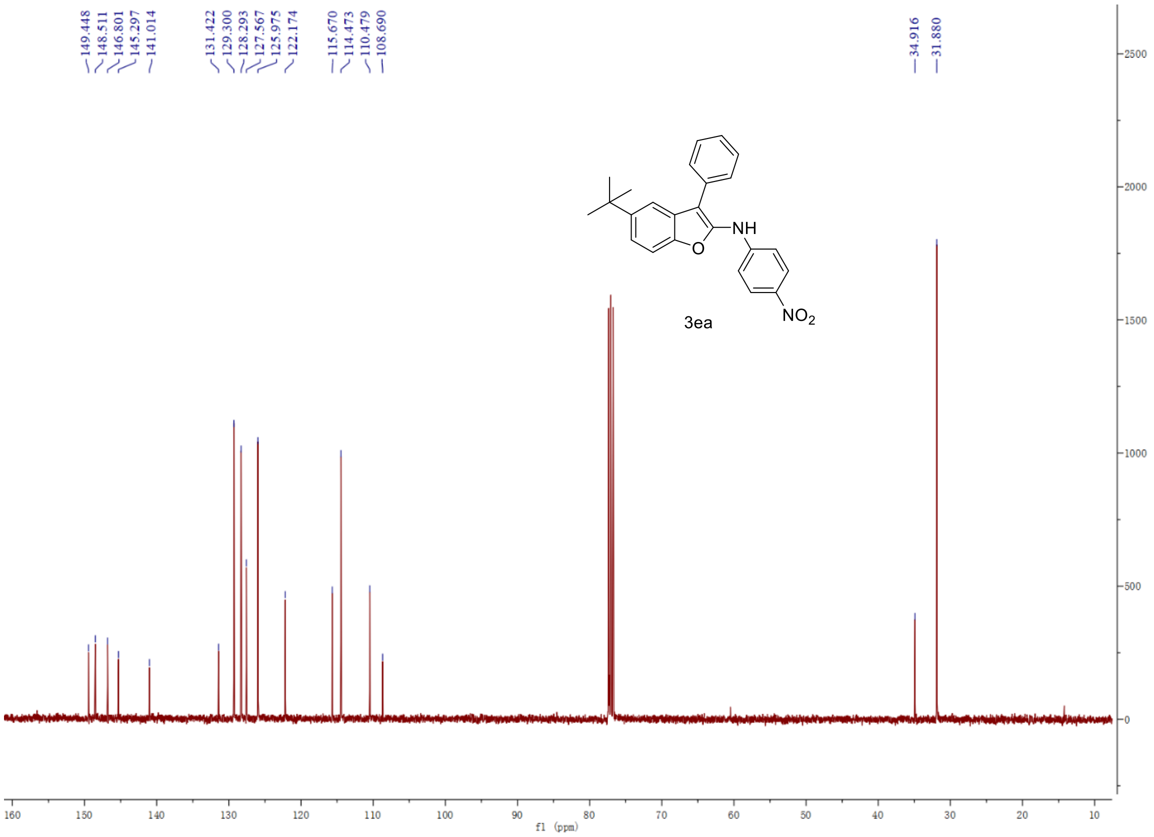

Figure S11. The ^{13}C NMR of compound **3ea**.

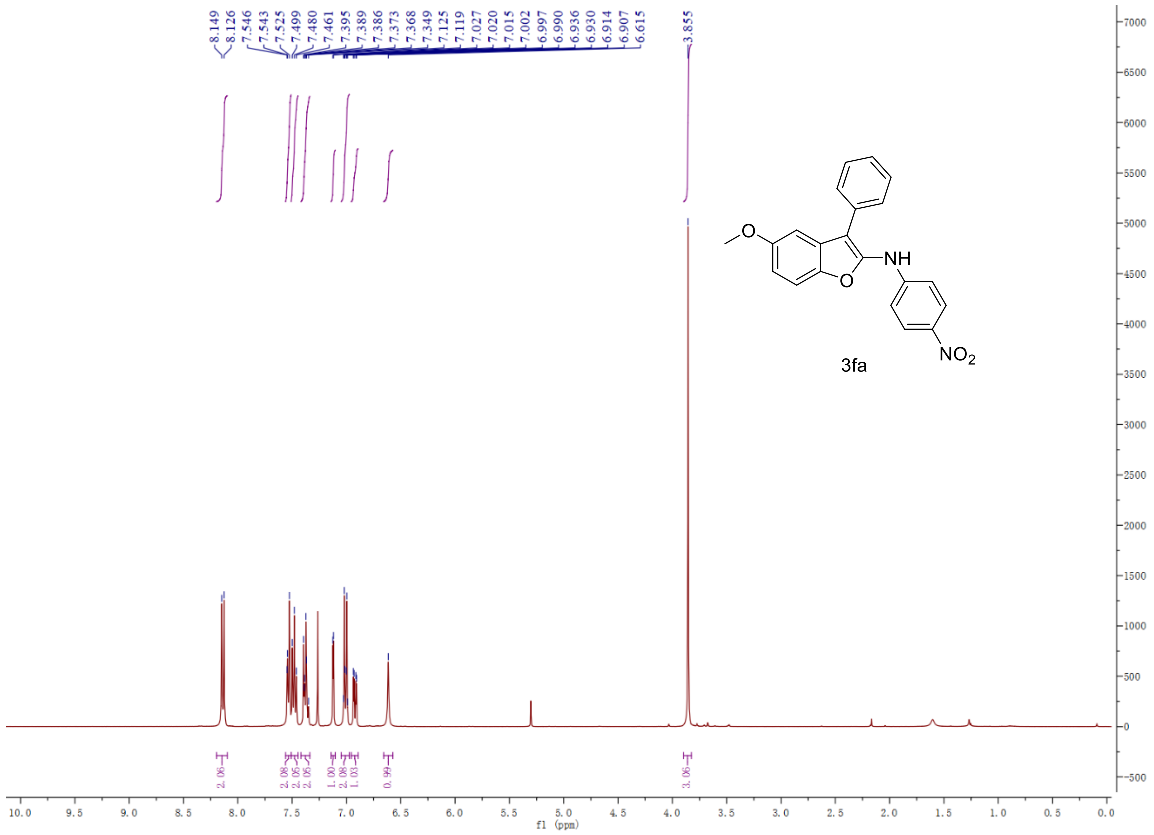

Figure S12. The ^{1}H NMR of compound **3fa**.

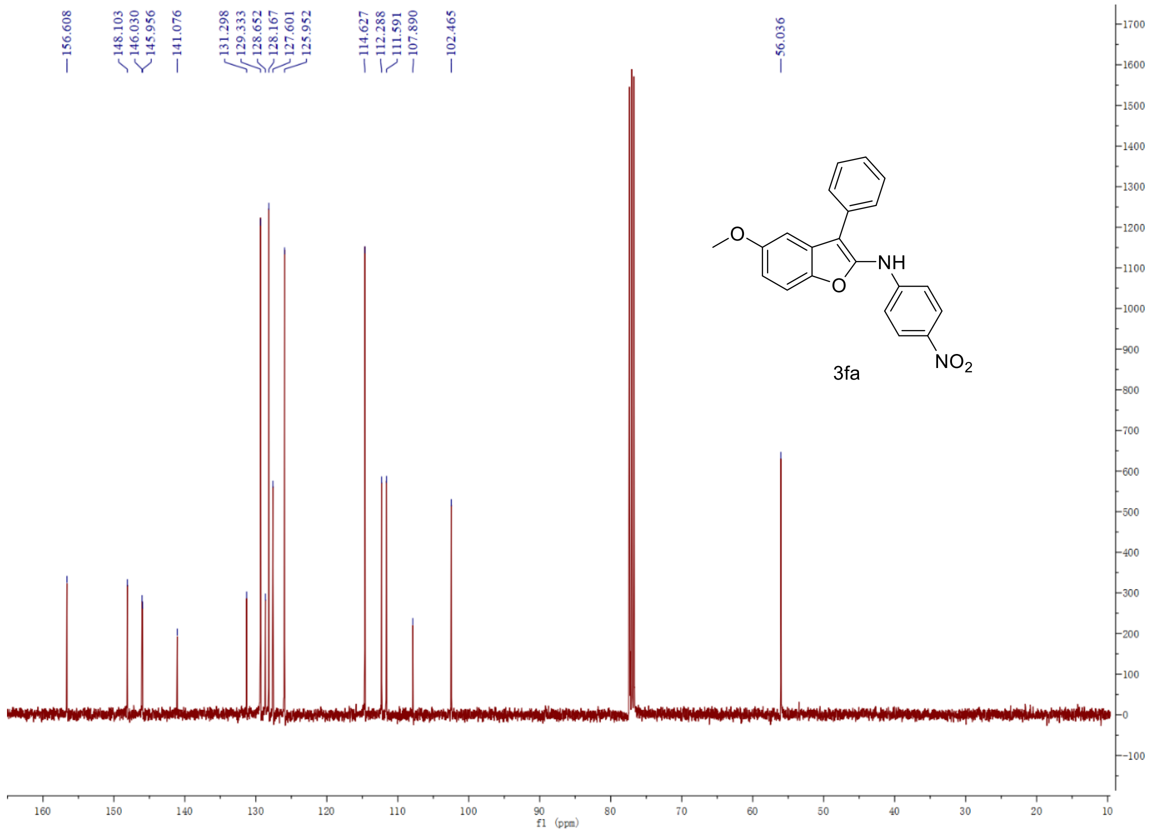

Figure S13. The ^{13}C NMR of compound **3fa**.

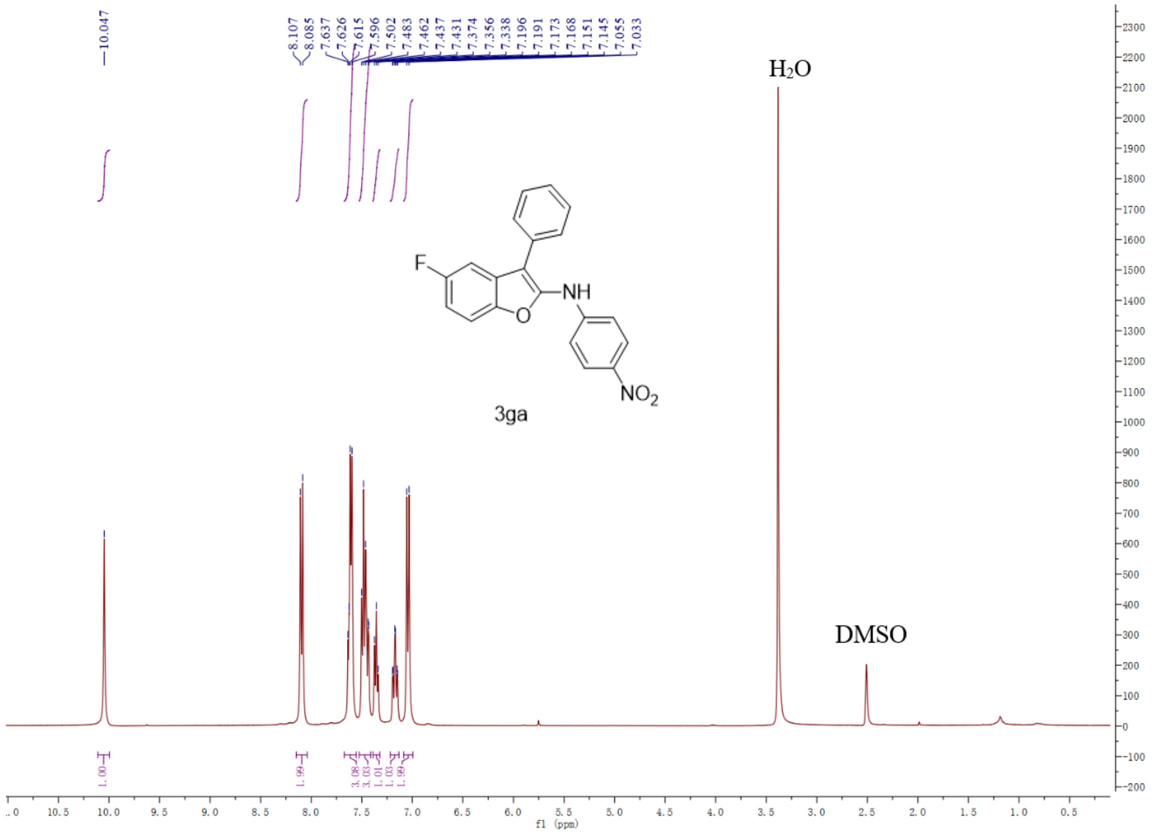

Figure S14. The ^{1}H NMR of compound **3ga**.

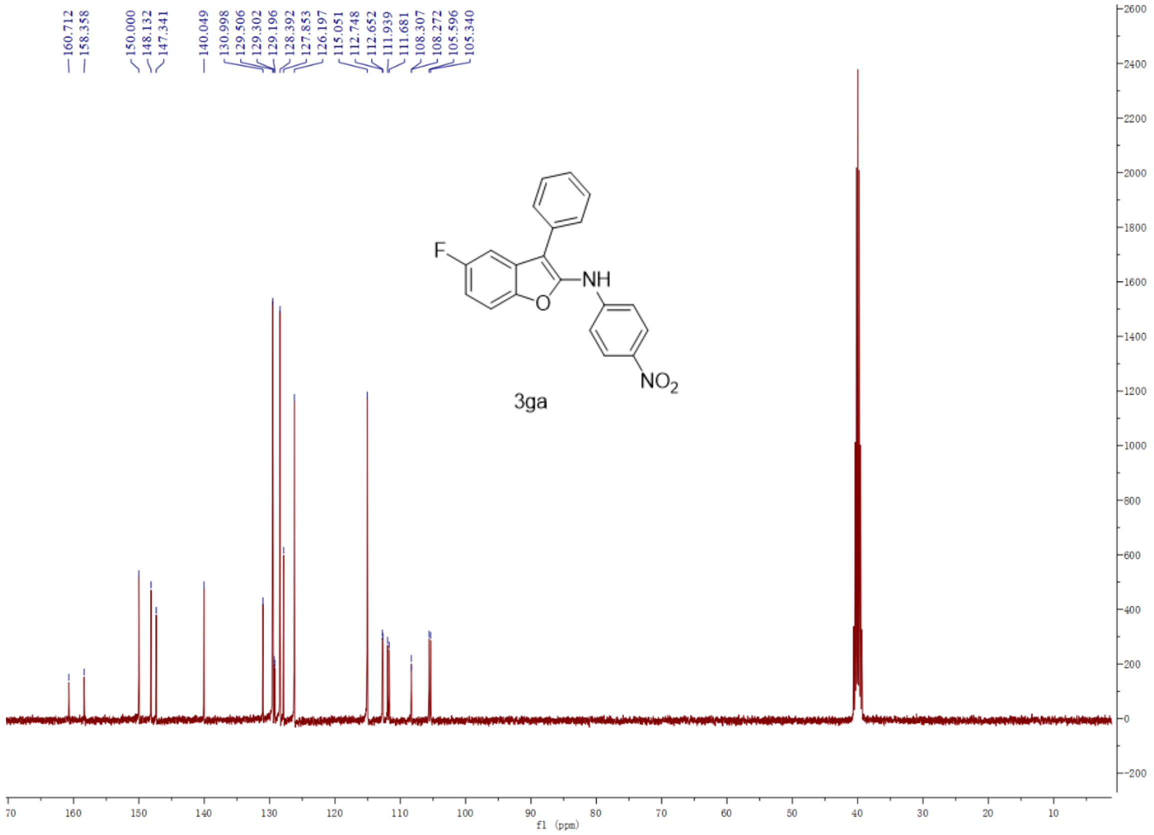

Figure S15. The ^{13}C NMR of compound **3ga**.

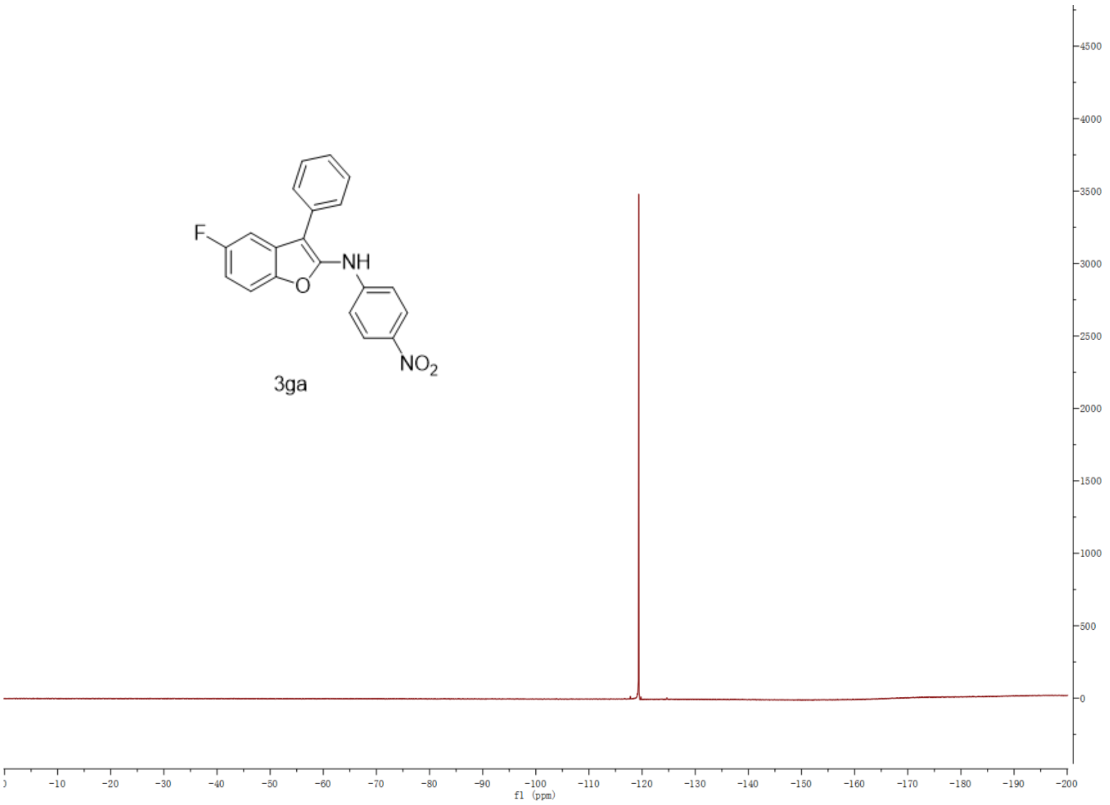

Figure S16. The ^{19}F NMR of compound **3ga**.

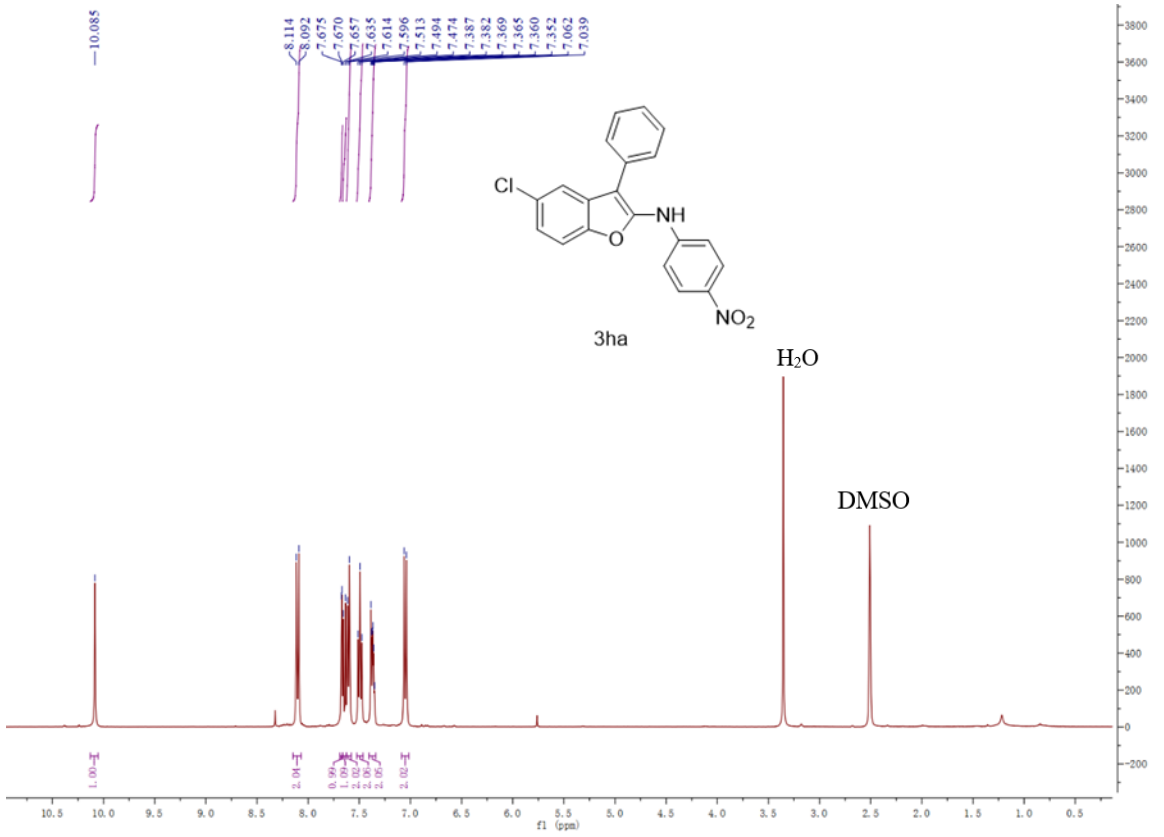

Figure S17. The ^{1}H NMR of compound **3ha**.

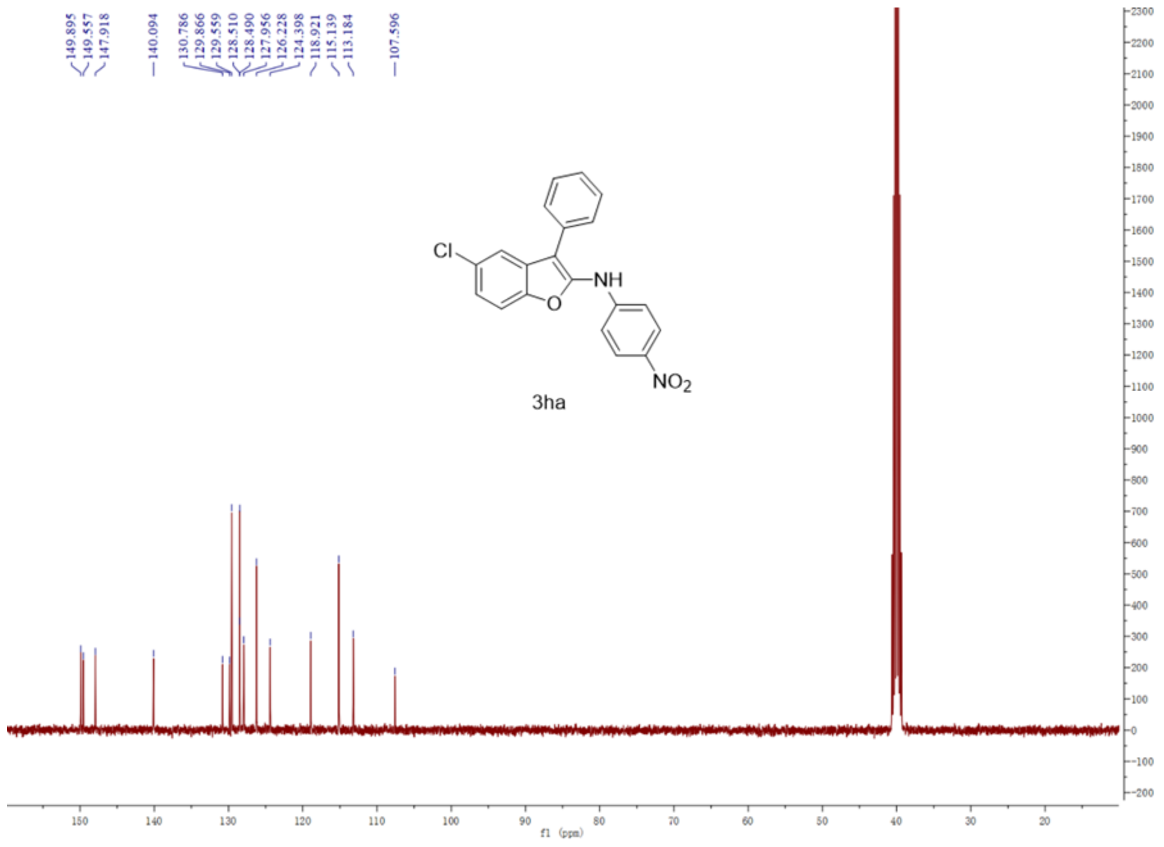

Figure S18. The ^{13}C NMR of compound **3ha**.

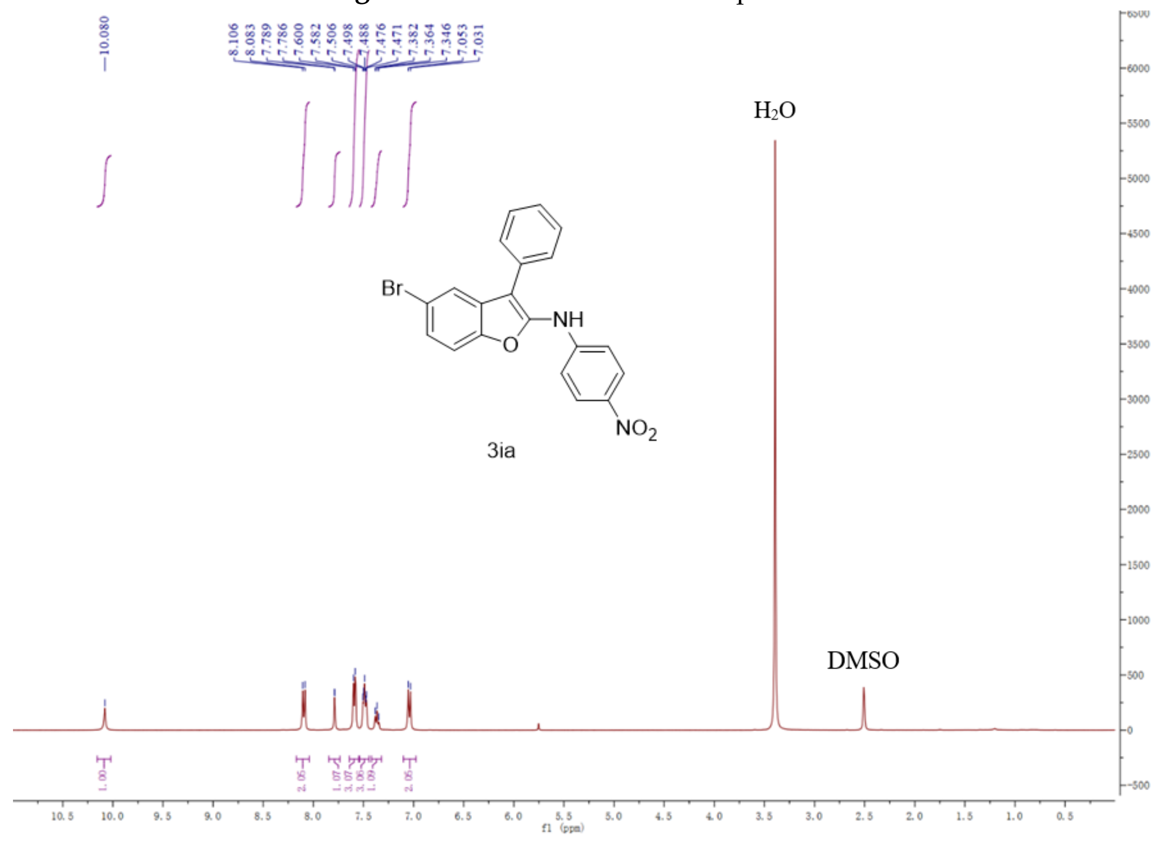

Figure S19. The ^{1}H NMR of compound **3ia**.

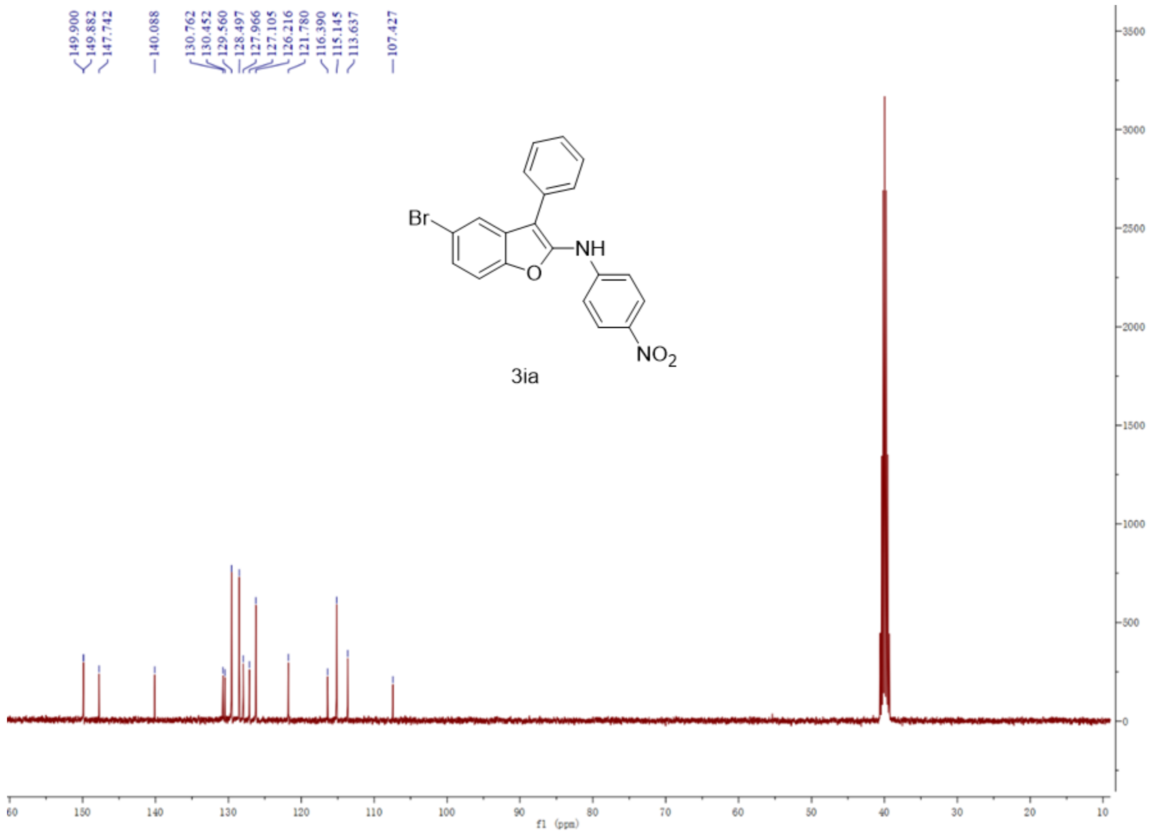

Figure S20. The ^{13}C NMR of compound **3ia**.

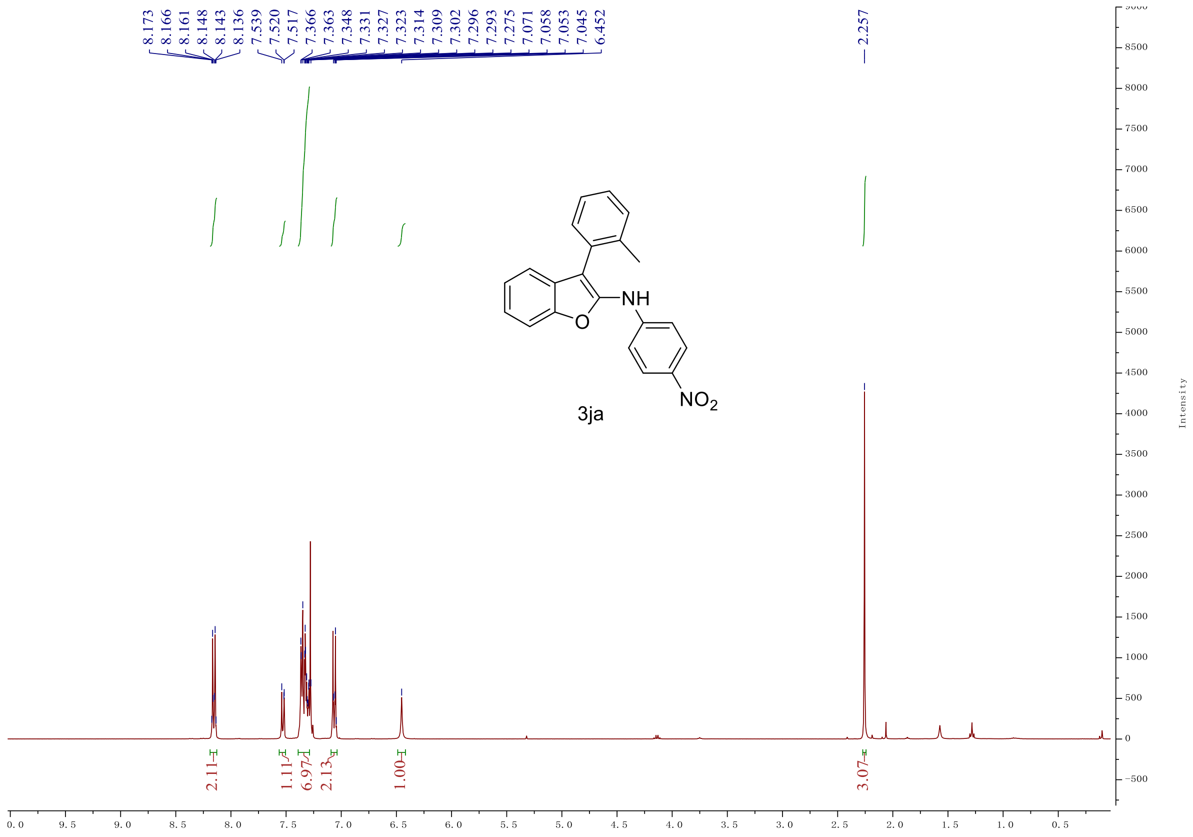

Figure S21. The ^{1}H NMR of compound **3ja**.

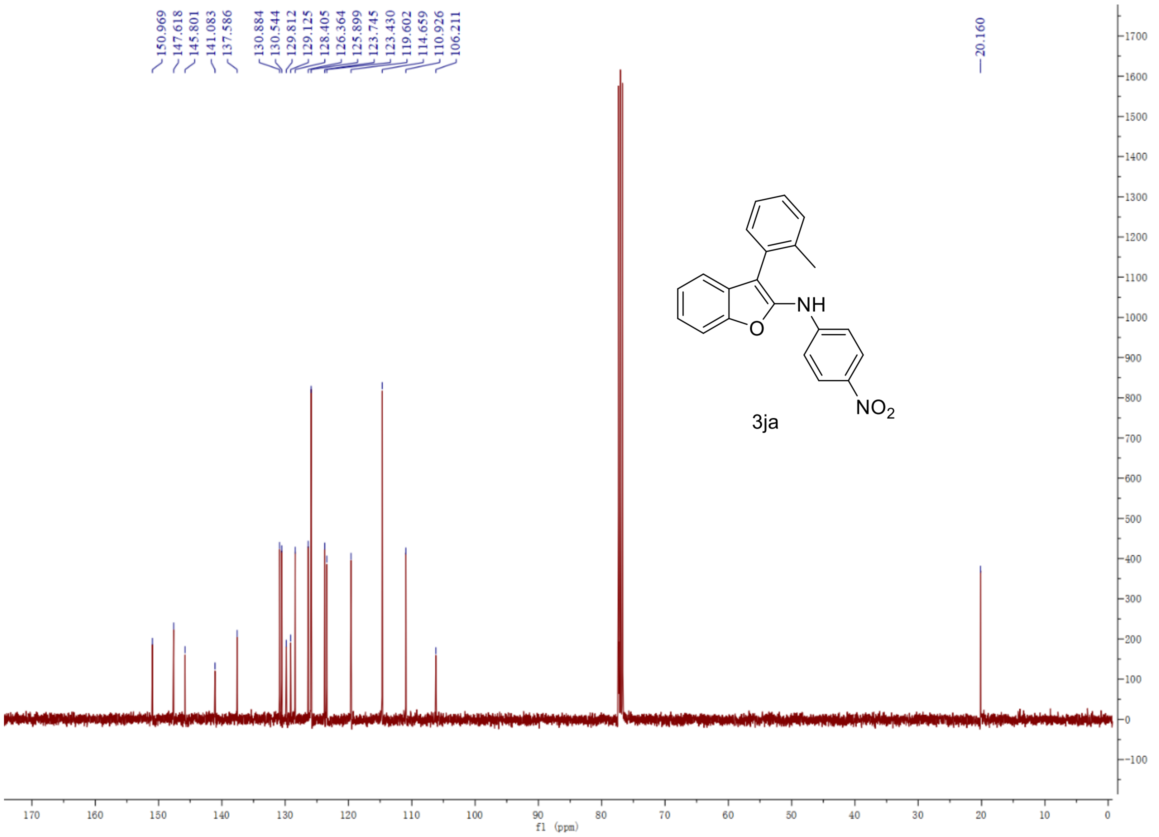

Figure S22. The ^{13}C NMR of compound **3ja**.

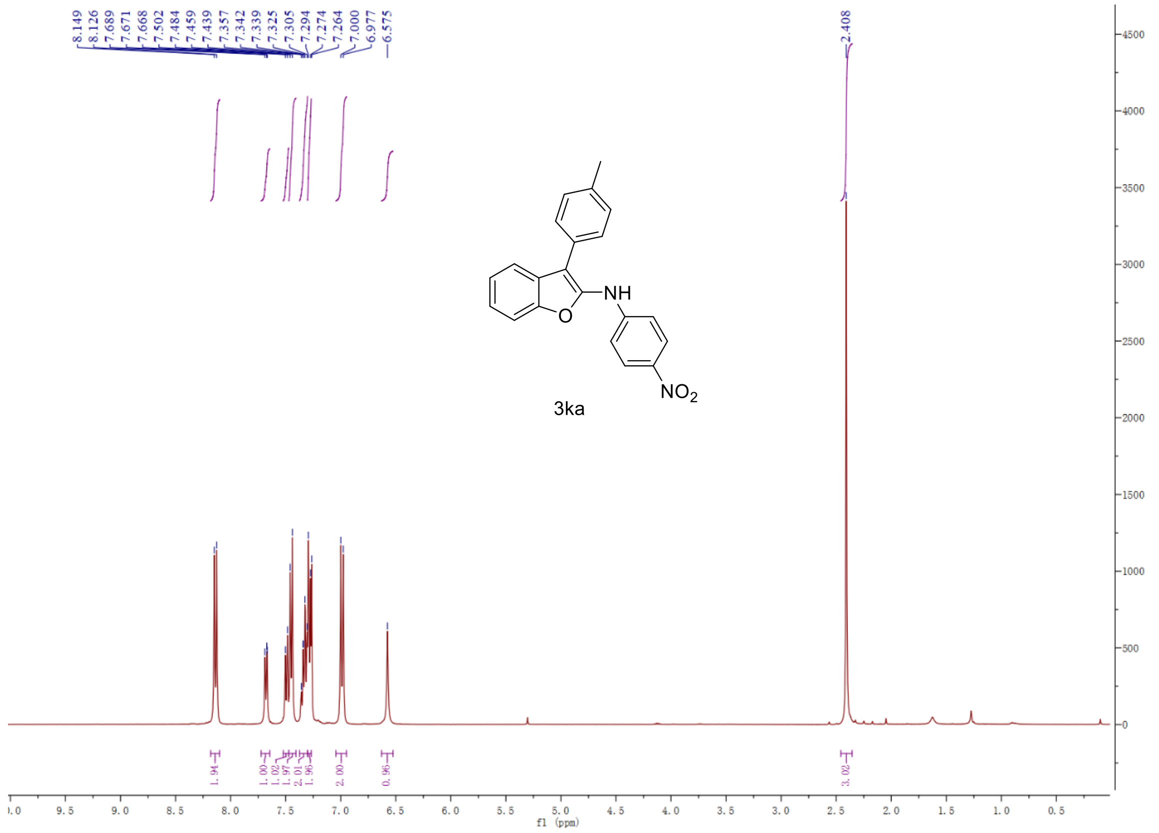

Figure S23. The ^{1}H NMR of compound **3ka**.

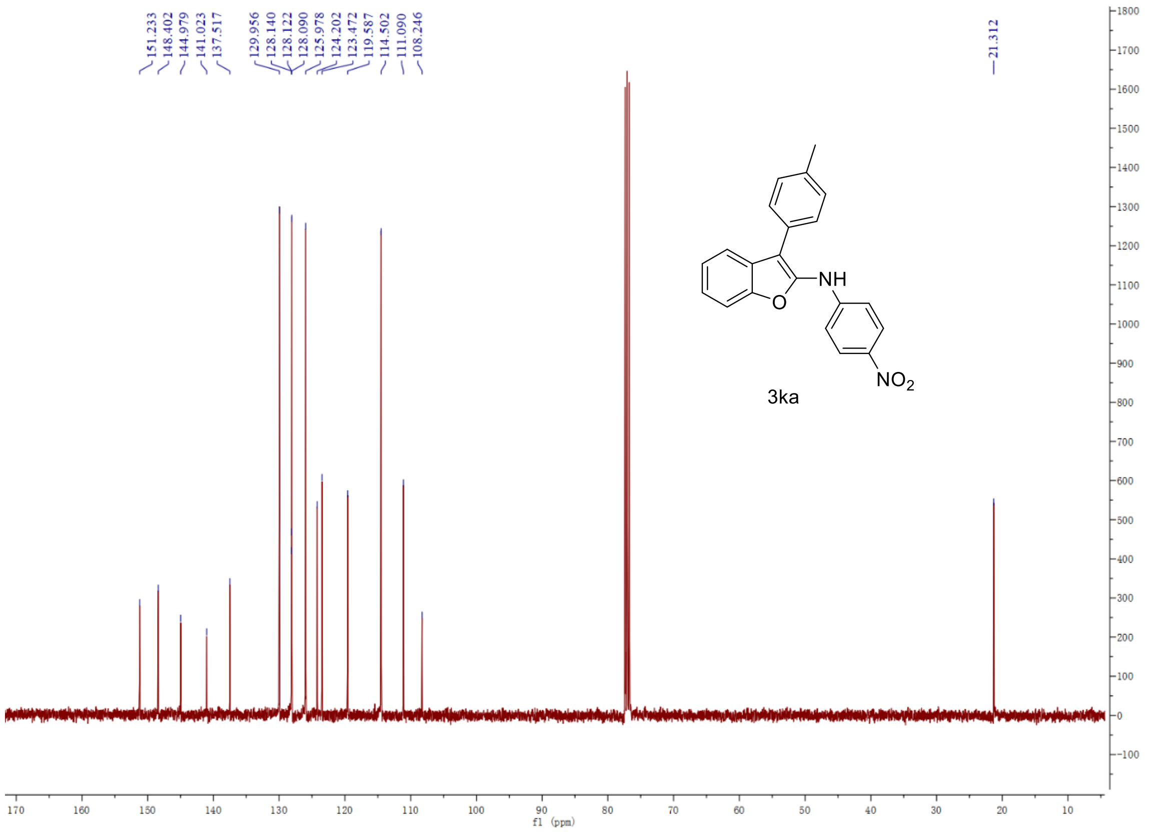

Figure S24. The ^{13}C NMR of compound **3ka**.

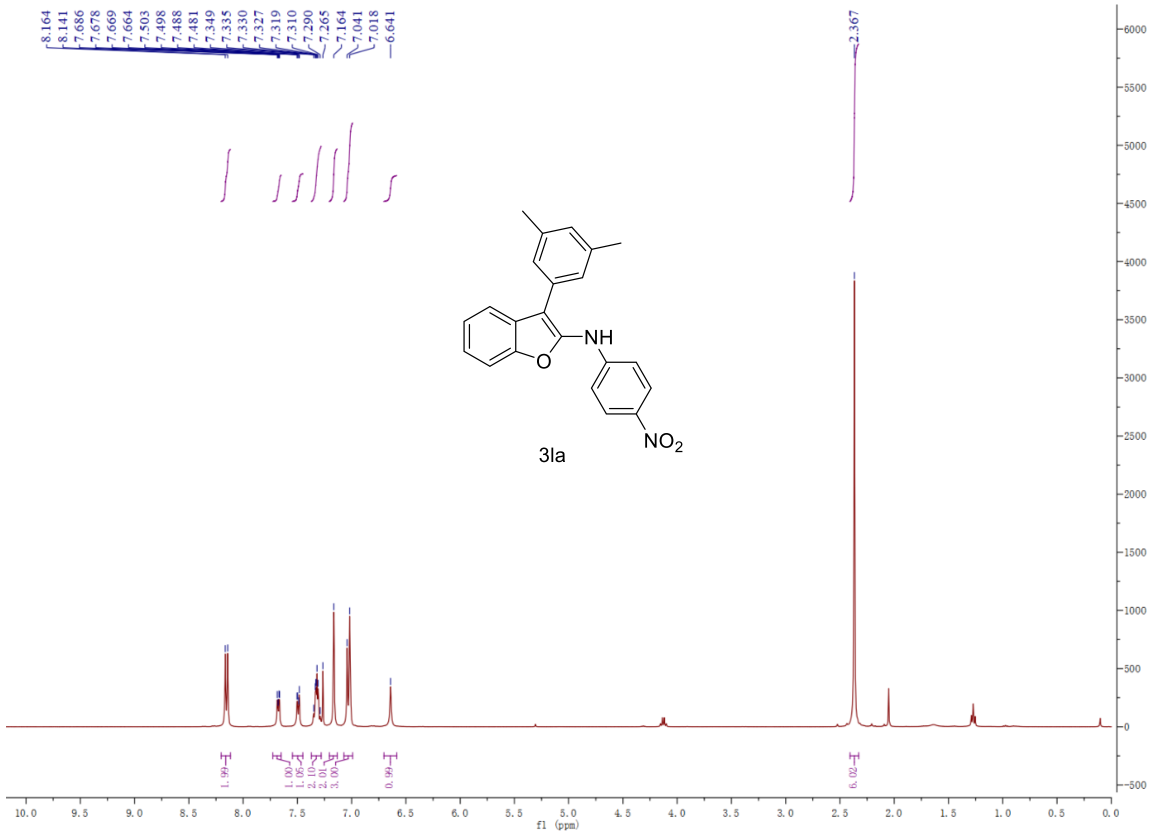

Figure S25. The ^{1}H NMR of compound **3la**.

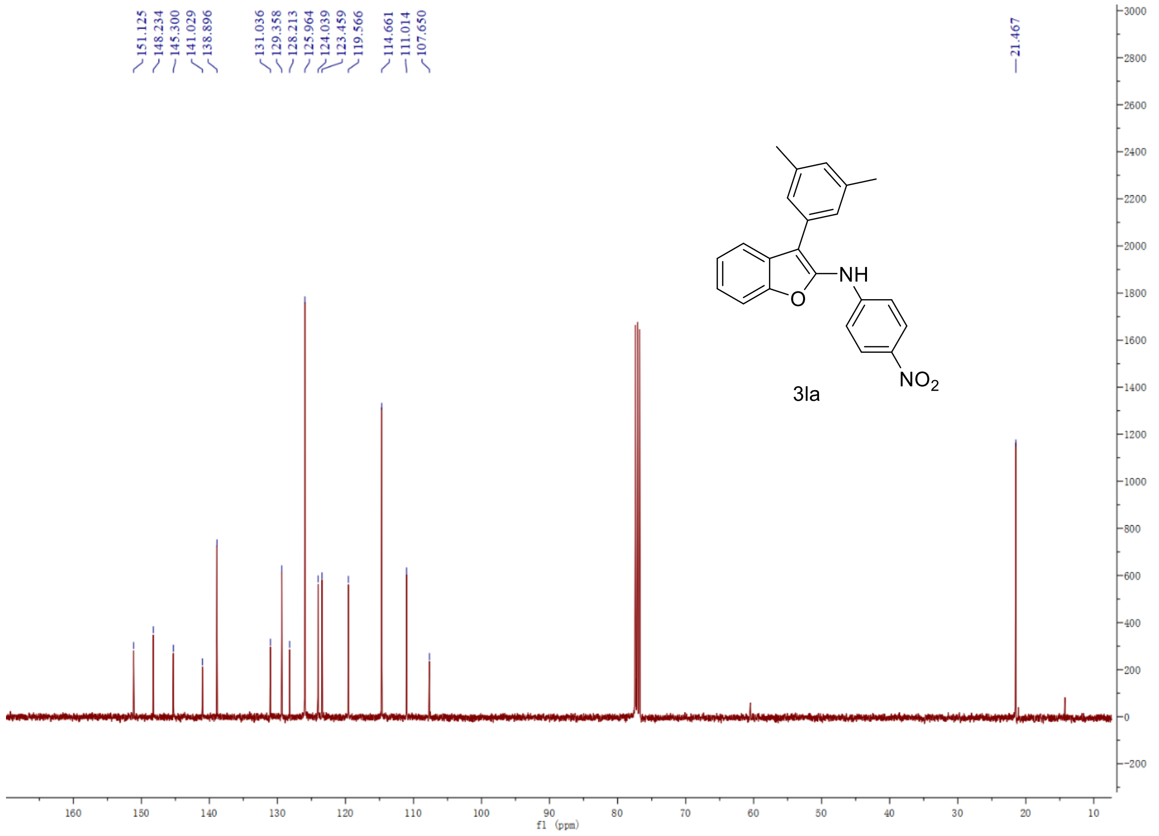

Figure S26. The ^{13}C NMR of compound **3la.**

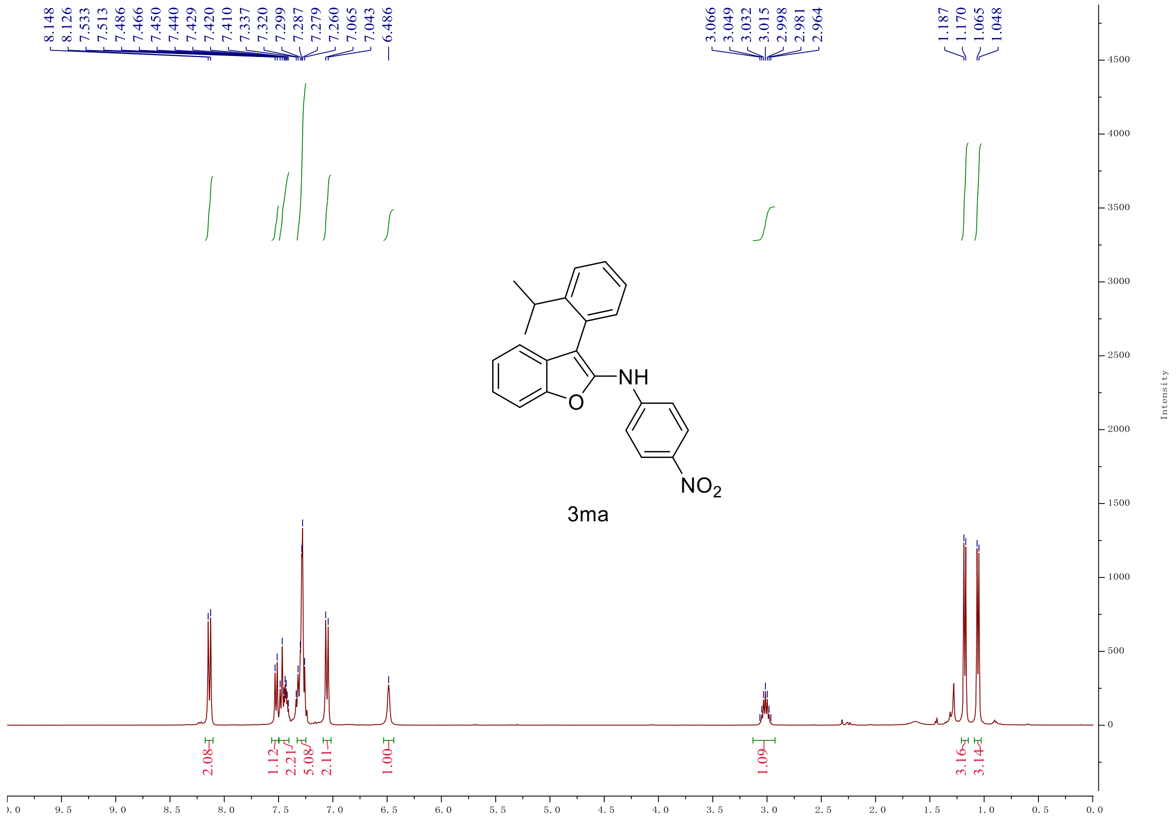

Figure S27. The ^{1}H NMR of compound **3ma**.

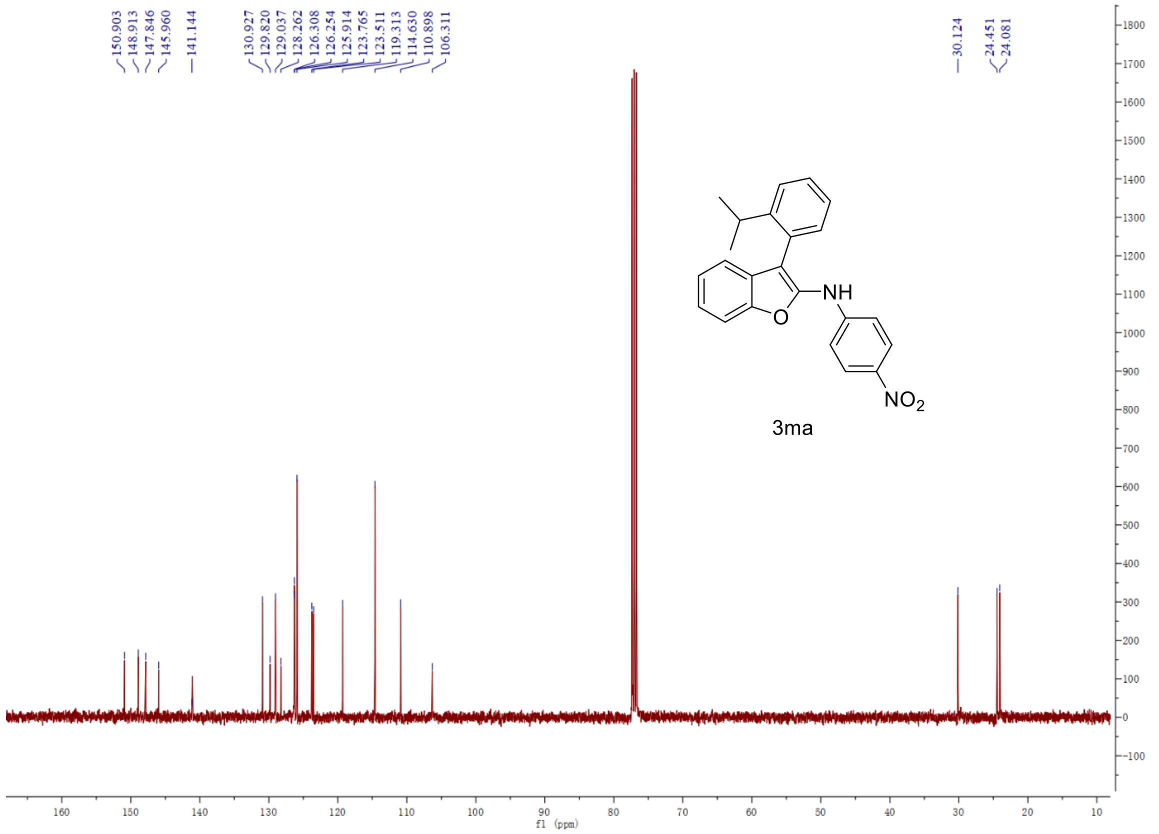

Figure S28. The ^{13}C NMR of compound **3ma**.

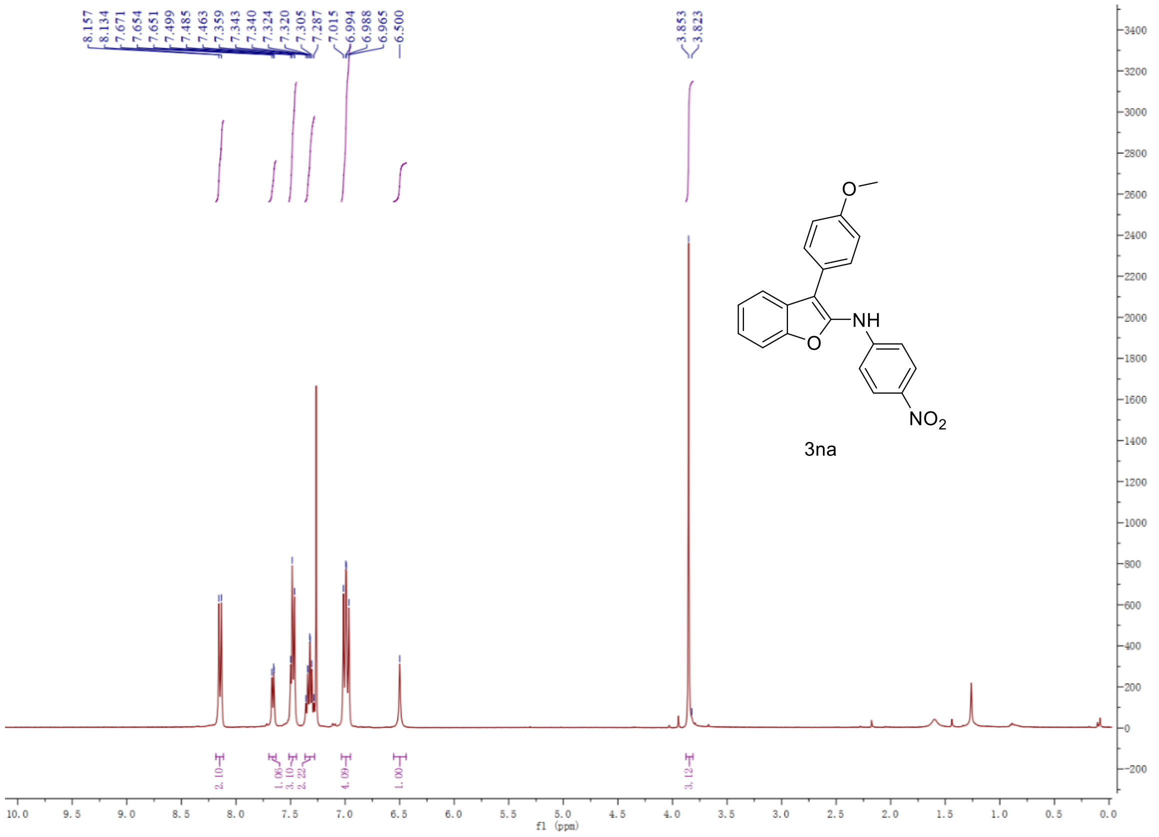

Figure S29. The ^{1}H NMR of compound **3na**.

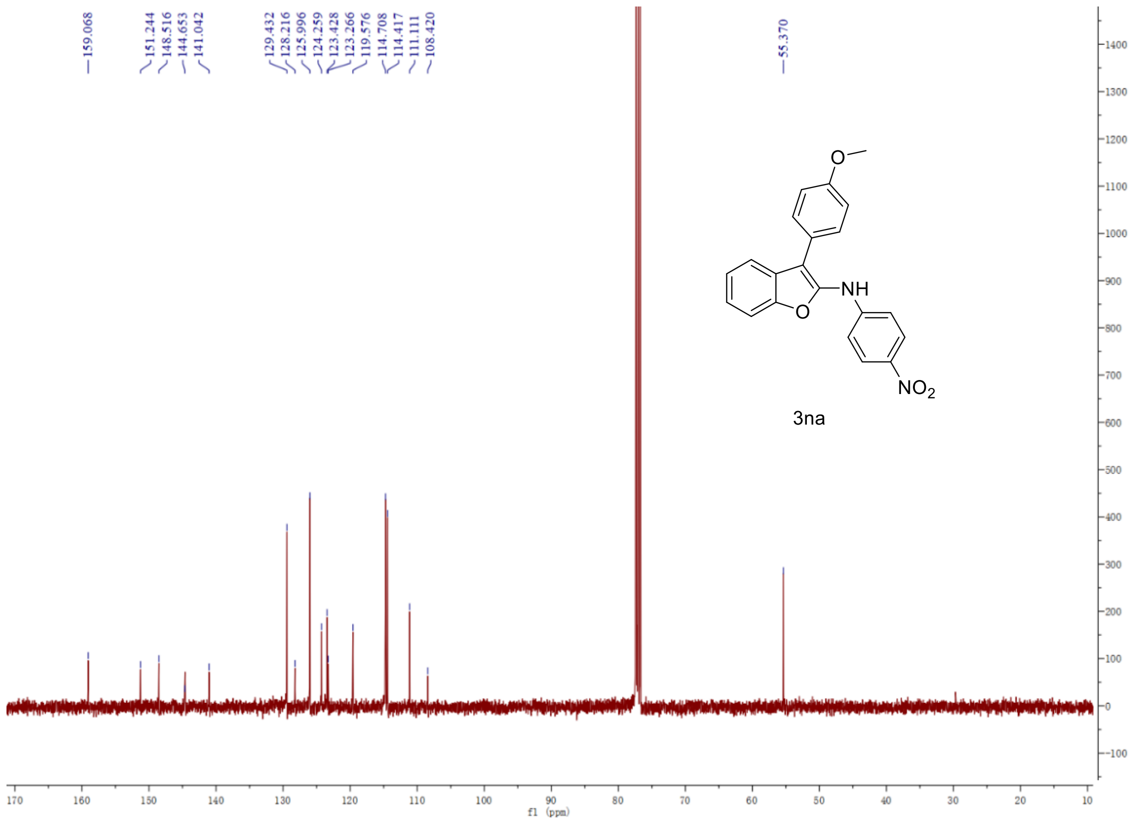

Figure S30. The ^{13}C NMR of compound **3na**.

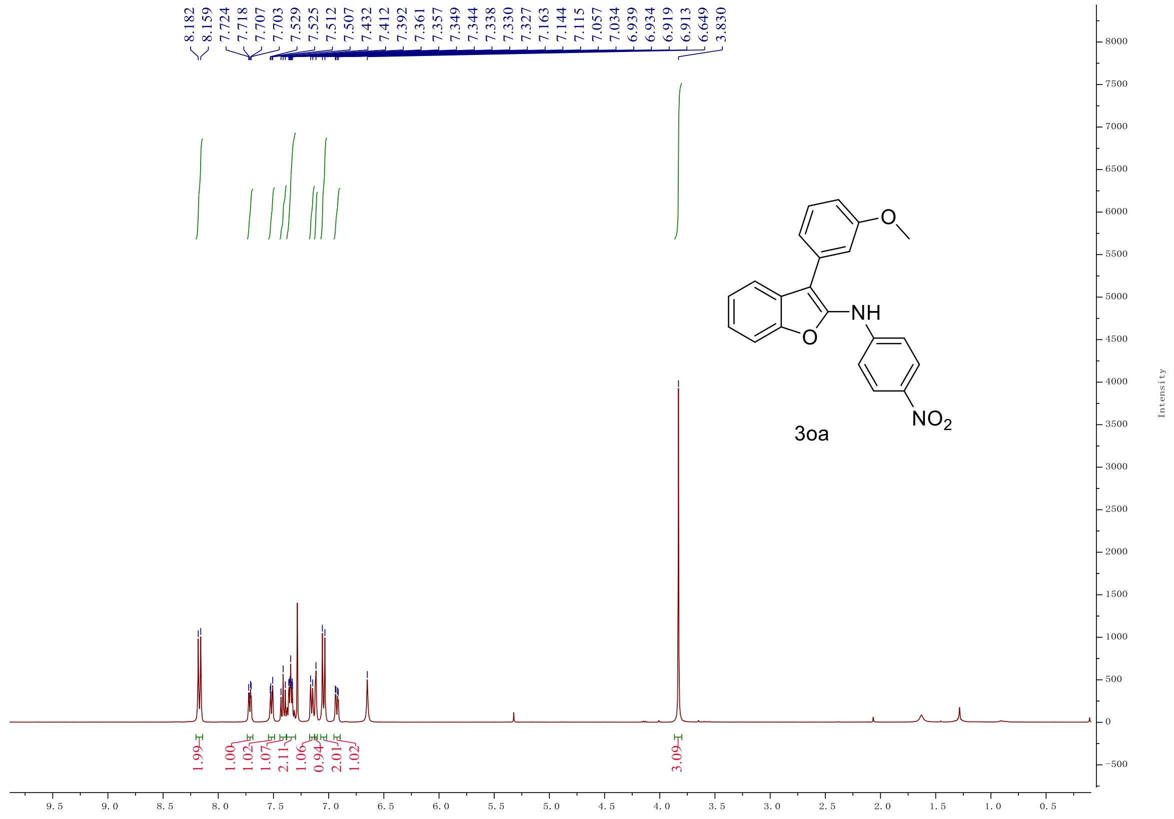

Figure S31. The ^{1}H NMR of compound **3oa**.

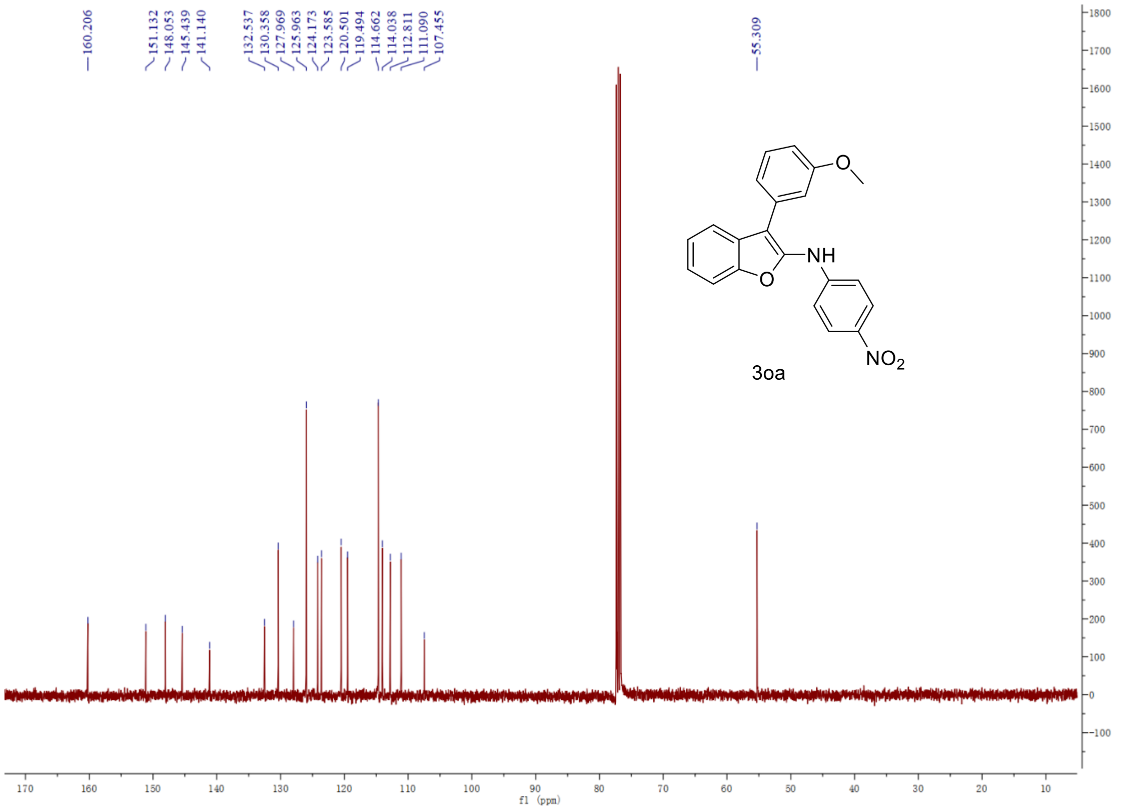

Figure S32. The ^{13}C NMR of compound **3oa**.

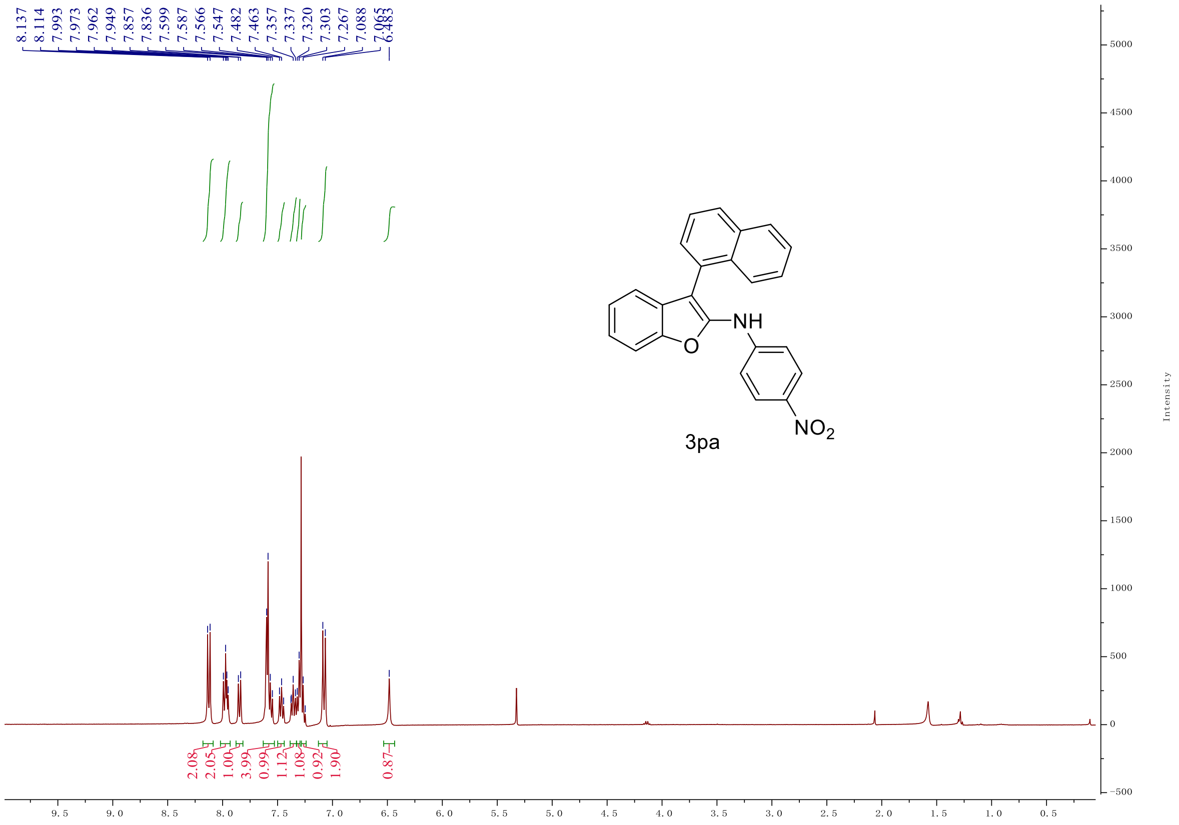

Figure S33. The ^{1}H NMR of compound **3pa**.

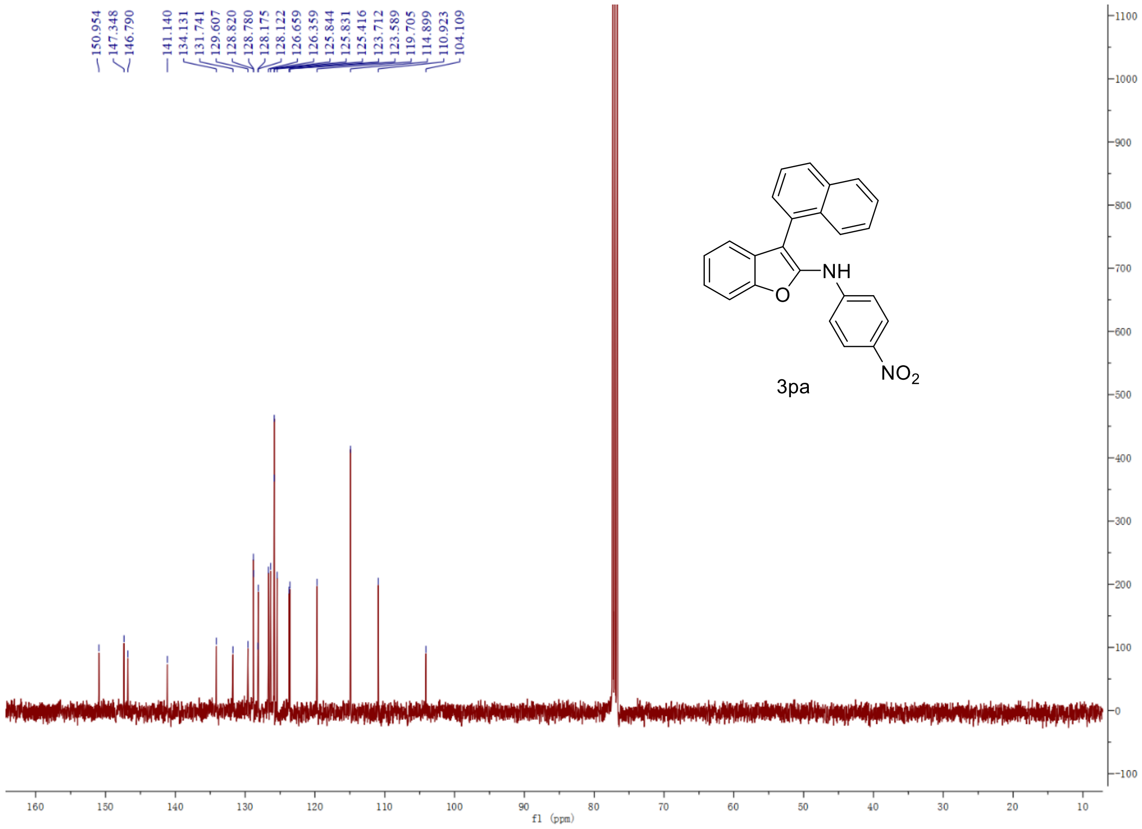

Figure S34. The ^{13}C NMR of compound **3pa**.

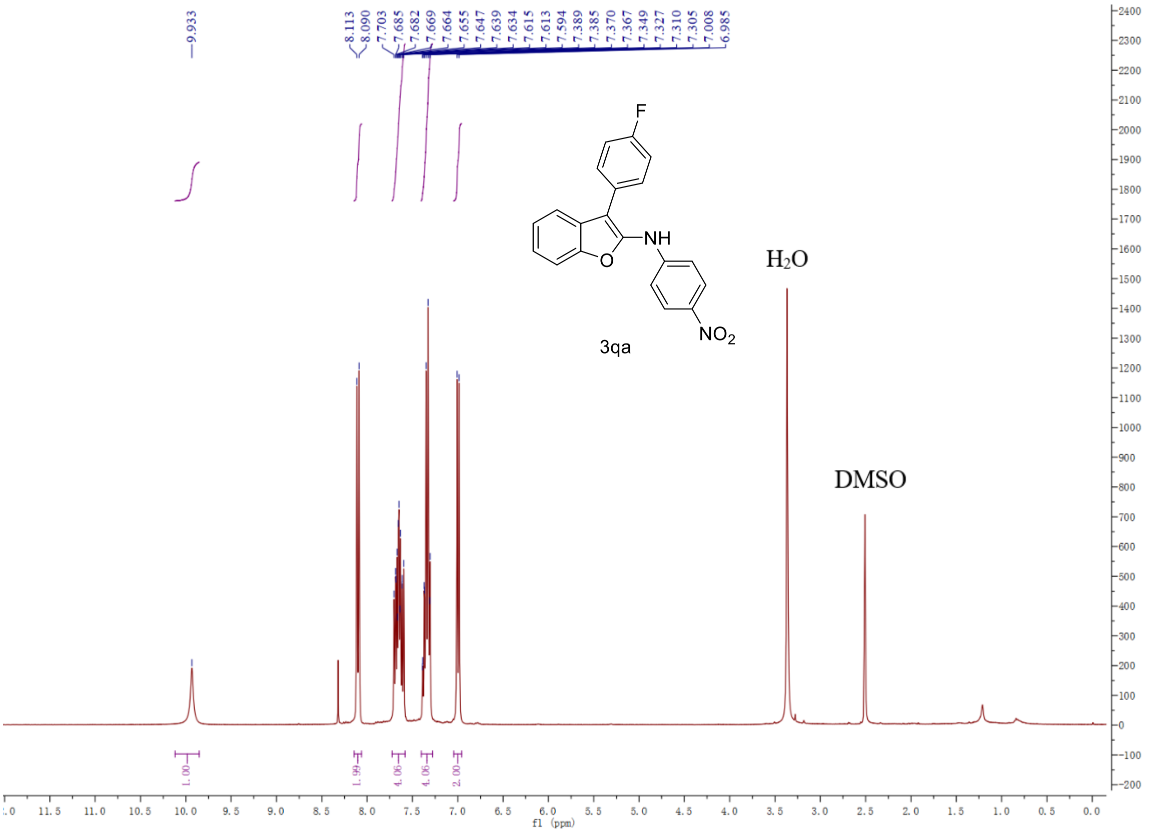

Figure S35. The ^{1}H NMR of compound **3qa**.

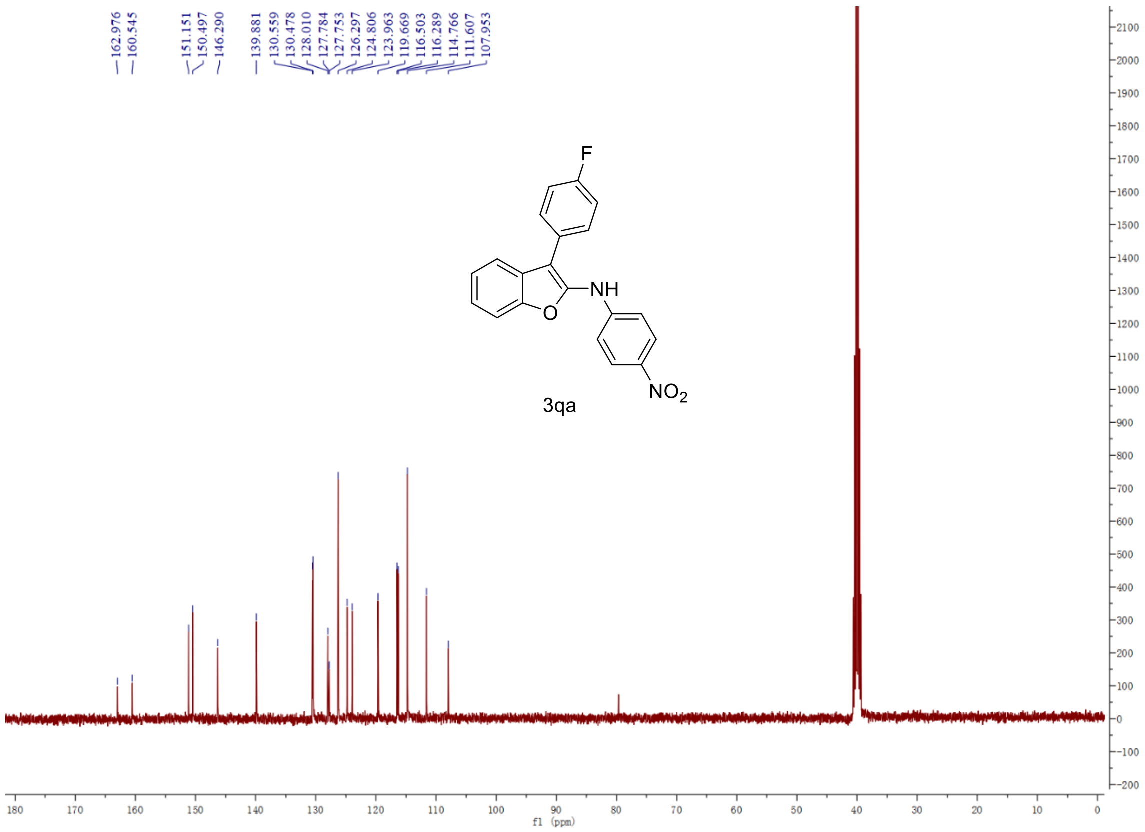

Figure S36. The ^{13}C NMR of compound **3qa**.

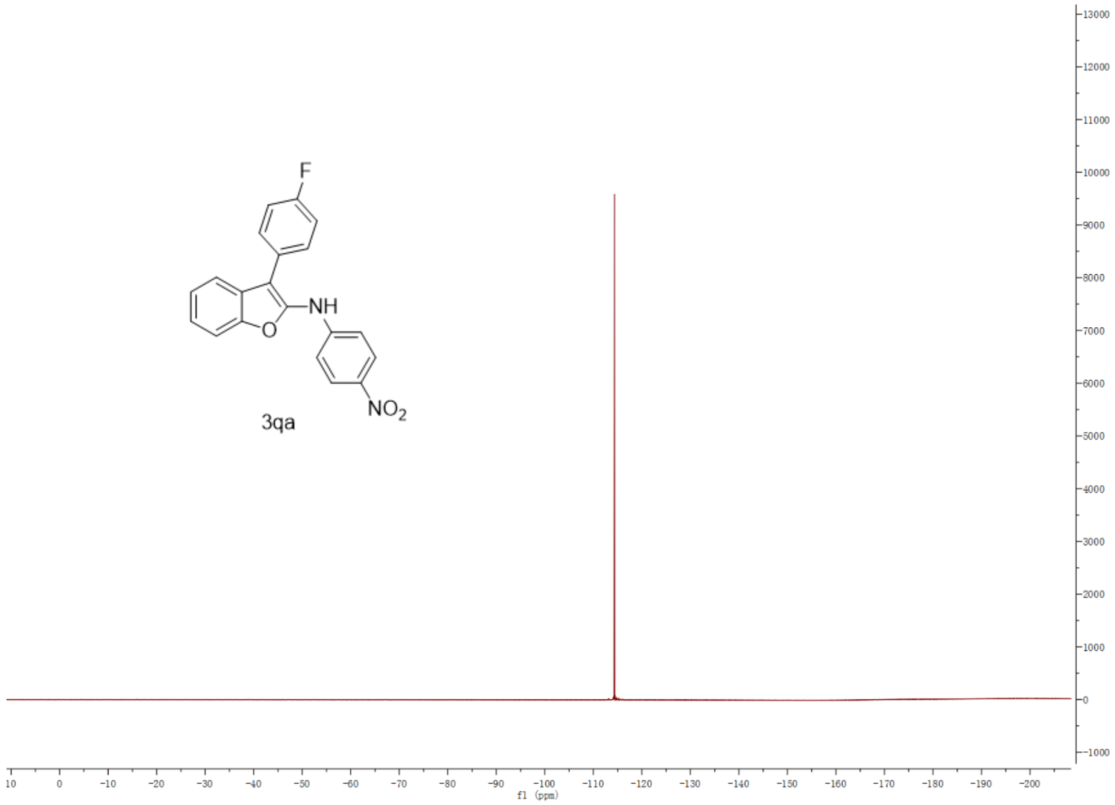

Figure S37. The ^{19}F NMR of compound **3qa**.

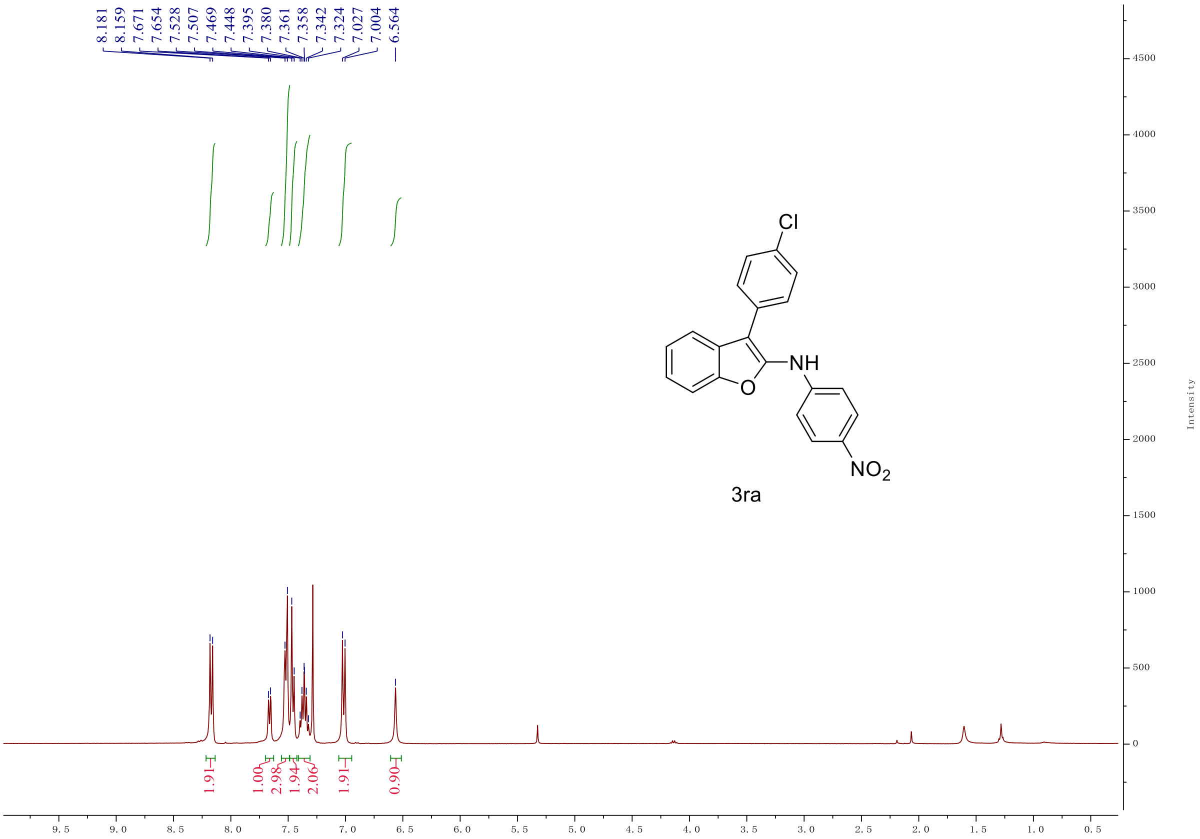

Figure S38. The ^{1}H NMR of compound **3ra**.

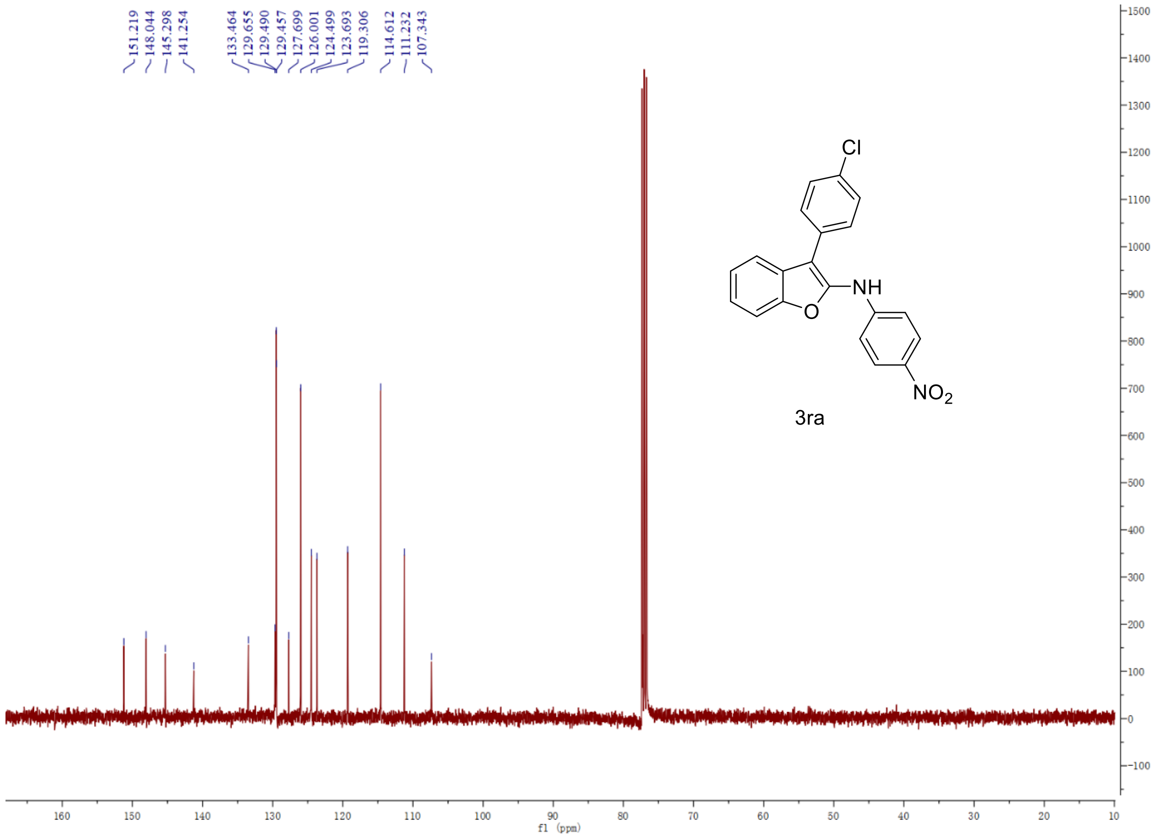

Figure S39. The ^{13}C NMR of compound **3ra**.

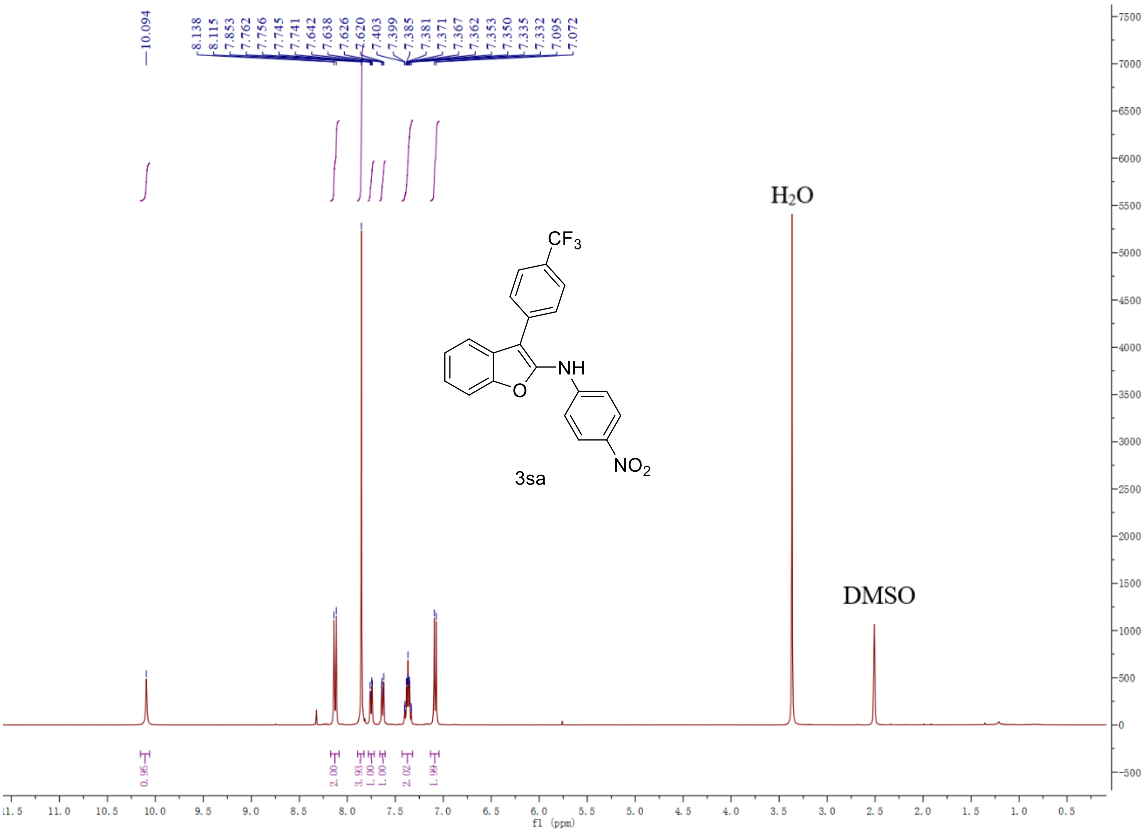

Figure S40. The ^{1}H NMR of compound **3sa**.

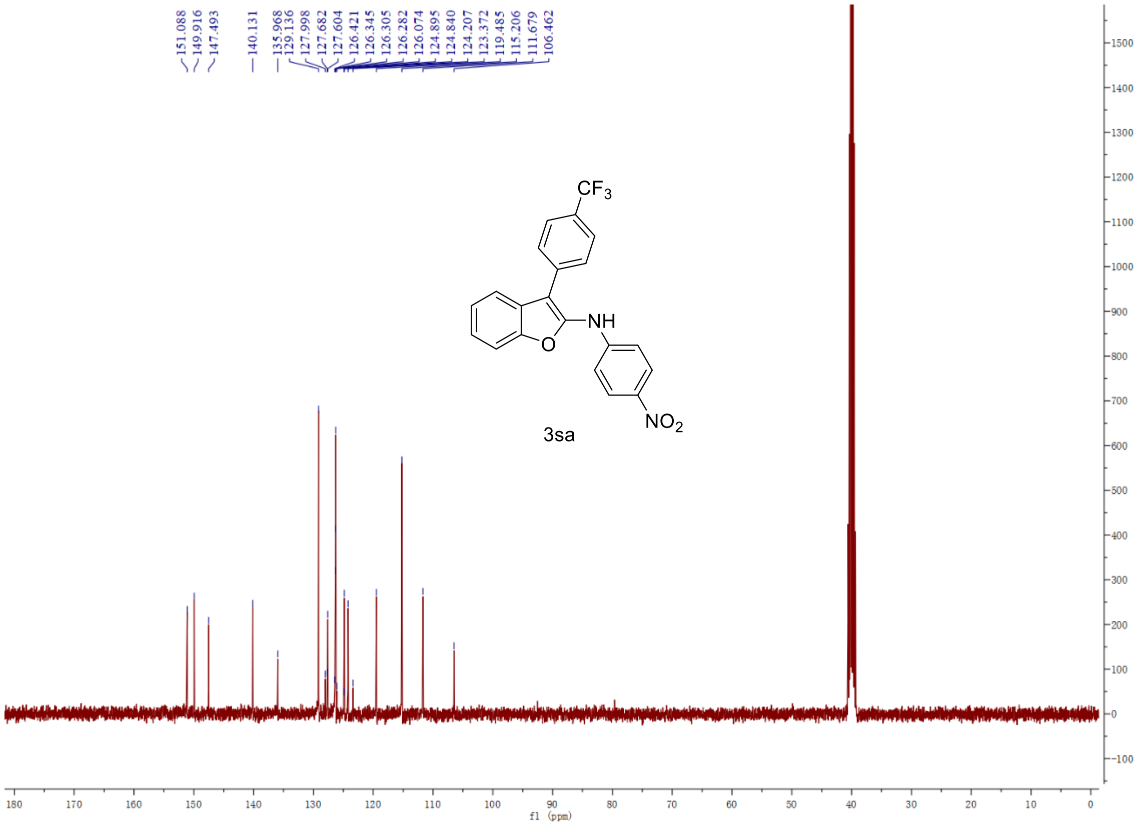

Figure S41. The ^{13}C NMR of compound **3sa**.

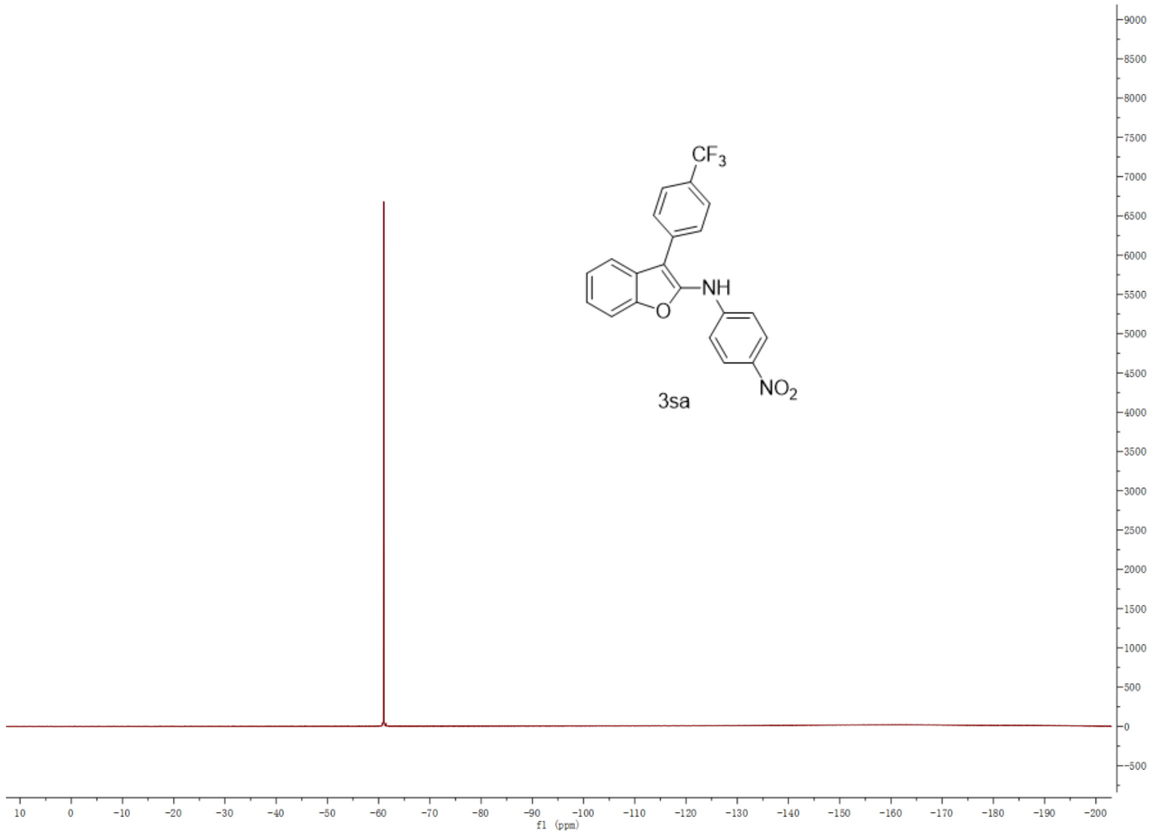

Figure S42. The ^{19}F NMR of compound **3sa**.

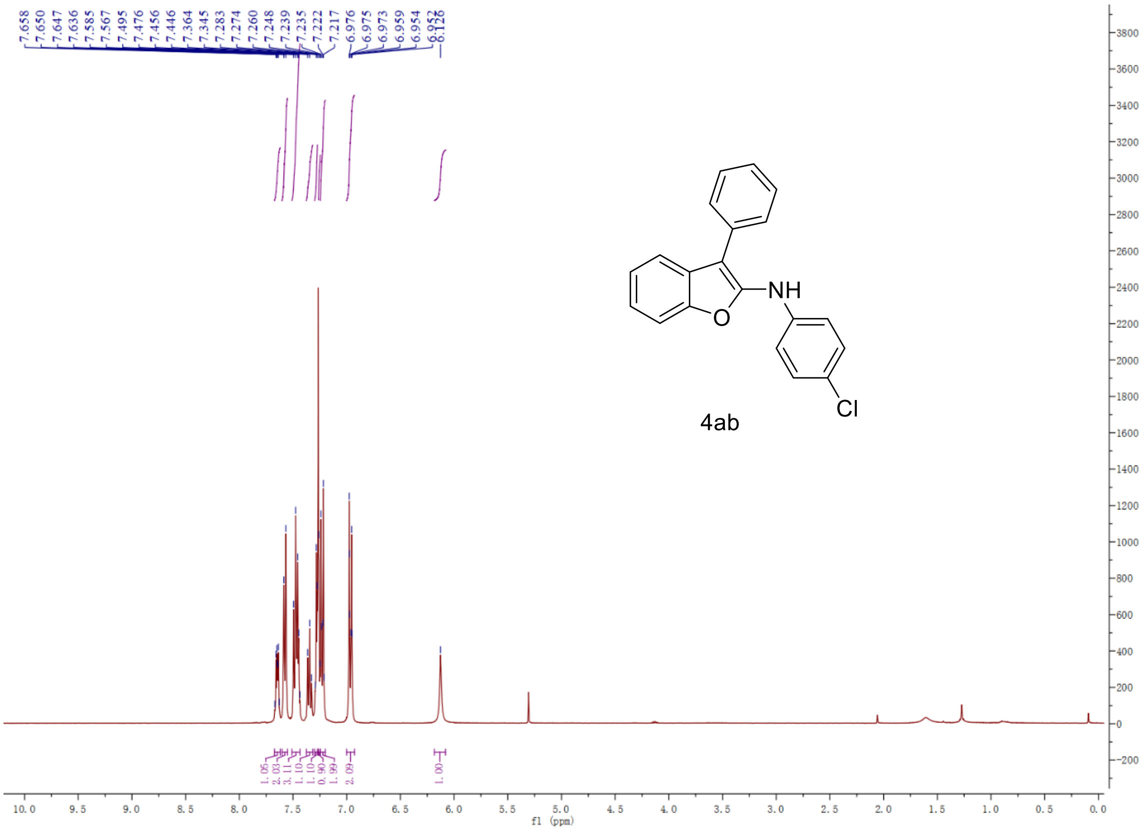

Figure S43. The ^{1}H NMR of compound **4ab**.

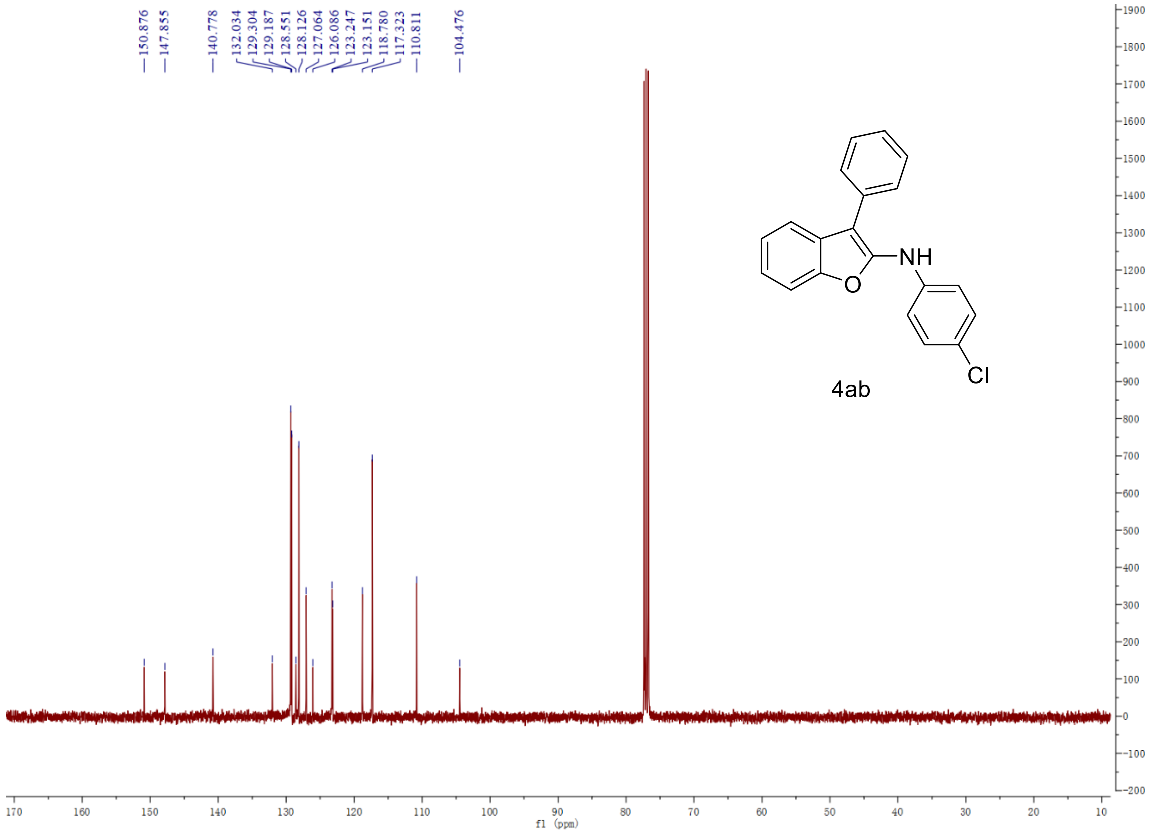

Figure S44. The ^{13}C NMR of compound **4ab**.

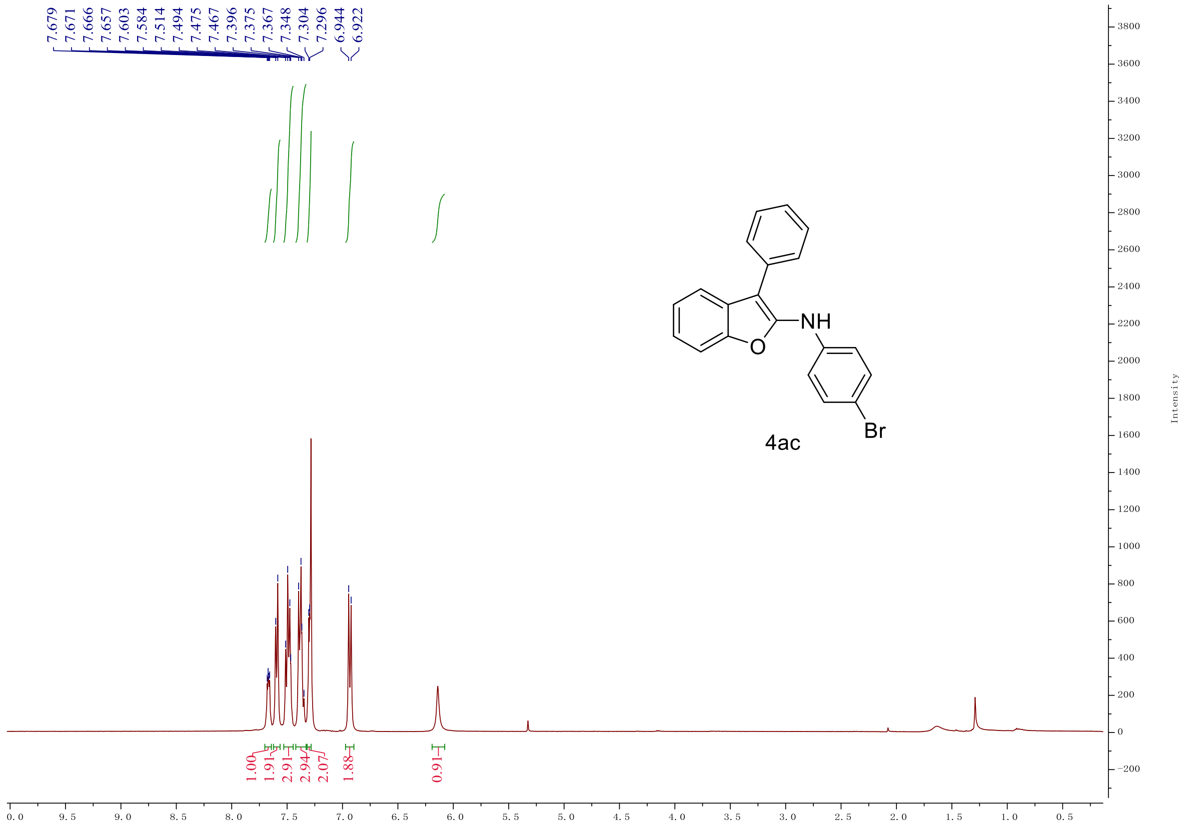

Figure S45. The ^{1}H NMR of compound **4ac**.

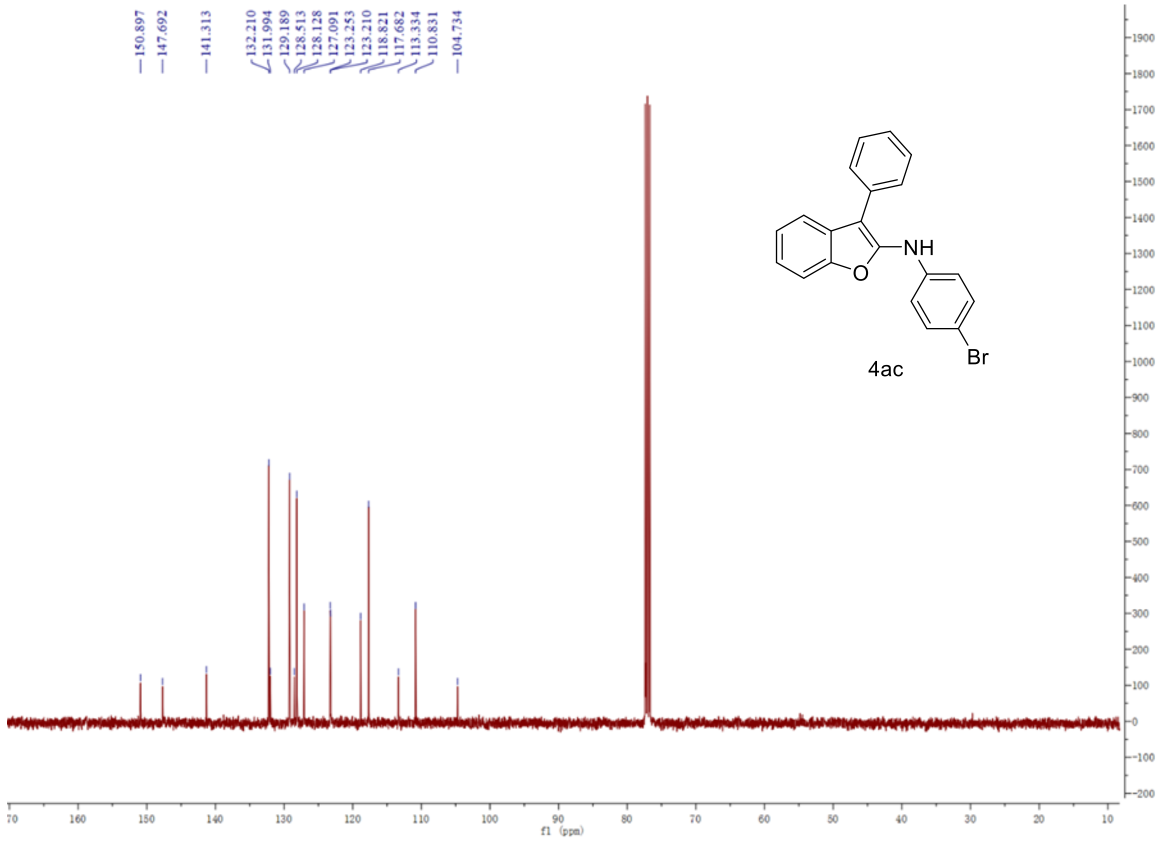

Figure S46. The ^{13}C NMR of compound **4ac**.

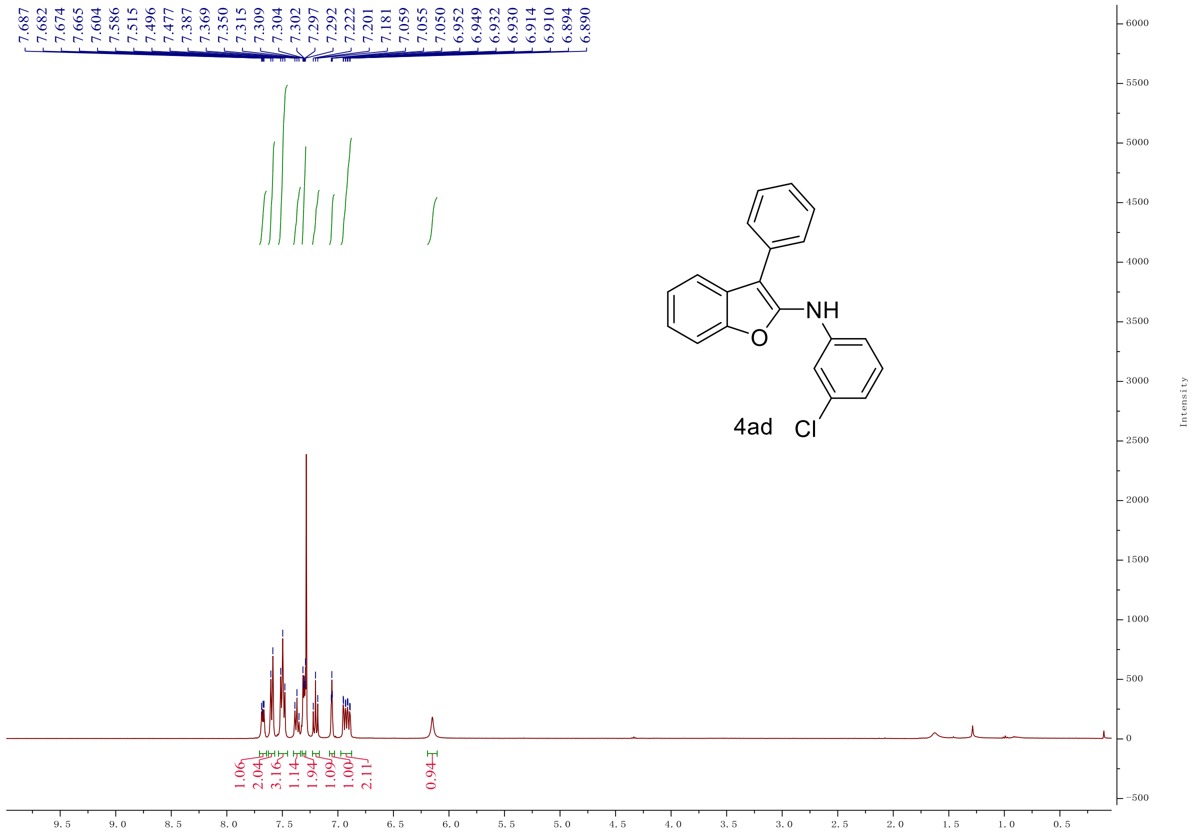

Figure S47. The ^{1}H NMR of compound **4ad**.

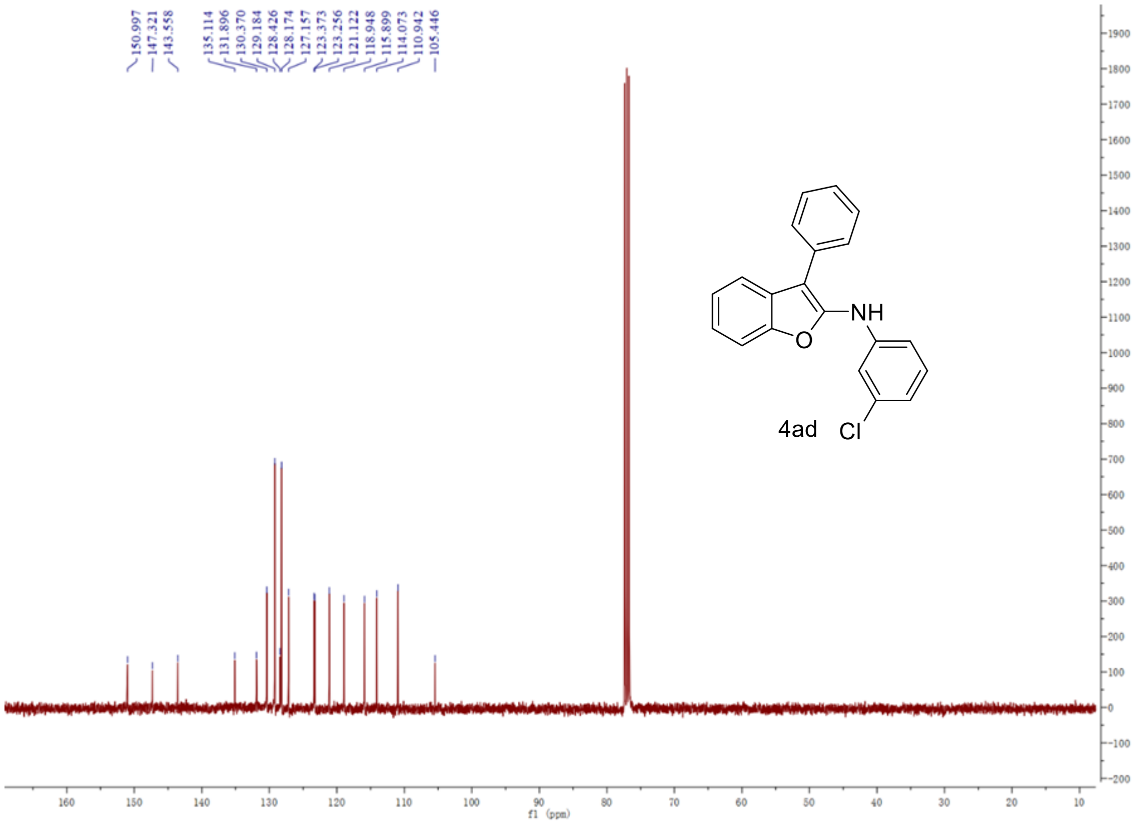

Figure S48. The ^{13}C NMR of compound **4ad**.

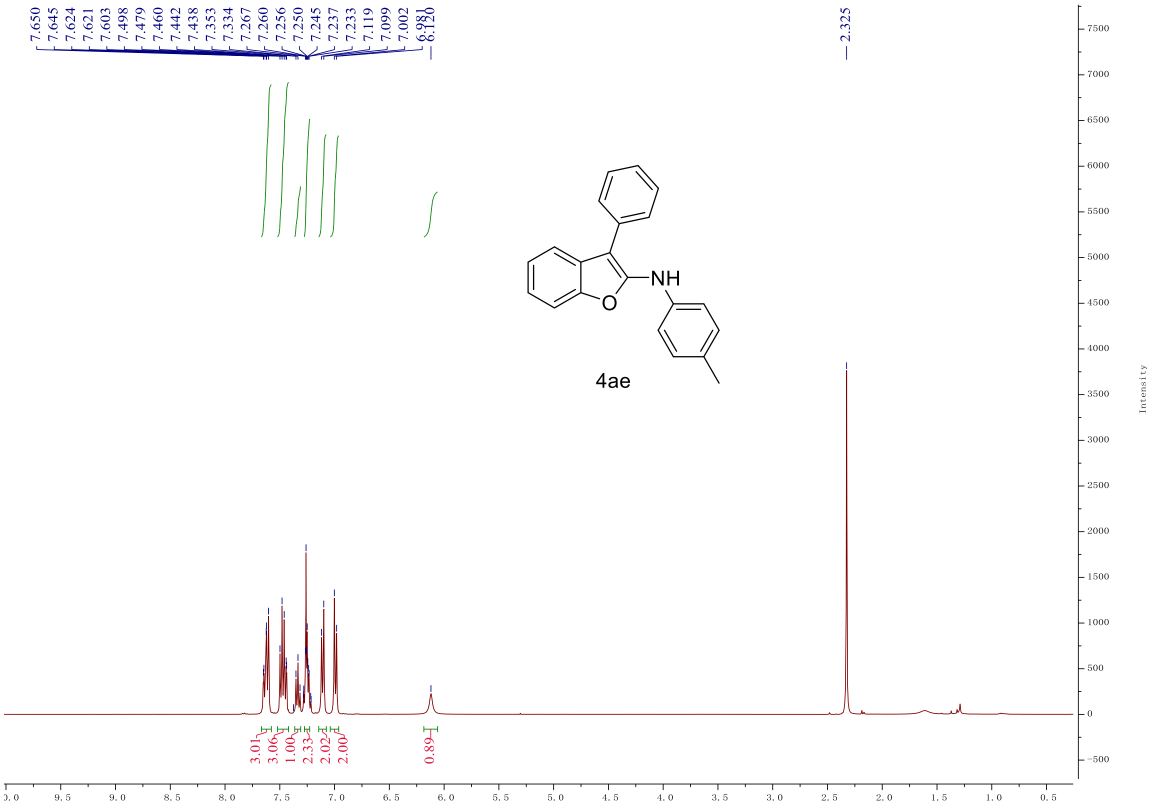

Figure S49. The ^{1}H NMR of compound **4ae**.

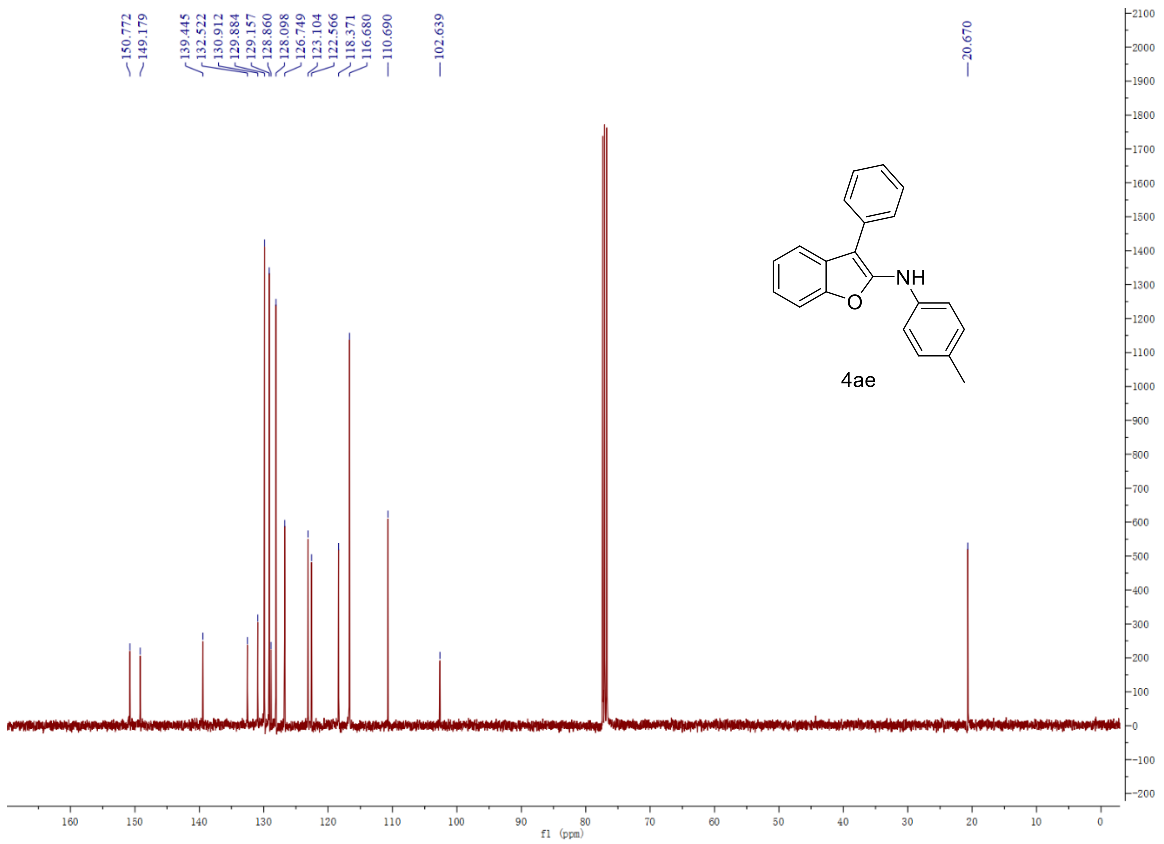

Figure 50. The ^{13}C NMR of compound **4ae**.

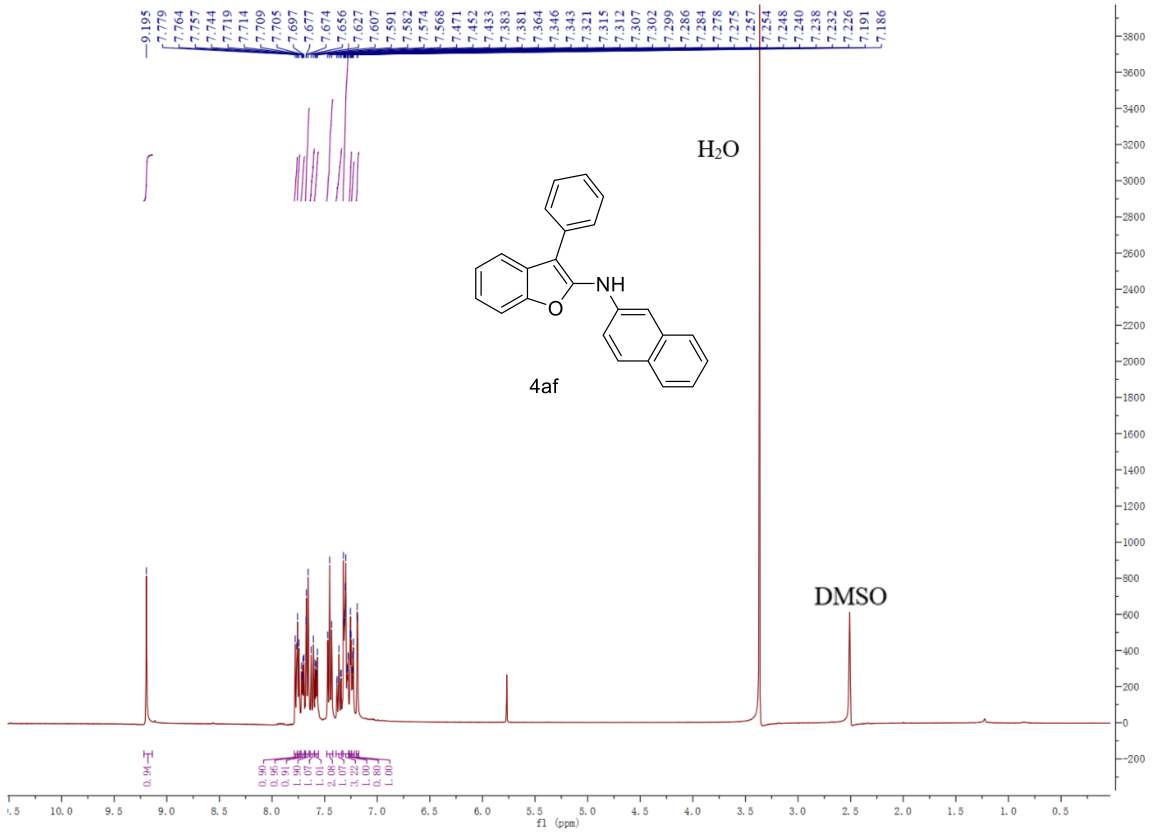

Figure S51. The ^{1}H NMR of compound **4af**.

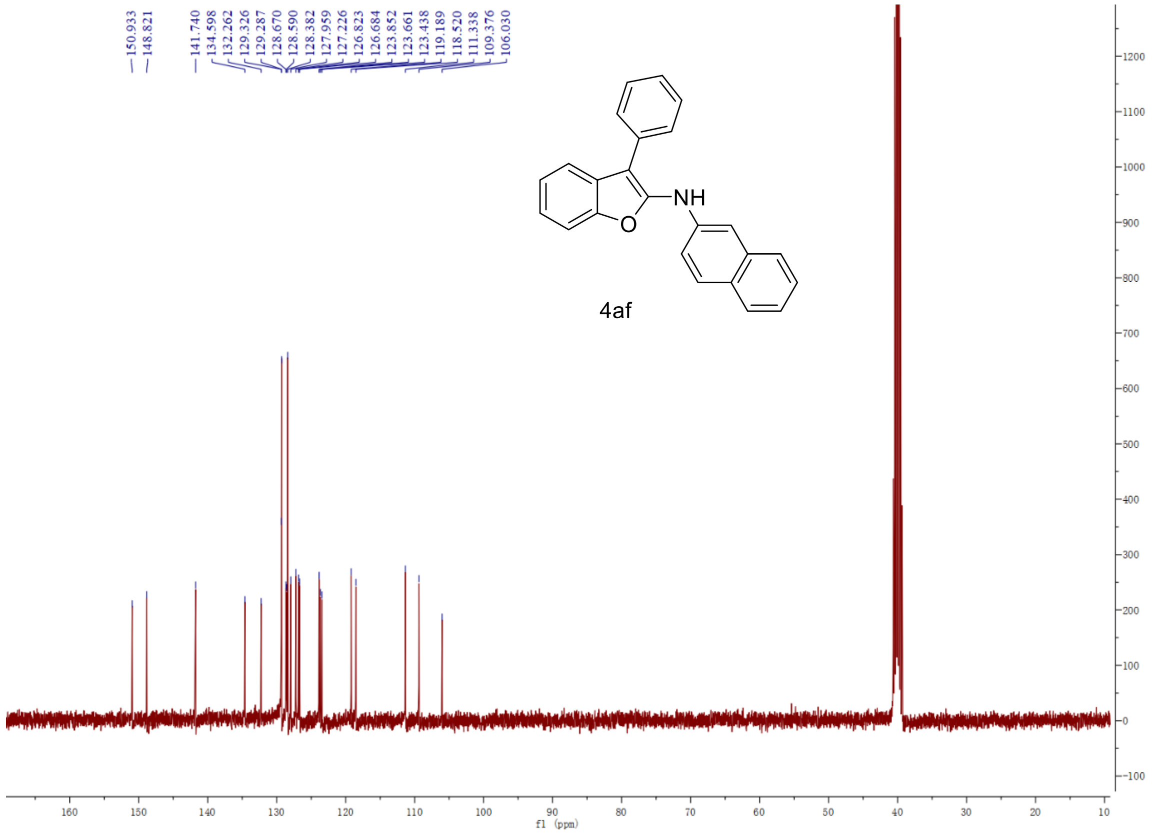

Figure S52. The ^{13}C NMR of compound **4af**.

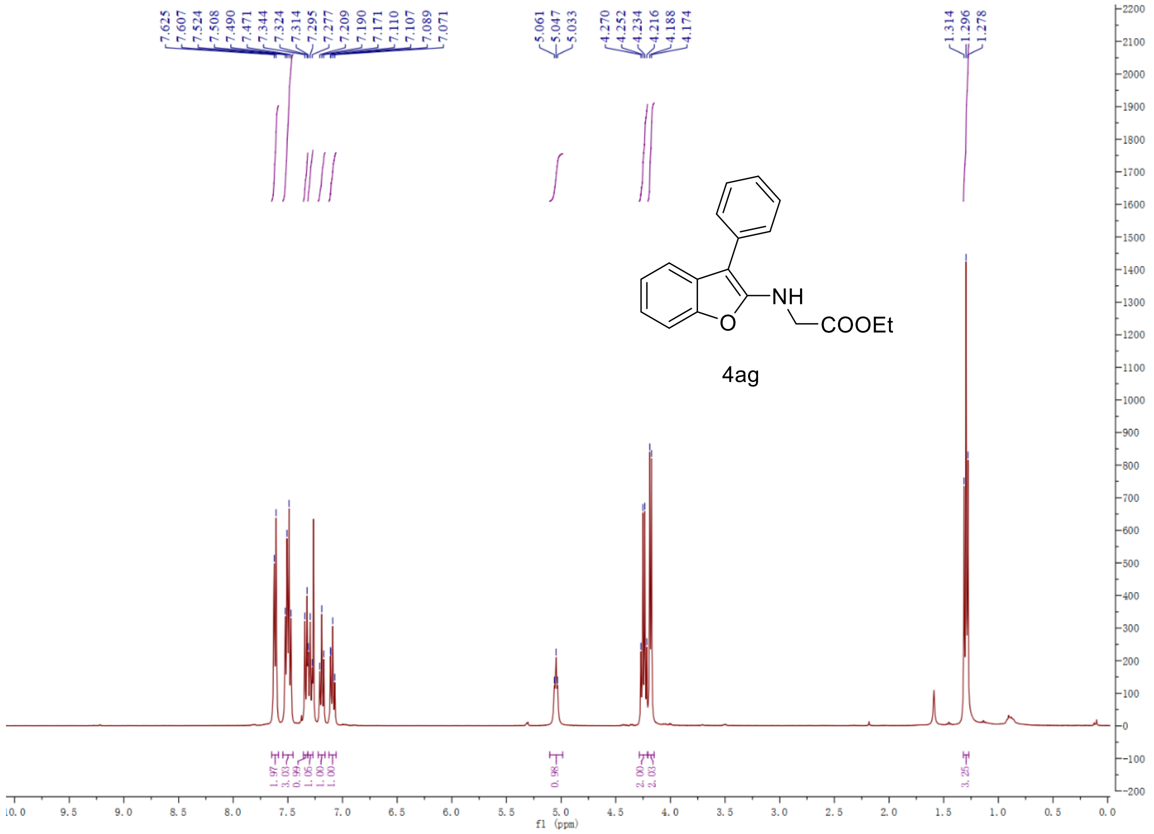

Figure S53. The ^{1}H NMR of compound **4ag**.

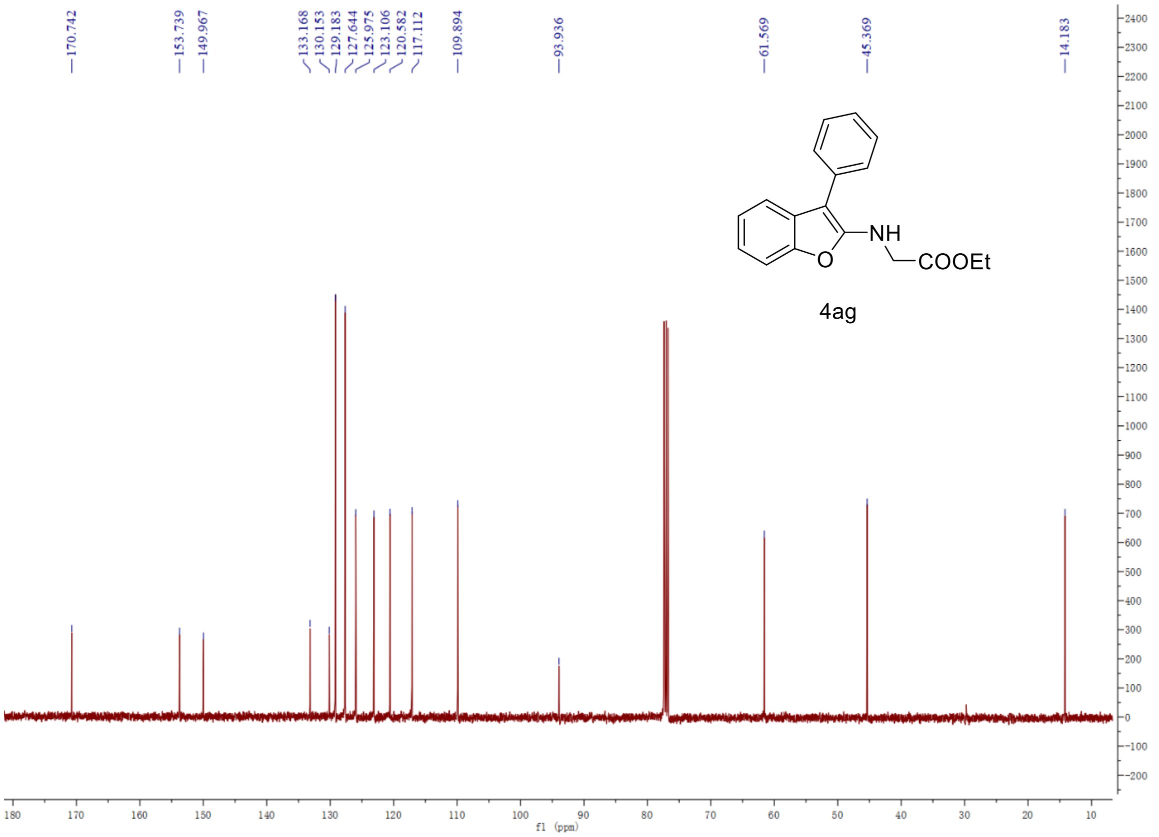

Figure S54. The ^{13}C NMR of compound **4ag**.

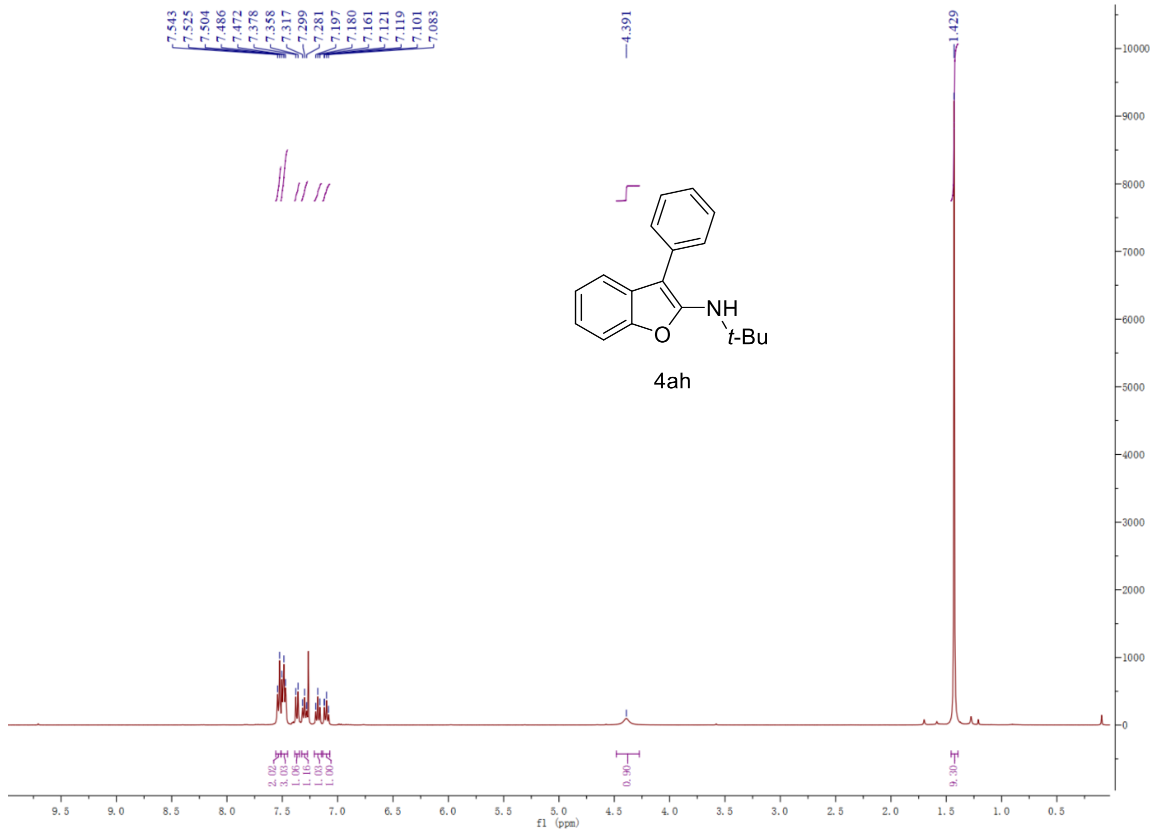

Figure S55. The ^{1}H NMR of compound **4ah**.

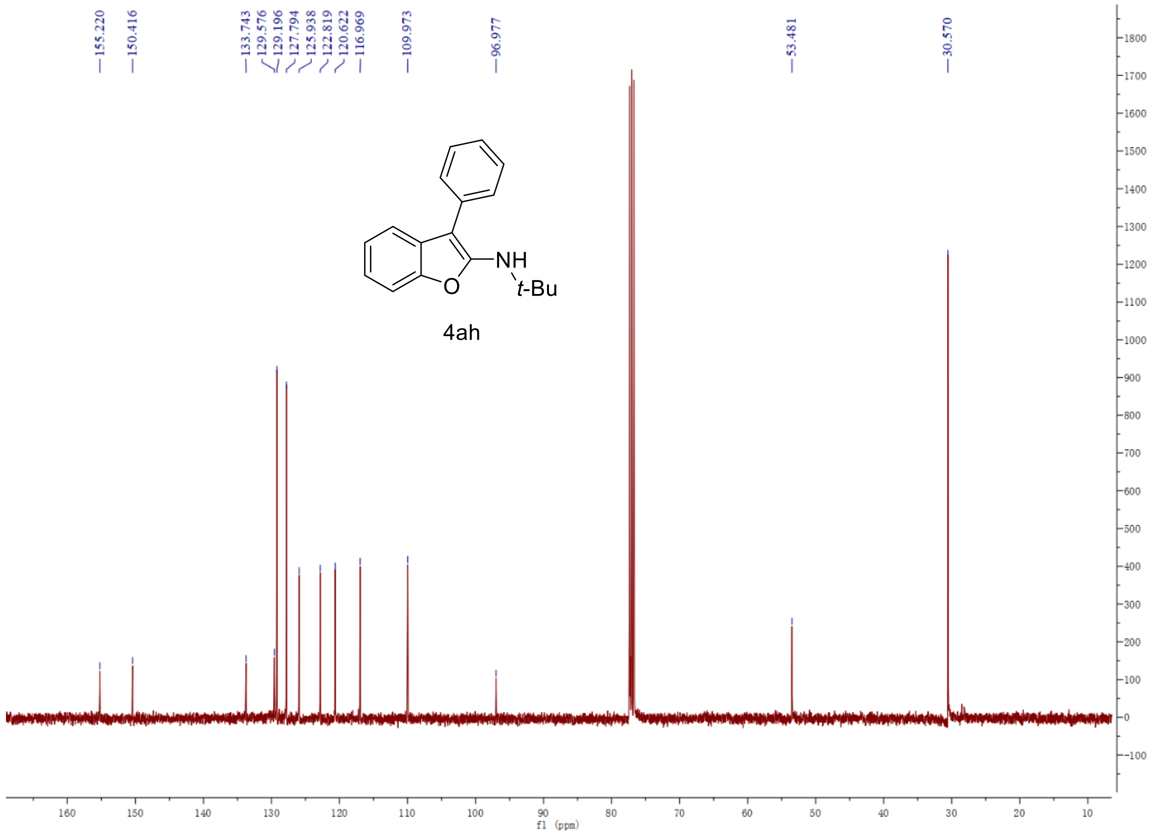

Figure S56. The ^{13}C NMR of compound **4ah**.
